# Homologous recombination promotes non-immunogenic mitotic cell death upon DNA damage

**DOI:** 10.1038/s41556-024-01557-x

**Published:** 2025-01-13

**Authors:** Radoslaw Szmyd, Sienna Casolin, Lucy French, Anna G. Manjón, Melanie Walter, Léa Cavalli, Christopher B. Nelson, Scott G. Page, Andrew Dhawan, Eric Hau, Hilda A. Pickett, Harriet E. Gee, Anthony J. Cesare

**Affiliations:** 1 Genome Integrity Unit, Children’s Medical Research Institute, University of Sydney, Westmead, New South Wales Australia; 2 Radiation Oncology Network, Western Sydney Local Health District, Sydney, New South Wales Australia; 3 Telomere Length Regulation Unit, Children’s Medical Research Institute, University of Sydney, Westmead, New South Wales Australia; 4 Neurological Institute, Cleveland Clinic, Cleveland, OH USA; 5 Westmead Clinical School, University of Sydney, Westmead, New South Wales Australia; 6 Translational Radiation Biology and Oncology Laboratory, Centre for Cancer Research, The Westmead Institute for Medical Research, Westmead, New South Wales Australia

**Keywords:** Double-strand DNA breaks, Homologous recombination, Mitosis, Cell death

## Abstract

Double-strand breaks (DSBs) can initiate mitotic catastrophe, a complex oncosuppressive phenomenon characterized by cell death during or after cell division. Here we unveil how cell cycle-regulated DSB repair guides disparate cell death outcomes through single-cell analysis of extended live imaging. Following DSB induction in S or G2, passage of unresolved homologous recombination intermediates into mitosis promotes non-immunogenic intrinsic apoptosis in the immediate attempt at cell division. Conversely, non-homologous end joining, microhomology-mediated end joining and single-strand annealing cooperate to enable damaged G1 cells to complete the first cell cycle with an aberrant cell division at the cost of delayed extrinsic lethality and interferon production. Targeting non-homologous end joining, microhomology-mediated end joining or single-strand annealing promotes mitotic death, while suppressing mitotic death enhances interferon production. Together the data indicate that a temporal repair hierarchy, coupled with cumulative DSB load, serves as a reliable predictor of mitotic catastrophe outcomes following genome damage. In this pathway, homologous recombination suppresses interferon production by promoting mitotic lethality.

## Abstract (web summary)

Szmyd et al. show that DNA repair pathways impact whether cells with DNA lesions arrest in mitosis. The formation of homologous recombination-driven double Holliday junctions elicits mitotic cell death and suppresses inflammatory signalling.

## Main

Mitotic catastrophe is a phenotypic amalgam induced by genome damage and comprised of mitotic death, interphase death, aberrant cell division and genomic instability^1,2^. During which, mitotic death is associated with mitotic arrest^3–5^ and non-immunogenic BAX- and BAK-dependent apoptosis^1,2^. The cells that survive mitosis with chromosome segregation errors can activate an inflammatory response through recognition of cytosolic DNA or RNA, respectively, by cyclic GMP–AMP synthase (cGAS) or mitochondrial antiviral signalling (MAVS)^4,6–8^. This can potentiate STAT1 activation, interferon regulatory factor 3 (IRF3)-mediated interferon stimulated gene (ISG) expression and extrinsic lethality^6,8^. The mechanisms guiding the diverse outcomes within mitotic catastrophe remain unclear, specifically what distinguishes cells that die in mitosis from those that survive.

Double-strand breaks (DSBs) are repaired through four cell cycle-regulated pathways. Non-homologous end joining (NHEJ) operates throughout G1, S and G2, homologous recombination (HR) and single-strand annealing (SSA) are active in S and G2 and microhomology-mediated end joining (MMEJ) is active outside of G1 (refs. ^9–13^). DNA-dependent protein kinase catalytic subunit (DNA-PKcs) and LIG4 cooperate in NHEJ to ligate DNA ends covalently independent of resection^14^. The other restoration mechanisms require end resection to expose single-strand DNA (ssDNA) sequences used in repair^9,10^. During MMEJ and SSA, homologous ssDNA sequences align, followed by polymerase-mediated fill-in and ligation^15,16^. SSA relies on RAD52 (ref. ^17^), while POLθ is indispensable for MMEJ^18^.

HR utilizes a complementary sequence to template error-free repair^19,20^. Early in HR, the DNA damage response kinase Ataxia telangiectasia and Rad3-related (ATR) phosphorylates PALB2, promoting its interaction with BRCA2 and BRCA2–PALB2 loading at resected DSBs^21,22^. BRCA2 loads RAD51 onto the resected ssDNA^23,24^, and the RAD51-ssDNA nucleofilaments strand-invade the homologous sequence to establish a displacement loop^25^. HR can then proceed through non-crossover synthesis-dependent strand annealing or form the double Holliday junction (dHJ) structural intermediate that is required for crossover repair^26^. RTEL1 suppresses dHJ formation to promote synthesis-dependent strand annealing^27^. dHJs are resolved through BLM–Top3α–RMI1/2 (BTR)-dependent dissolution in interphase, cleaved at the G2/M transition in a manner dependent on SLX4, or cleaved in mitosis dependent on SLX4 or GEN1 (refs. ^28–31^)

Here, we use single-cell analysis of long-duration live imaging to investigate mitotic catastrophe in human cells exposed to ionizing radiation (IR). We found that different cell death outcomes in p53-compromised cells were a function of cell cycle-regulated DSB repair. The formation of toxic HR intermediates emerged as the factor determining whether cells perished through immunogenic or non-immunogenic pathways.

## Results

### Genome damage promotes distinct cell death outcomes

**Fig. 1:**
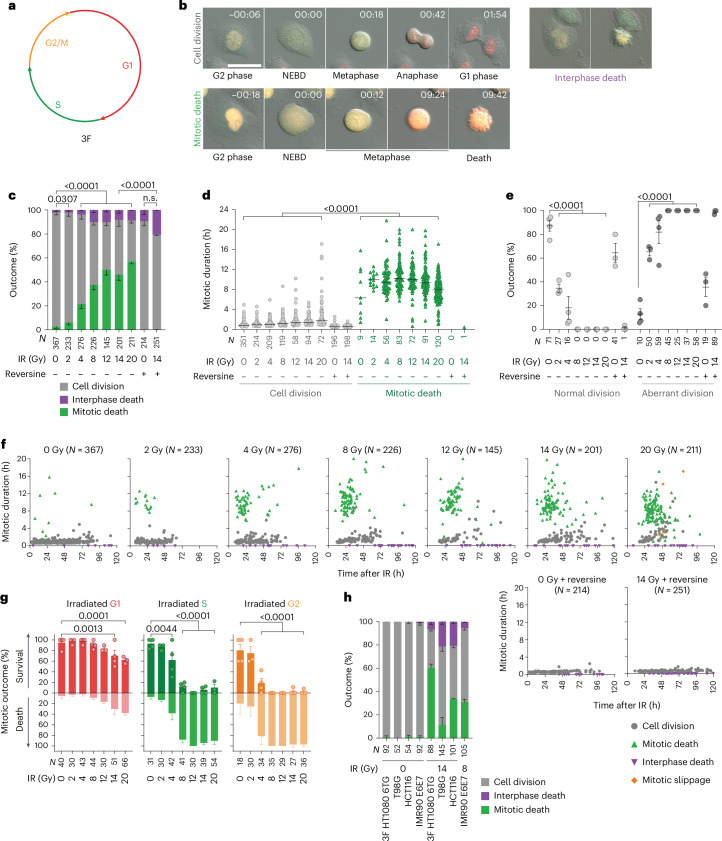
Irradiation induces distinct cell death outcomes. **a**, The 3F schematic^32^. **b**, Stills from 3F HeLa live imaging (representative of *n* = 3; h:min is relative to the nuclear envelope breakdown (NEBD)). Scale bar, 50 µm. **c**–**e**, All cell cycle outcomes (**c**), mitotic duration (**d**) and first completed mitosis outcome (**e**) of 3F HeLa in the 120 h post IR (*n* = 4, 3, 4, 4, 3, 4, 3, 3 and 3 from left to right). Analysis for **c** and **e** includes the replicate mean ± s.e.m. and a two-sided Fisher’s exact test of *N* for mitotic death, and analysis for **d** includes the median and a Kruskal–Wallis uncorrected Dunn’s multiple comparisons test of *N*. **f**, Multi-dimensional representation of **c**. The symbols represent individual outcomes. **g**, First mitosis outcome in 3F HeLa as a function of cell cycle phase at IR (mean ± s.e.m., *n* = 4, 3, 4, 4, 3, 4 and 3 from left to right, two-sided Fisher’s exact test of *N*). **h**, The outcome as in **c** (mean ± s.e.m., *n* = 2). For **a**–**g**, *N* = individual cells across all replicates and *n* = biological replicates. n.s., not significant. Source data

**Extended Data Fig. 1:**
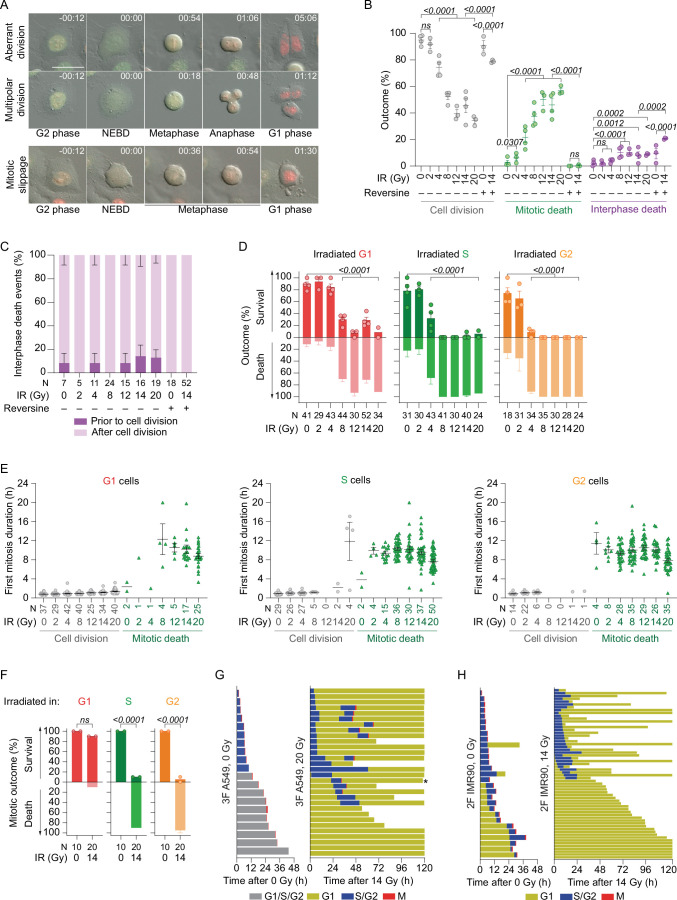
Distinct modes of cell death following lethal genomic damage in p53 compromised cells. **A)** Examples of aberrant mitoses from irradiated 3F HeLa (representative of n = 3). Time is hrs:min relative to nuclear envelope breakdown (NEBD). Scale bar = 50 µm. **B)** Replicate means from Fig. 1c (mean ± SEM left to right n = 4, 3, 4, 4, 3, 4, 3, 3, 3; two-sided Fisher’s exact test of N shown in Fig. 1c). **C)** Interphase lethality from Fig. 1c (mean ± SEM left to right n = 3, 3, 4, 4, 3, 4, 3, 3, 3. **D)** Outcomes over the entire 120-hour live imaging experiment in Fig. 1g as a function of cell cycle phase at irradiation (mean ± SEM left to right of n = 4, 3, 4, 4, 3, 4, 2; two-sided Fisher’s exact test of N). **E)** Mitotic duration from Fig. 1g delineated by outcome and cell cycle phase at IR (mean ± SEM left to right n = 4, 3, 4, 4, 3, 4, 3). **F)** First mitosis outcome in 3F HT1080 6TG as a function of cell cycle phase at irradiation (mean of n = 2; two-sided Fisher’s exact test of N). **G, H)** Cell fate map from 3F A549 (G, n = 1) and 2 F IMR90 (H, n = 2) cultures following mock or IR treatment. Each line represents a single cell relative to IR at T0. For mock-treated cultures, only the first cycle was recorded. For irradiated cultures, cells were tracked for 120 h or until migration from the imaging frame. The asterisk depicts the only cell death event observed. All panels, N = individual cells across all replicates, n = biological replicates, ns = not significant. Source numerical data are available in source data. Source data

We visualized single-cell outcomes in p53-compromised HeLa cells expressing the three-colour FUCCI (3F) cell cycle reporter (Fig. 1a,b and Supplementary Video 1)^32^. The cultures were treated with a single IR fraction and monitored at 6 min intervals for 120 h. The cell cycle phase at IR was noted, and the outcomes were classified as cell division, interphase death or mitotic death (Fig. 1b and Supplementary Video 2). The cell division classification included aberrant mitoses with chromosome segregation errors, micronuclei, multi-polar spindles or mitotic slippage (Extended Data Fig. 1a and Supplementary Video 2).

We observed an IR dose-dependent increase in mitotic death, correlating with prolonged mitosis (Fig. 1c,d and Extended Data Fig. 1b). Mitotic death was significant with ≥2 Gy IR, and ≥8 Gy conferred ubiquitous aberrant mitoses (Fig. 1c,e and Extended Data Fig. 1b). Data visualization in multiple dimensions revealed distinct cell death modes: specifically, a population of cells that arrested and died in the first mitotic attempt 24–48 h post IR, and cells that died predominantly in interphase following one or more aberrant cell divisions (Fig. 1f and Extended Data Fig. 1c). The spindle assembly checkpoint (SAC) prevents mitotic exit until chromosomes correctly align in metaphase^33^. The SAC inhibitor reversine^34^ completely suppressed mitotic arrest and mitotic death in 14-Gy-irradiated cultures, indicating that IR-induced mitotic death is SAC-dependent (Fig. 1c–f and Extended Data Fig. 1b).

We observed a strong correlation between cell cycle phase at IR and cell death mode (Fig. 1g). In asynchronous 3F HeLa, 4 Gy or more induced significant mitotic death in the first attempted cell division within S- and G2-irradiated cells (Fig. 1g). Conversely, G1-irradiated cells predominantly survived their current cell cycle and completed mitosis, to succumb thereafter (Fig. 1g and Extended Data Fig. 1d). Irrespective of the cell cycle phase at IR, mitotic death correlated with mitotic arrest (Extended Data Fig. 1e).

We performed similar experiments in primary two-colour FUCCI (2F) IMR90 fibroblasts^35^, IMR90 HPV16 E6 and E7 (IMR90 E6E7) with compromised p53 and Rb^36^, p53 mutant 3F HT1080 6TG fibrosarcoma and T98G glioblastoma, p53-knockout (KO) HCT116 colorectal carcinoma and p53 wild-type 3F A549 lung adenocarcinoma cells. All irradiated p53-compromised cultures responded similarly to HeLa (Fig. 1h), and 3F HT1080 6TG displayed the same correlation between cell cycle phase at IR and first mitosis outcome (Extended Data Fig. 1f). IR induced G0/G1 arrest in p53 wild-type cultures, preceded by mitotic bypass or cell division in the S/G2-irradiated population (Extended Data Fig. 1g,h). We term mitotic lethality in the first attempted cell division ‘immediate mitotic death’ and cell death following at least one cell division ‘delayed lethality’.

### DSB repair impacts how cells die following genome damage

**Fig. 2:**
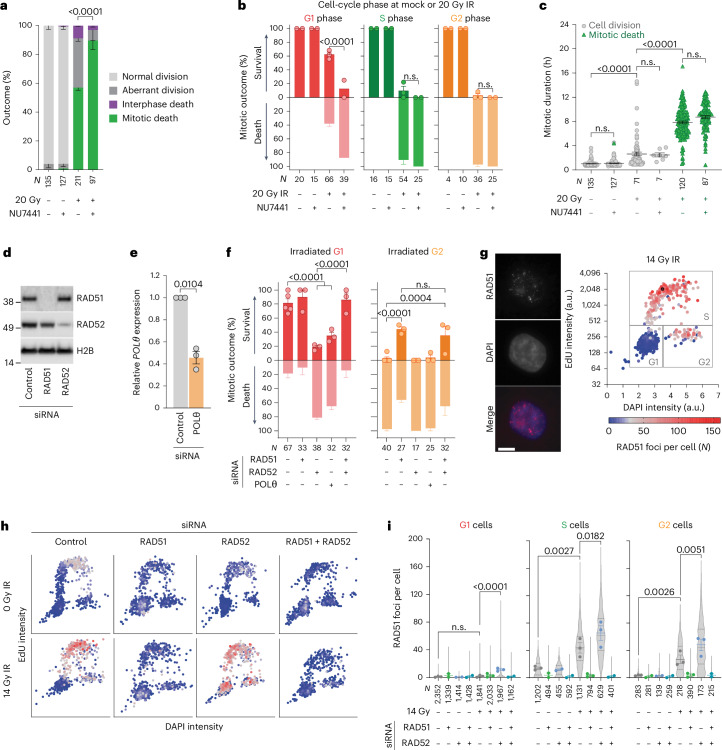
DSB repair pathways guide cell death mode following IR. **a**–**c**, All cell cycle outcomes over 120 h (**a**), first mitosis outcomes (**b**) and all mitotic durations (**c**) for 3F HeLa ± 0.5 µM NU7441. The 20 Gy IR are from Fig. 1, and the irradiated samples in **c** are stratified by outcome (for **a** and **b**, the mean ± s.e.m. *n* = 2, except 20 Gy (*n* = 3), two-sided Fisher’s exact test of *N*; for **c**, mean ± s.e.m. and Kruskal–Wallis uncorrected Dunn’s multiple comparisons test of *N*). **d**, Western blots of whole-cell extracts from 3F HeLa (representative of *n* = 3). **e**, Quantitative PCR with reverse transcription of 3F HeLa (mean ± s.e.m., *n* = 3, two-tailed paired *t*-test, *t* = 9.7, d.f. of 2, 95% confidence interval (CI) −0.79 to −0.30; fold change relative to control siRNA, normalized to 1). **f**, The first mitosis outcome in 3F HeLa (mean ± s.e.m., *n* = 3, except control siRNA (*n* = 5); two-sided Fisher’s exact test of *N*). **g**, Example images and RAD51 foci quantitation from HeLa. Each dot represents a single cell (representative of *n* = 3). Scale bar, 20 µm. **h**,**i**, RAD51 foci in siRNA transfected HeLa ± 14 Gy IR (**h**) and quantitation (**i**) (for **i**, mean ± s.e.m. *n* = 3, 2, 2, 2, 3, 3, 3 and 2 from left to right; violin plots of *N*; the dotted lines are quartiles and the solid lines medians; ordinary one-way ANOVA with Fisher’s Least Significant Difference (LSD) multiple comparisons test). For **a**–**i**, *N* = individual cells across all replicates and *n* = biological replicates. n.s., not significant. Source data

**Extended Data Fig. 2:**
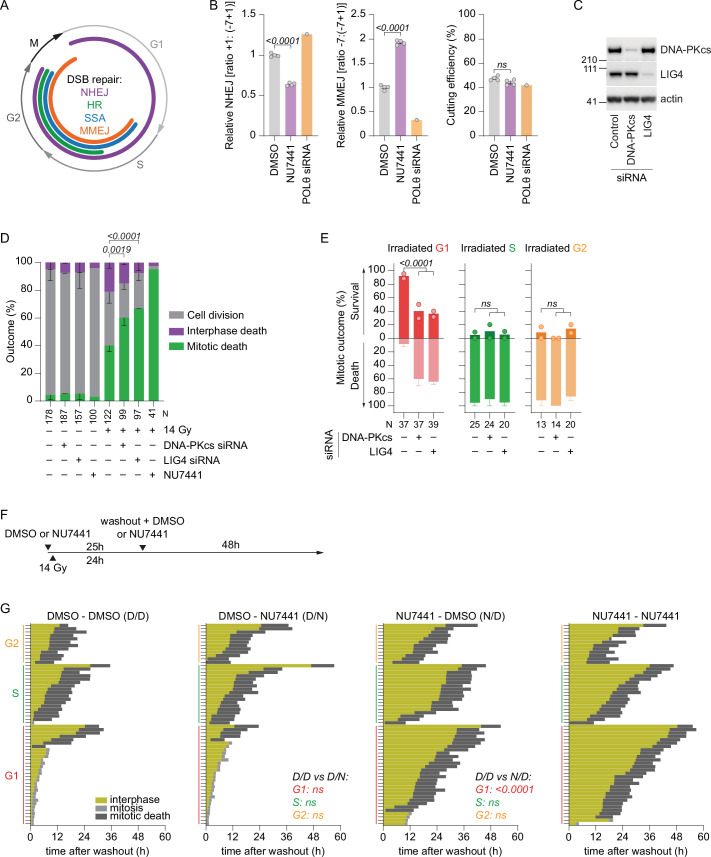
Immediate mitotic death is suppressed by NHEJ. **A)** Active DSB repair pathways relative to cell cycle phase. **B)** TIDE measurement of relative NHEJ ( + 1) and MMEJ (-7) of a Cas9-induced DSB in HeLa^71,72^, normalized to 1 for DMSO-treated samples. Cas9 cutting efficiency on right (mean ± SEM, n = 4, except POLθ siRNA n = 1, unpaired two-tailed t-test; t, df, and 95% CI in Source Data). **C)** Western blots of whole cell extracts derived from 3F HeLa (representative of n = 3). **D)** All cell cycle outcomes in 3F HeLa ± siRNA, 0.5 µM NU7441, and/or 14 Gy IR (mean ± SEM n = 2 except NU7441 positive control n = 1, two-sided Fisher’s exact test of N). **E)** First cell cycle outcome from (D) (mean ± SEM n = 2, two-sided Fisher’s exact test of N). **F, G)** Experimental timing (F) and cell fate maps (G) of asynchronous and 14 Gy irradiated 3F HeLa ± DMSO or 0.5 µM NU7441. Each line is a single cell, sorted by cell cycle phase at irradiation (n = 2, all cells compiled, two-sided Fisher’s exact test of N = 60). All panels, N = individual cells across all replicates, n = biological replicates, ns = not significant. Source numerical data and unprocessed blots are available in source data. Source data

Correlation between cell death mode and cell cycle phase at IR suggested a connection to DSB repair (Extended Data Fig. 2a). We suppressed NHEJ with the DNA-PKcs inhibitor NU7441 or DNA-PKcs or LIG4 short interfering RNA^37^ (Extended Data Fig. 2b,c). This did not affect mock-irradiated cultures, nor S- or G2-irradiated cells within asynchronous 3F HeLa. Targeting DNA-PKcs or LIG4 did, however, confer immediate mitotic arrest and death in G1-irradiated cells (Fig. 2a–c, Extended Data Fig. 2d,e and Supplementary Video 3). We irradiated asynchronous 3F HeLa in the presence or absence of NU7441 and 24 h later added or washed NU7441 from the media (Extended Data Fig. 2f). G1-irradiated cells in vehicle pre-treated cultures predominantly escaped immediate mitotic death, and adding NU7441 24 h post IR did not impact first mitotic survival. Conversely, pre-treating with NU7441 conferred immediate mitotic death in the G1-irradiated population, even when NU7441 was removed 24 h later (Extended Data Fig. 2g).

**Extended Data Fig. 3:**
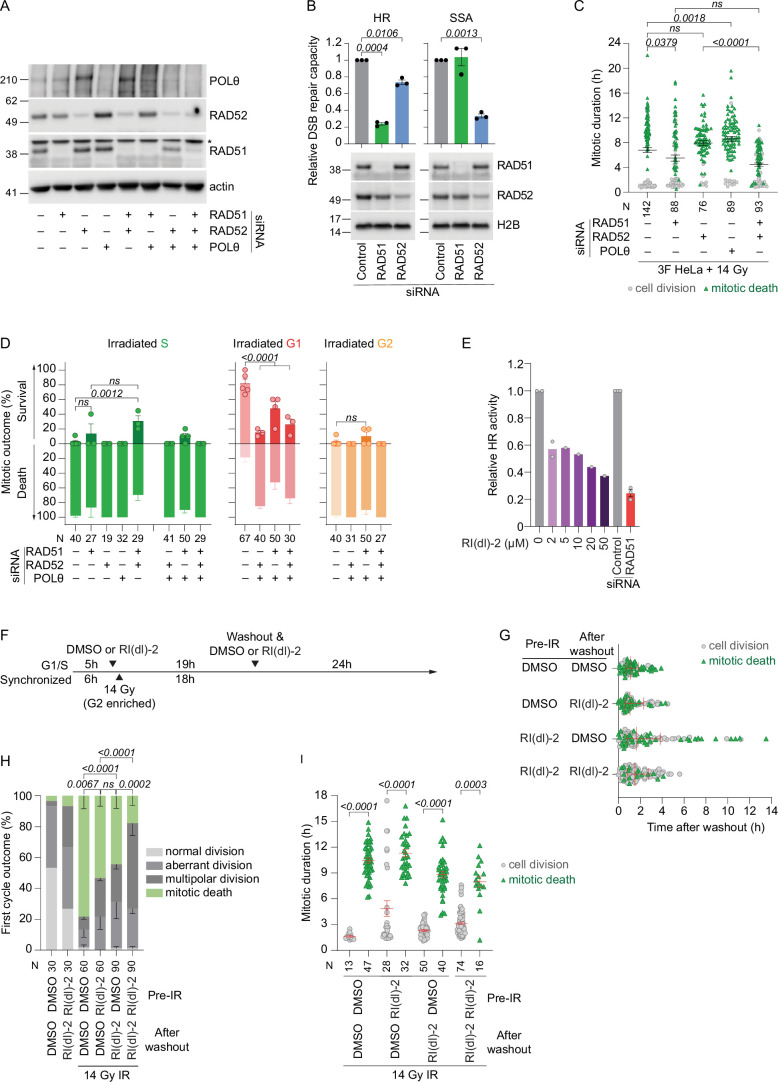
RAD51 promotes mitotic arrest and mitotic death in irradiated cells. **A)** Western blots of 3F HeLa whole cell extracts (*non-specific band, n = 1). **B)** HR and SSA reporters^17,73^ in siRNA and I-SceI transfected HeLa normalized to 1. (mean ± SEM n = 3, two-tailed paired t-test; t, df, and 95% CI in Source Data). Western blots of whole cell extracts from experimental cultures shown below (representative of n = 3). **C)** First mitosis duration of G1, S, and G2 irradiated 3F HeLa cultures from (D) and Fig. 2f combined (n = 3, except for control siRNA n = 5; mean ± SEM and Kruskal-Wallis uncorrected Dunn’s multiple comparisons test of N). **D)** Fist mitosis outcome in 14 Gy irradiated 3F HeLa (mean ± SEM, n = 3 except for control siRNA n = 5, and RAD51/POLθ siRNA n = 4; two-sided Fisher’s exact test of N). 14 Gy control siRNA samples for G1 and G2 irradiated cells are from Fig. 2f. **E)** HR activity as in (B) in siRNA transfected or RI(dl)-2 treated HeLa (mean ± SEM n = 2, 2, 1, 1, 1, 1, 3, 3 left to right). **F)** Experimental timing in (G-I). Mitotic outcomes were scored following media change. **G-I)** Time of mitotic entry (**G**), first mitotic outcome (**H**), and mitotic duration (G) for 3F HeLa treated with DMSO, 50 µM Rl(dl)-2, and 14 Gy IR (n = 1, 1, 2, 2, 3, 3 left to right in H; G, median ± interquartile range of N; H, mean ± SEM of n; two-sided Fisher’s exact test of N; **I**, mean ± SEM and Kruskal-Wallis uncorrected Dunn’s multiple comparisons test of N). All panels, N = individual cells across all replicates, n = biological replicates, ns = not significant. Source numerical data and unprocessed blots are available in source data. Source data

We targeted HR, SSA or MMEJ in 3F Hela by, respectively, depleting RAD51, RAD52 or POLθ and irradiated asynchronous cultures with 14 Gy (Fig. 2d,e, Extended Data Figs. 2b and 3a,b and Supplementary Video 4). RAD52 or POLθ depletion failed to influence the outcome of S/G2-irradiated cells but instead conferred immediate mitotic arrest and death in the G1-irradiated population (Fig. 2f and Extended Data Fig. 3c,d). Conversely, singular RAD51 knockdown rescued immediate mitotic arrest and death in G2-irradiated cells and co-depleting RAD51 rescued the enhanced immediate mitotic death conferred by RAD52 depletion (Fig. 2f and Extended Data Fig. 3c,d). RAD51 co-depletion did not rescue mitotic death in POLθ-depleted cells, potentially reflecting known RAD51 and POLθ synthetic lethality^38^ (Extended Data Fig. 3d). Under all conditions, mitotic arrest and death correlated (Extended Data Fig. 3c).

RAD51 and RAD52 compete in DSB repair^8,14,39^. RAD51 foci were present in non-irradiated S-phase cells consistent with spontaneous replication stress^40^ and in 14-Gy-irradiated S/G2 cells (Fig. 2g–i). RAD52 depletion conferred elevated RAD51 foci in 14-Gy-irradiated G1, S and G2 cells, consistent with premature HR engagement in RAD52-depleted cultures that experience a significant increase in RAD51-dependendent immediate mitotic death (Fig. 2f–i).

We also targeted HR with RI(dl)-2, a RAD51 inhibitor that suppresses displacement loop formation (Extended Data Fig. 3e)^41^. The 3F HeLa cells were G2-enriched through a double thymidine block, released and confirmed by live imaging of G2 predominance 6 h later. The cultures were dimethylsulfoxide (DMSO) or RI(dl)-2 treated 5 h post release, irradiated 6 h post release and the media exchanged 24 h post release. The cells typically entered mitosis within the following 4 h (Extended Data Fig. 3f,g). RI(dl)-2 equally rescued immediate mitotic death when added before IR and washed out or when first added 18 h post IR. Maintaining RI(dl)-2 in the media throughout conferred the greatest mitotic death rescue (Extended Data Fig. 3h,i). RAD51-dependent strand invasion at any time before mitotic entry can therefore promote immediate mitotic death, whereas DNA-PKcs, LIG4, POLθ and RAD52 cooperate to enable first cell cycle survival.

### HR promotes immediate mitotic death

**Fig. 3:**
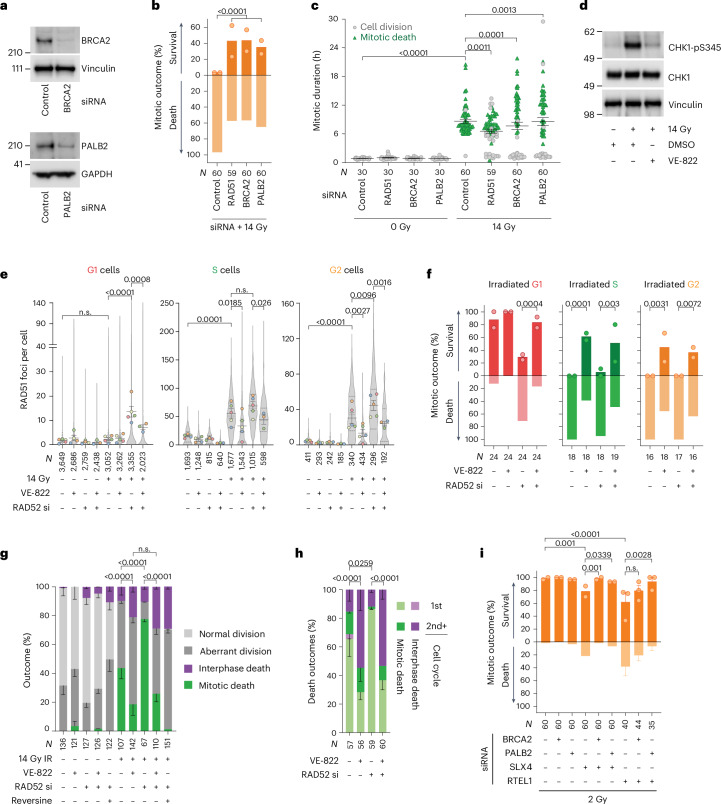
Recombination intermediates potentiate mitotic death. **a**, Western blots of whole-cell extracts from 3F HeLa (representative of *n* = 2). **b**,**c**, First mitosis outcome (**b**) and mitotic duration (**c**) in G2-enriched 3F HeLa treated with 14 Gy IR (for **b**, the mean *n* = 2, two-sided Fisher’s exact test of *N*; for **c**, mean ± s.e.m. and two-tailed Kolmogorov–Smirnov test of *N*). **d**, Western blots of whole-cell extracts collected 1 h post IR from HeLa ± 0.2 µM VE-822 (*n* = 1). **e**, RAD51 foci in HeLa ± siRNA (si) and/or 0.2 µM VE-822 (mean ± s.e.m. of *n* = 5, 4, 4, 3, 5, 5, 5 and 3 from left to right; violin plots of *N*, the dotted lines are quartiles and the solid lines medians; ordinary one-way ANOVA with Fisher’s LSD multiple comparisons test). All VE-822 negative conditions include replicates from Fig. 2i. **f**,**g**, First mitosis (**f**) and all cell cycle outcomes over 120 h (**g**) in 3F HeLa ± siRNA, 0.2 µM VE-822, 0.5 µM reversine and/or 14 Gy IR (for **f**, mean *n* = 2, two-sided Fisher’s exact test of *N*; for **g**, the mean ± s.e.m. *n* = 2, two-sided Fisher’s exact test of *N* for mitotic death). **h**, Cell death from **g** (mean ± s.e.m. *n* = 2, two-sided Fisher’s exact test of *N*). **i**, First mitosis in G2-enriched 3F HeLa following 2 Gy IR (mean *n* = 2, except RTEL1 siRNA ± BRCA2/PALB2 siRNA mean ± s.e.m. (*n* = 3); two-sided Fisher’s exact test of *N*). For **a**–**i**, *N* = individual cells across all replicates and *n* = biological replicates. n.s., not significant. Source data

**Extended Data Fig. 4:**
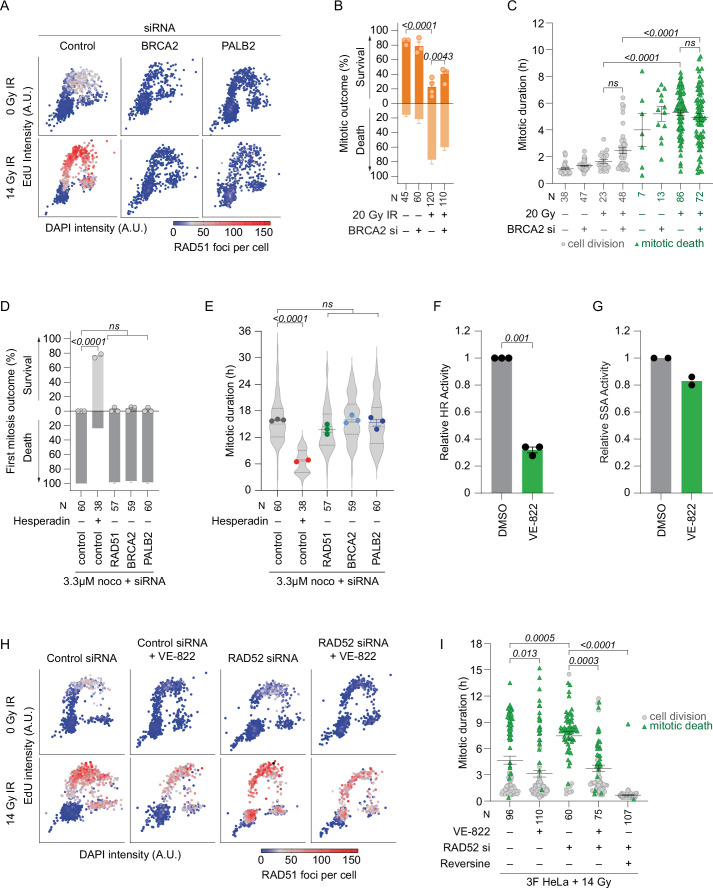
BRCA2, PALB2, and ATR promote immediate mitotic death following IR. **A)** RAD51 foci in HeLa ± 14 Gy IR and/or siRNA as shown in Fig. 2g (n = 1). **B, C)** First mitosis outcome (**B**) and duration (**C**) in G2 enriched 3F HT1080 6TG ± 20 Gy IR and/or siRNA (B, mean ± SEM n = 3, 3, 4, 4 left to right, two-sided Fisher’s exact test of N; C, mean ± SEM and Kruskal-Wallis uncorrected Dunn’s multiple comparisons test of N). **D, E)** Mitotic outcome (**D**) and duration (**E**) for HeLa ± siRNA, 3.3 µM Nocodazole, and/or 0.5 µM Hesperadin (D, mean ± SEM n = 3, except Hesperadin n = 2, two-sided Fisher’s exact test of N; E, mean ± SEM of n, violin plots depict N, dotted lines mark quartiles and solid lines represent medians; Kruskal-Wallis Dunn’s multiple comparisons test of N). **F, G)** HR (**F**) or SSA (**G**) I-SceI repair reporters in HeLa cells ± 0.2 µM VE-822 and normalized to 1 (F, mean ± SEM n = 3, two-tailed paired t-test, t = 31.6, df = 2, 95% CI = 0.59 to 0.77; G, mean n = 2). Samples were collected 48 h after I-SceI transfection and 40 h after VE-822 addition. **H)** RAD51 foci in HeLa ± siRNA, 0.2 µM VE-822, and/or 14 Gy IR as quantified in Fig. 3e (representative, see Fig. 3e legend for n). **I)** Mitotic duration of irradiated 3F HeLa cultures from Fig. 3g (mean ± SEM and Kruskal-Wallis uncorrected Dunn’s multiple comparisons test of N from n = 2). All panels, N = individual cells across all replicates, n = biological replicates, ns = not significant. Source numerical data are available in source data. Source data

BRCA2 or PALB2 depletion prevented RAD51 foci formation and rescued immediate mitotic arrest and death in G2-enriched and 14-Gy-irradiated 3F HeLa and 3F HT1080 6TG (Fig. 3a–c and Extended Data Fig. 4a–c). Unlike direct inhibition of the SAC regulator aurora kinase B with hesperadin^42^, RAD51, BRCA2 or PALB2 depletion did not rescue mitotic arrest and death in 3F HeLa treated with the microtubule poison nocodazole (Extended Data Fig. 4d,e). RAD51, PALB2 or BRCA2, therefore, do not directly regulate the SAC.

ATR regulates diverse activities including HR^21^. We suppressed ATR activity with VE-822 (ref. ^43^), as evidenced by diminished CHK1 phosphorylation (Fig. 3d). VE-822 strongly suppressed HR while only mildly inhibiting SSA (Extended Data Fig. 4f,g). In asynchronous 14-Gy-irradiated 3F HeLa, VE-822 suppressed RAD51 foci and immediate mitotic arrest and death, and increased delayed interphase lethality (Fig. 3e–h and Extended Data Fig. 4h,i). This was true for S- and G2-irradiated cells from control siRNA samples and in G1-, S- and G2-irradiated cells in RAD52-depleted cultures. Reversine abolished mitotic arrest and death in irradiated RAD52-depleted cells, consistent with SAC-dependent mitotic lethality (Fig. 3g).

**Extended Data Fig. 5:**
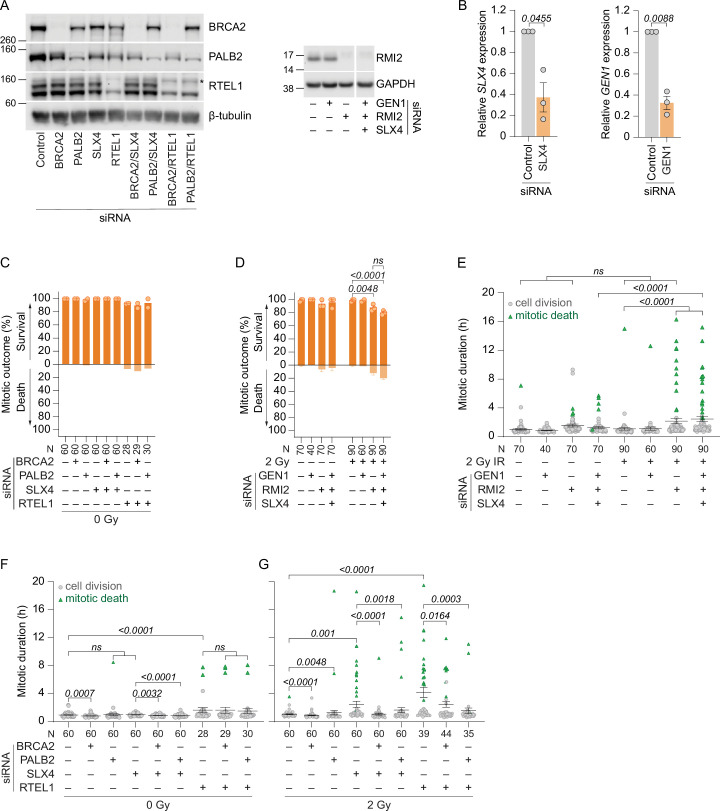
BTR and SLX4 promote mitotic survival following low dose IR. **A)** Western blots of whole cell extracts from 3F HeLa. The asterisk denotes a non-specific band (representative of n = 2). **B)** RT-qPCR of siRNA transfected 3F HeLa (n = 3, two-tailed paired t-test t, df, and 95% CI are shown in Source Data). **C, D)** First mitosis outcome in siRNA transfected and G2 enriched 3F HeLa ± 2 Gy IR (mean ± SEM n = 2 for (**C**) and 3 for (**D**), two-sided Fisher’s exact test of N). **E-G)** Mitotic duration for the cells in Extended Data Fig. 5D (**E**), Extended Data Fig. 5C (**F**), and Fig. 3i (**G**) (mean ± SEM and Kruskal-Wallis uncorrected Dunn’s multiple comparisons test of N). All panels, N = individual cells across all replicates, n = biological replicates, ns = not significant. Source numerical data and unprocessed blots are available in source data. Source data

Downstream of BRCA2 and PALB2-dependent strand invasion, RTEL1 suppresses dHJ formation^27^, and dHJs are resolved through mechanisms dependent on RMI2, SLX4 or GEN1 (refs. ^28–31^). We depleted these factors in 3F HeLa, enriched for G2 and 2 Gy irradiated. RTEL1, RMI2 and/or SLX4 depletion increased immediate mitotic arrest and death in G2-irradiated cells, whereas GEN1 siRNA had no effect (Fig. 3i and Extended Data Fig. 5a–g). Co-depleting BRCA2 and/or PALB2 with RTEL1 or SLX4 rescued immediate mitotic arrest and death (Fig. 3i and Extended Data Fig. 5f,g). Collectively, these data are consistent with SAC-dependent mitotic arrest and death following IR resulting from the passage of ATR-dependent HR intermediates, most probably dHJs, into mitosis.

### Chromosome rearrangements correlate with mitotic survival

**Fig. 4:**
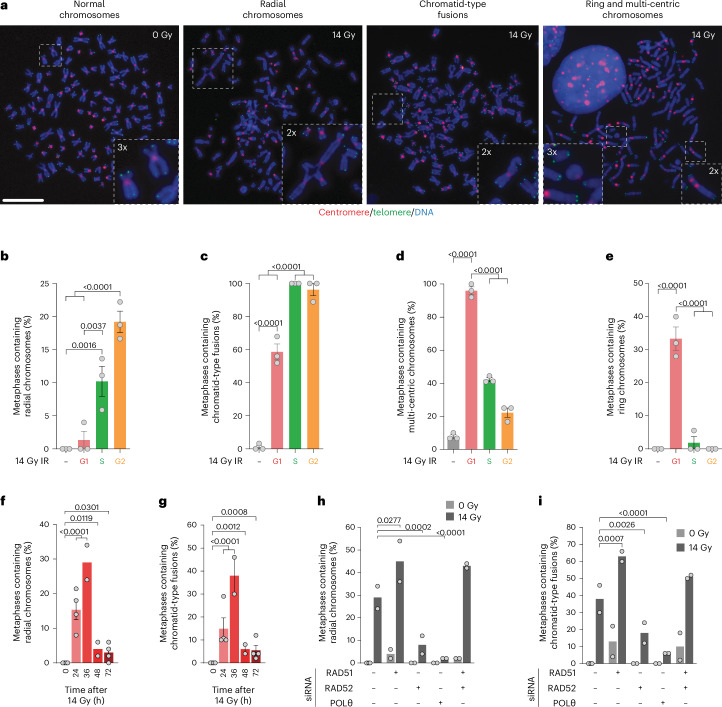
Chromosomal structural aberrations correlate with first mitosis survival. **a**, Cytogenetic preparations from HeLa ± 14 Gy. Scale bar, 10 µm (representative of *n* = 3). **b**–**e**, Radial chromosomes (**b**), chromatid-type fusions (**c**), multi-centric chromosomes (**d**) and ring chromosomes (**e**) from asynchronous mock irradiated or the first mitosis post 14 Gy IR in G1, S or G2-enriched HeLa (mean ± s.e.m. *n* = 3, *N* = 50 metaphases per replicate, ordinary one-way ANOVA with Fisher’s LSD multiple comparisons test). **f**,**g**, Radial chromosomes (**f**) and chromatid-type fusions (**g**) in asynchronous HeLa following 14 Gy IR (mean ± s.e.m. *n* = 4, 4, 2, 2 and 4 from left to right, *N* = 50 metaphases per replicate; two-sided Fisher’s exact test of *N*). **h**,**i**, HeLa radial chromosomes (**h**) and chromatid-type fusions (**i**) in the first mitosis post 14 Gy IR (mean *n* = 2, except 0 Gy control siRNA (*n* = 4), *N* = 50 metaphases per replicate, two-sided Fisher’s exact test of *N*). The control siRNA are the 0 and 36 h timepoints from **f** and **g**. Source data

Cytogenetic preparations from 14-Gy-irradiated HeLa revealed chromosome structural aberrations (Fig. 4a). We released G1/S synchronized 3F HeLa and irradiated 3, 6 or 14 h later corresponding with S, G2 or G1. The cytogenetic preparations from the immediate mitosis revealed preferential formation of radial chromosomes and chromatid-type fusions in S/G2-irradiated cells, and multi-centric chromosome-type fusions and circular chromosomes in G1-irradiated cells (Fig. 4b–e). Radials and chromatid-type fusions were present 24 and 36 hours post 14 Gy IR of asynchronous HeLa (Fig. 4f,g). RAD51 depletion, alone or with RAD52, rescued immediate mitotic death but increased mitoses containing radials or chromatid-type fusions, whereas singular RAD52 or POLθ depletion suppressed chromatid-type fusions and radials but increased mitotic lethality (Figs. 2f and 4h,i).

### Cohesion fatigue is associated with mitotic death

**Fig. 5:**
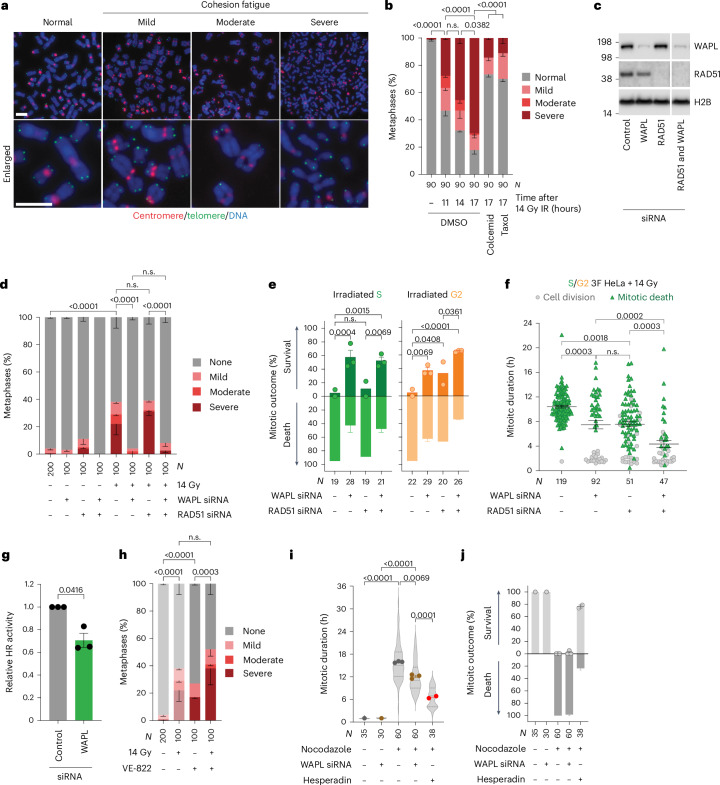
Cohesion fatigue promotes mitotic death following lethal genomic damage. **a**, Cohesion fatigue in HeLa chromosome spreads. Scale bars, 5 µm (representative of *n* = 5). **b**, Cohesion fatigue in HeLa ± 14 Gy IR, 100 ng ml^−1^ colcemid or 5 µg ml^−1^ taxol (mean ± s.e.m. *n* = 3, two-sided Fisher’s exact test of *N*). **c**, Western blots of HeLa whole-cell extracts (representative of *n* = 3). **d**, HeLa cohesion fatigue in the first mitosis after 14 Gy IR (mean ± s.e.m. *n* = 2, except 0 Gy control siRNA (*n* = 4), two-sided Fisher’s exact test of *N*). **e**,**f**, First mitosis outcome (**e**) and mitotic duration (**f**) in 14-Gy-irradiated asynchronous 3F HeLa (for **e**, mean ± s.e.m. *n* = 2, 3, 2 and 3 from left to right, two-sided Fisher’s exact test of *N*; for **f**, mean ± s.e.m. and Kruskal–Wallis uncorrected Dunn’s multiple comparisons test of *N*). **g**, HR of an I-SceI DSB repair reporter in HeLa (mean ± s.e.m. *n* = 3, two-tailed paired *t*-test, *t* = 4.8, d.f. of 2, 95% confidence interval 0.03 to 0.56). **h**, Cohesion fatigue in HeLa ± 14 Gy IR and/or 0.2 µM VE-822 (mean ± s.e.m. *n* = 2, except 0 Gy (*n* = 4), two-sided Fisher’s exact test of *N*). The samples without VE-822 are from Fig. 5d. **i**,**j**, Mitotic duration (**i**) and outcome (**j**) of HeLa ± siRNA, 0.5 µM hesperadin and/or 3.3 µM nocodazole (for **i**, points means ± s.e.m. of *n* = 1, 1, 3, 3 and 2 from left to right, violin plot of *N*, the dotted lines are quartiles and the solid lines medians, Kruskal–Wallis uncorrected Dunn’s multiple comparisons test; for **j**, mean ± s.e.m. as in **i**). The control siRNA + nocodazole and/or hesperadin are from Extended Data Fig. 4d. For **a**–**j**, *N* = individual cells/metaphases across all replicates and *n* = biological replicates. n.s., not significant. Source data

**Extended Data Fig. 6:**
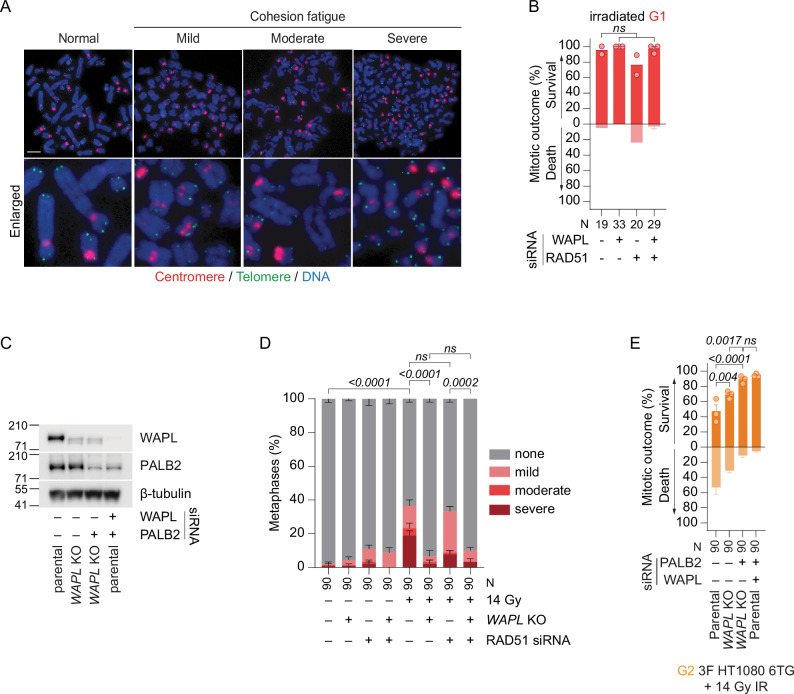
Targeting RAD51 or WAPL differently rescues immediate mitotic death. **A)** Cohesion fatigue in cytogenetic chromosome spreads from 14 Gy irradiated HT1080 6TG, scale bar = 5 µm (representative of n = 3). **B)** First mitosis outcome in 14 Gy irradiated G1 cells from asynchronous and siRNA transfected 3F HeLa (mean ± SEM, n = 2, 3, 2, 3 left to right, two-sided Fisher’s exact test of N). **C)** Western blots of whole cell extracts from parental and *WAPL* knockout (KO) 3F HT1080 6TG (representative n = 2). **D)** Cohesion fatigue in parental or *WAPL* KO HT1080 6TG cultures ± 14 Gy IR and/or siRNA (mean ± SEM of n = 3, two-sided Fisher’s exact test of N). **E)** First mitosis outcome in G2 enriched and 14 Gy irradiated parental or *WAPL* KO 3F HT1080 6TG ± siRNA (mean ± SEM, n = 3, two-sided Fisher’s exact test of N). All panels, N = individual cells across all replicates, n = biological replicates, ns = not significant. Source numerical data and unprocessed blots are available in source data. Source data

The cytogenetic preparations also revealed cohesion fatigue, a phenomenon characterized by unscheduled, asynchronous chromatid separation, and promoted by the cohesion antagonist WAPL^44^. Consistent with other forms of lethal genome damage^3^, cohesion fatigue following 14 Gy IR progressed from mild (separated sister centromeres), to moderate (separated centromeres and adjacent regions, cohesive telomeres), to severe (completely separated chromatids) as a function of mitotic duration and microtubule pulling forces negated by the poisons taxol or colcemid (Fig. 5a,b and Extended Data Fig. 6a).

Targeting WAPL suppressed cohesion fatigue and immediate mitotic death following 14 Gy IR (Fig. 5c–f and Extended Data Fig. 6b–e). WAPL siRNA did subtly reduce HR (Fig. 5g). However, unlike targeting WAPL, depleting RAD51 or inhibiting ATR failed to rescue cohesion fatigue following 14 Gy (Fig. 5d,h). Co-targeting WAPL and HR also provided additive mitotic survival in G2-irradiated cells (Fig. 5e and Extended Data Fig. 6e), and WAPL or RAD51 depletion differently affected the first mitosis after IR. WAPL depletion conferred bi-modal mitotic duration with survival strongly correlated with normal duration and death with arrest. These distinctions were blurred in RAD51-depleted cells (Fig. 5f). In nocodazole-treated 3F HeLa, WAPL siRNA failed to rescue mitotic death and reduced mitotic arrest to a significantly lesser degree then hesperadin, suggesting that targeting WAPL did not directly influence the SAC (Fig. 5i,j).

### Suppressing HR promotes chromosomal instability

**Fig. 6:**
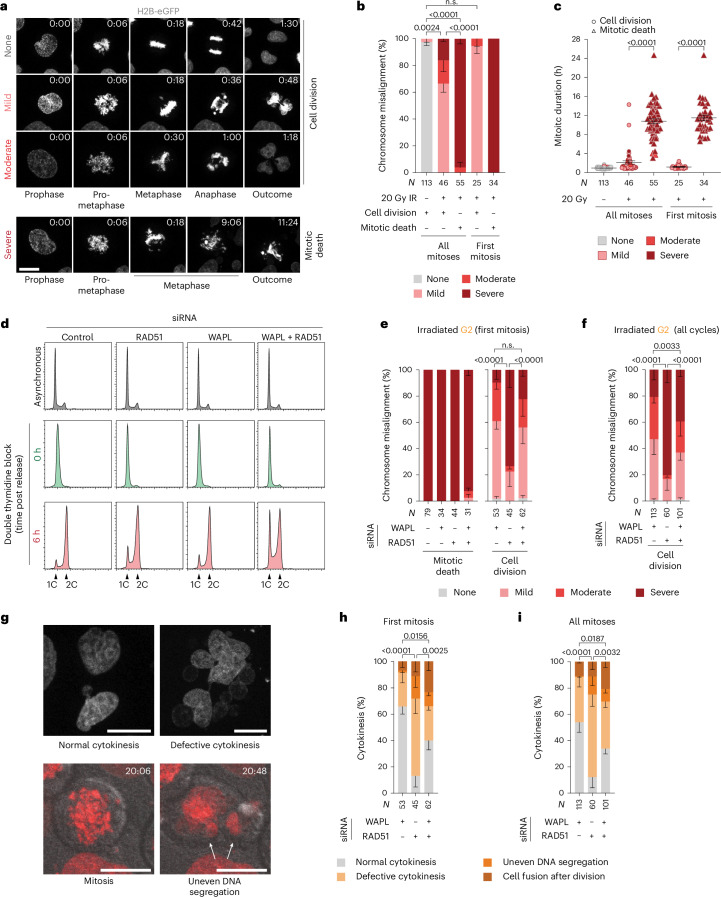
Targeting RAD51 promotes genomic instability upon mitotic death escape. **a**, Stills from live imaging of HeLa H2B–eGFP ± 20 Gy IR. Scale bar, 20 µm (representative of *n* = 2; hours:minutes is relative to prophase). **b**,**c**, Mitotic outcome (**b**) and duration (**c**) as a function of chromosome alignment in asynchronous HeLa H2B–eGFP ± 20 Gy IR (for **b**, mean ± s.e.m. *n* = 2, two-sided Fisher’s exact test of *N*; for **c**, mean ± s.e.m. and Kruskal–Wallis uncorrected Dunn’s multiple comparisons test of *N*). **d**, The cell cycle profiles of HeLa mCherry–H2B (representative of *n* = 2). C represents DNA content, where 1C is a haploid genome prior to DNA replication, while 2C stands for a diploid genome after DNA has been duplicated in S phase. **e**,**f**, Chromosome alignment in the first (**e**) and all (**f**) mitoses of G2-enriched HeLa mCherry–H2B treated with 14 Gy IR (mean ± s.e.m. *n* = 3, two-sided Fisher’s exact test of *N*). **g**, Representative images of cytokinesis defects. Scale bars, 20 µm (representative of *n* = 3). Arrows identify daughter cells with uneven DNA content. **h**,**i**, The cytokinesis outcomes from **e** and **f** in first (**h**) or subsequent (**i**) mitoses (mean ± s.e.m. *n* = 3, two-sided Fisher’s exact test of *N*). For **a**–**i**, *N* = individual cells across all replicates and *n* = biological replicates. n.s., not significant. Source data

We live-imaged asynchronous and 20-Gy-irradiated HeLa H2B–eGFP or mCherry–H2B and classified mitotic chromosome misalignment as mild (approximately one to three chromosomes outside the metaphase plate), moderate (greater than three chromosomes outside a structured metaphase and/or multi-polar spindle) or severe (the metaphase plate either did not form or spontaneously dispersed during mitosis) (Fig. 6a and Supplementary Video 5). A spontaneous loss of metaphase integrity is consistent with cohesion fatigue^3^. In asynchronous or G2-enriched HeLa, treated with 20 or 14 Gy, immediate mitotic arrest and death were associated with severe chromosome misalignment (Fig. 6b–e and Supplementary Videos 5 and 6). Relative to singular RAD51 depletion, WAPL siRNA alone or with RAD51 improved chromosome alignment and cytokinesis (Fig. 6e–i). WAPL depletion in irradiated cells, thus, countervails chromosome misalignment and cohesion fatigue to promote more effective cytokinesis. Conversely, RAD51 targeting enables mitotic exit despite cohesion fatigue and cytokinesis defects.

### Immediate mitotic death is executed via intrinsic apoptosis

**Fig. 7:**
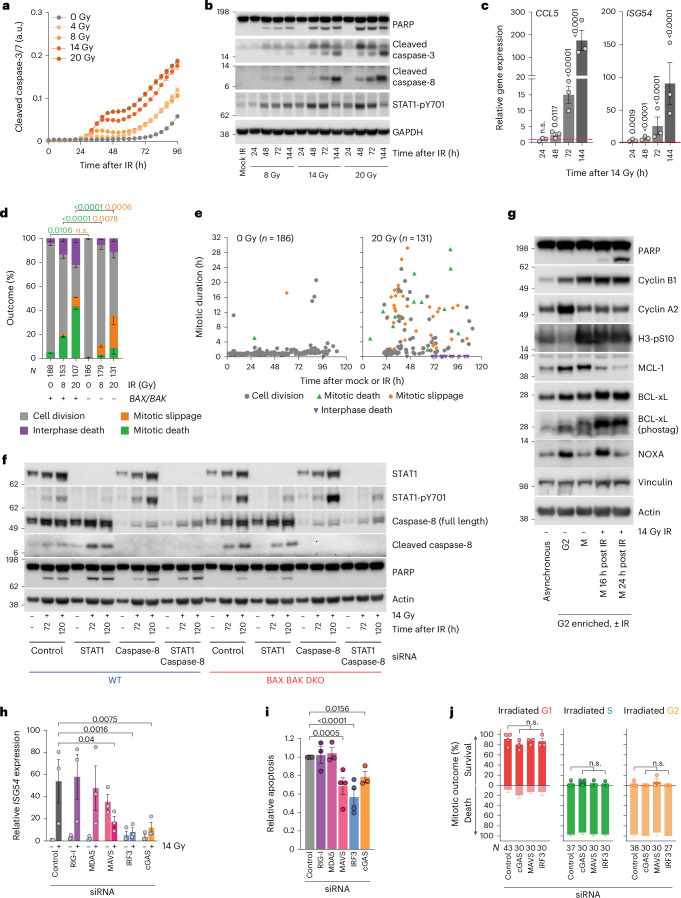
Distinct waves of intrinsic and extrinsic apoptosis following IR. **a**, IncuCyte live imaging of a caspase-3 and caspase-7 fluorescence reporter in HeLa (representative of *n* = 2, mean ± standard deviation of three technical replicates). a.u., arbitrary units. **b**, Western blots of HeLa whole-cell extracts (representative of *n* = 2). **c**, A quantitative PCR with reverse transcription (RT–qPCR) of HeLa post 14 Gy IR (mean ± s.e.m. *n* = 3, repeated measures (RM) one-way ANOVA with Fisher’s LSD multiple comparisons test). The fold change is relative to the cells collected immediately before IR and normalized to 1 (red dashed line). **d**, All cell cycle outcomes in HeLa ± IR and/or *BAX BAK* DKO (mean ± s.e.m. of *n* = 3, except 20 Gy (*n* = 2); two-sided Fisher’s exact test of *N*). **e**, Multi-dimensional representation of **d**. The symbols represent the individual outcomes. **f**, Western blots of whole-cell extracts from HeLa ± siRNA, *BAX* *BAK* DKO and/or 14 Gy IR (representative of *n* = 2). **g**, Western blots of whole-cell extracts from HeLa (representative of *n* = 2). The mitotic cells were collected by shakeoff. **h**, RT–qPCR of HeLa ± siRNA and/or 14 Gy IR, with a fold change to non-irradiated control siRNA normalized to 1 (mean ± s.e.m. *n* = 3, RM one-way ANOVA with Fisher’s LSD multiple comparisons test; the *P* values are relative to the control siRNA + 14 Gy). **i**, Relative apoptosis, as measured in **a**, in 14-Gy-irradiated HeLa (mean ± s.e.m. *n* = 4, 3, 3, 4, 4 and 3 from left to right, mixed-effect one-way ANOVA with a Fisher’s LSD multiple comparisons test). **j**, The first mitosis outcome in asynchronous and 14-Gy-irradiated 3F HeLa (mean ± s.e.m. *n* = 3, except control siRNA (*n* = 4), two-sided Fisher’s exact test of *N*). For **a**–**i**, *N* = individual cells across all replicates and *n* = biological replicates. n.s., not significant. Source data

**Extended Data Fig. 7:**
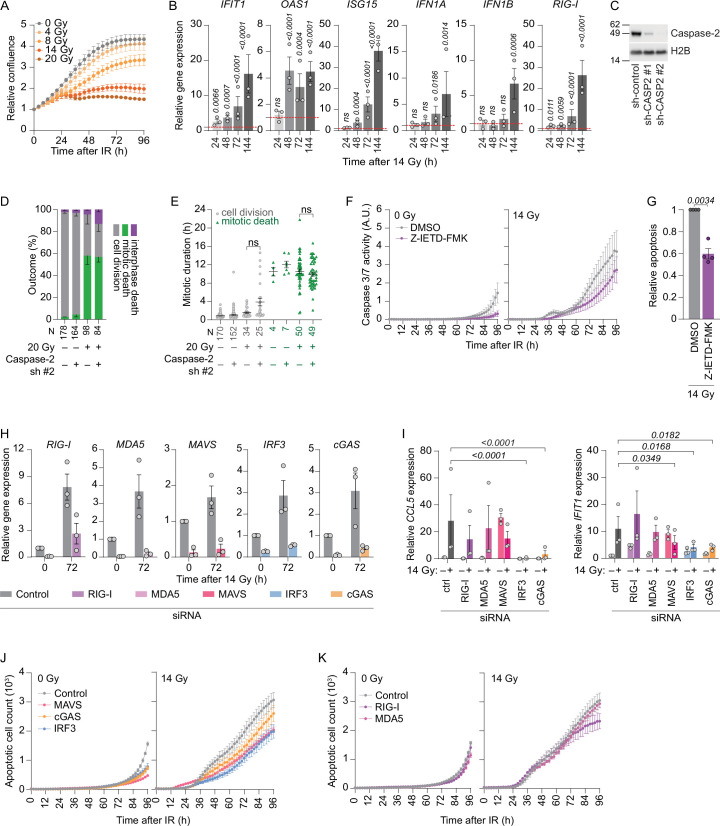
Distinct apoptotic pathways underpin immediate and delayed lethality following IR. **A)** HeLa proliferation measured through IncuCyte live imaging (mean ± SEM, n = 3). **B)** RT-qPCR from HeLa. Fold change presented relative to cells collected prior to IR (T0), normalized to 1 and depicted as a red dotted line (mean ± SEM, n = 3, RM one-way ANOVA with Fisher’s LSD multiple comparisons test, F and 95% CI in Source Data). **C)** Western blots of HeLa whole cell extracts. #1 are transduced with diluted 1:2 lentivectors and #2 with crude viral supernatant (n = 1). **D, E)** All cell cycle outcomes (**D**) and mitotic duration (**E**) in HeLa ± shRNA and/or 20 Gy IR (D, mean ± SEM n = 2, two-sided Fisher’s exact test of N; E, mean ± SEM and Kruskal-Wallis uncorrected Dunn’s multiple comparisons test of N). **F, G)** IncuCyte live imaging of a Caspase 3/7 fluorescence reporter (**F**) and relative apoptosis (**G**, area under the curve from F) in HeLa ± 14 Gy and/or 40 µM Z-IETD-FMK (mean ± SEM, n = 4; G, two-tailed paired t-test, t = 8.5, df = 3, 95% CI = -0.55 to -0.25). **H, I)** RT-qPCR of siRNA transfected HeLa. Fold change in gene expression relative to control siRNA immediately prior to IR (T0) and normalized to 1 (mean ± SEM n = 3; I, RM one-way ANOVA with Fisher’s LSD multiple comparisons test relative to control siRNA + 14 Gy IR, F and 95% CI in Source Data). **J, K)** IncuCyte live imaging of a Caspase 3/7 fluorescence reporter quantified in Fig. 7i (mean ± SEM; n in Fig. 7i legend). All panels, N = individual cells across all replicates, n = biological replicates, ns = not significant. Source numerical data and unprocessed blots are available in source data. Source data

Caspase-3 and caspase-7 function in intrinsic and extrinsic apoptosis, while caspase-8 functions specifically in extrinsic apoptosis^2,45,46^. Caspase-3 and caspase-7 activity was detected 32 h post IR with 8 Gy or more, with a second inflection from 56 h onward following ≥4 Gy (Fig. 7a and Extended Data Fig. 7a). Caspase-3 and PARP cleavage, known apoptotic hallmarks^47^, occurred contemporaneous with immediate mitotic and delayed lethality (compare Figs. 1f and 7b). Caspase-8 cleavage, however, was deferred and peaked with delayed lethality and ISG expression ≥72 h post IR (Fig. 7b,c and Extended Data Fig. 7b). *BAX* and *BAK* double KO (*BAX BAK* DKO) suppresses intrinsic apoptosis^3,48^ and significantly reduced mitotic death and PARP cleavage in irradiated HeLa (Fig. 7d–f). We observed no impact of caspase-2, a mitotic catastrophe associated factor^49^, in mitotic lethality following 20 Gy (Extended Data Fig. 7c–e). In other contexts, mitotic arrest initiates a competition between the inactivation of anti-apoptotic MCL1 and BCL-xL, which activates pro-apoptotic NOXA, and cyclin B1 degradation, which confers mitotic slippage^50,51^. We prepared whole-cell extracts from asynchronous, G2-enriched, or G2-enriched cultures allowed to enter mitosis and collected via shakeoff. Following 14 Gy IR in G2, we observed mitotic MCL1 degradation, BCL-xL phosphorylation and transient NOXA accumulation without cyclin B1 degradation (Fig. 7g), consistent with intrinsic apoptosis during mitotic arrest.

### Interferon production occurs with delayed lethality

The cells that completed the immediate mitosis after IR did so with chromosome structural aberrations and aberrant divisions (Figs. 4h,i and 6e–h), the outcomes associated with ISG expression^6,7,52^. Recognition of cytosolic DNA by cGAS or RNA by MAVS via RIG-I or MDA5 promotes IRF3-dependent STAT1 activation^53^. Phosphorylated STAT1-Y701 is a pro-inflammatory marker^54^. STAT1-pY701 preceded caspase-8 cleavage, ISG transcription and delayed lethality following IR (Fig. 7c,f and Extended Data Fig. 7b). Depleting caspase-8 suppressed PARP cleavage in 14-Gy-irradiated *BAX BAK* DKO cells (Fig. 7f), and inhibiting caspase-8 with Z-IETD-FMK reduced apoptosis at late timepoints after IR (Extended Data Fig. 7f,g).

Apoptosis is largely non-immunogenic^55,56^. Coincidently, STAT1-pY701 was enhanced when intrinsic and/or extrinsic apoptosis were suppressed by *BAX BAK* DKO and/or caspase-8 depletion (Fig. 7f). STAT1 depletion failed to suppress PARP or caspase-8 cleavage (Fig. 7f). Notwithstanding, depleting IRF3, cGAS or MAVS, but neither RIG-I nor MDA5 alone, suppressed ISG transcription and delayed apoptosis in 14-Gy-irradiated HeLa (Fig. 7h,i and Extended Data Fig. 7h–k). cGAS, MAVS or IRF3 siRNA did not affect the first cell cycle post IR (Fig. 7j). Cytoplasmic nucleic acid sensing and caspase-8 dependent-extrinsic apoptosis, thus, contribute to delayed lethality following IR, independent of STAT1.

### Affecting mitotic death modifies interferon expression

**Fig. 8:**
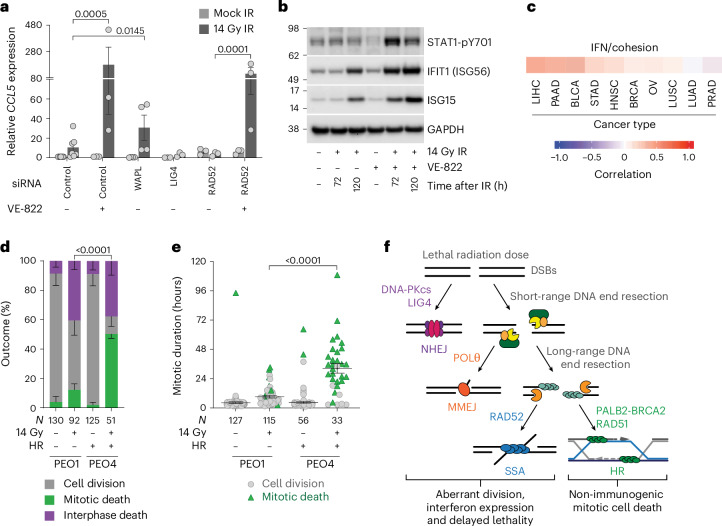
Mitotic death is tunable and influenced by disease genetics. **a**,**b**, Quantitative PCR with reverse transcription (**a**) and western blots of whole-cell extracts (**b**) from HeLa ± 14 Gy IR, siRNA and/or VE-822 (for **a**, mean ± s.e.m. *n* = 3, except the control—VE-822 (*n* = 6) and WAPL (*n* = 4); a mixed-effect one-way ANOVA with Fisher’s LSD multiple comparisons test was performed for the irradiated samples and fold change relative to non-irradiated control siRNA, normalized to 1; **b** is representative of *n* = 3). **c**, A Spearman correlation from mSigDB gene lists in TCGA patient samples. The tumour designations are in Methods. **d**,**e**, All cell cycle outcomes (**d**) and mitotic duration (**e**) in PEO1 and PEO4 ± 14 Gy IR (for **d**, mean ± s.e.m. *n* = 2, two-sided Fisher’s exact test of *N*; for **e**, mean ± s.e.m. and two-tailed Mann–Whitney test of *N*). **f**, A proposed decision tree with the corresponding outcome during mitotic catastrophe. For **a**–**f**, *N* = individual cells across all replicates and *n* = biological replicates. n.s., not significant. Source data

**Extended Data Fig. 8:**
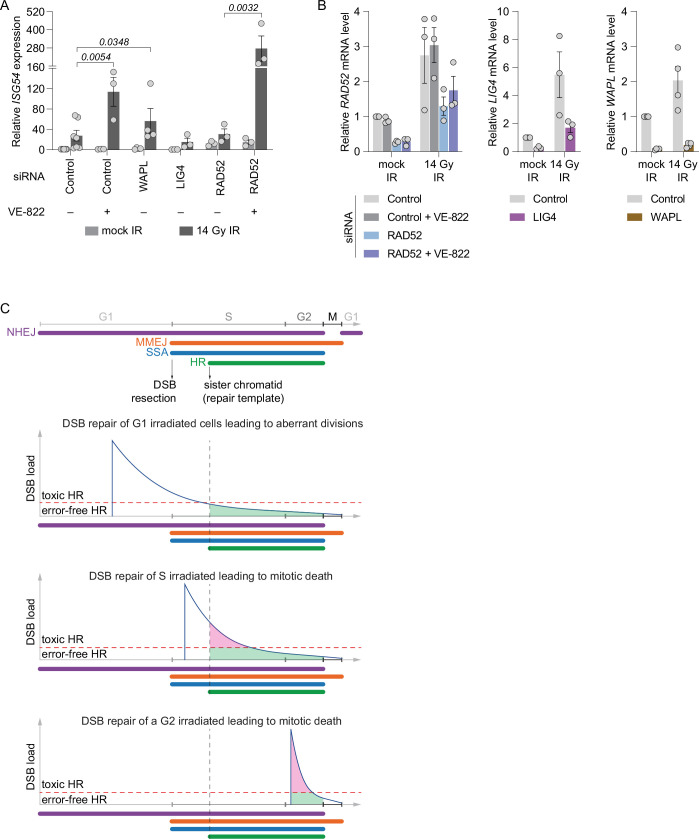
Pharmacological or genetic alteration of DSB repair impacts interferon expression following IR. **A)** RT-qPCR from HeLa ± 14 Gy IR, siRNA, and/or 0.2 µM VE-822 (mean ± SEM n = 3 except control n = 6, and WAPL siRNA n = 4, Mixed-effect one-way ANOVA with Fisher’s LSD multiple comparisons test, F and 95% CI in Source Data). **B)** RT-qPCR of siRNA in Fig. 8a and Extended Data Fig. 8A (mean ± SEM of n = 3). **C)** Model. Activity of DSB repair pathways across cell cycle (top). Interpretation of repair kinetics following DSB induction in G1, S, or G2 phase cells (below) as a function of DSB load and cell cycle progression. A red dotted line indicates a theoretical DSB threshold that separates functional (green shading) and toxic (magenta shading) HR. Source numerical data are available in source data. Source data

We postulate that mitotic death removed cells through an immunologically cold response, thus preventing subsequent interferon signalling. In agreement, VE-822 or WAPL siRNA rescued immediate mitotic death in 14-Gy-irradiated cultures and elevated STAT1-pY701, ISG transcript and/or interferon levels (Figs. 3f, 5e and 8a,b and Extended Data Fig. 8a,b). Conversely, LIG4 or RAD52 depletion enhanced immediate mitotic death after IR and did not increase ISG expression (Figs. 2f and 8a and Extended Data Figs. 2e and 8a,b). However, rescuing mitotic death in RAD52-depleted cells with VE-822 recovered elevated ISG transcripts, consistent with mitotic death suppressing interferon production (Figs. 3f and 8a and Extended Data Fig. 8a,b). Additionally, interrogating exome and RNA-sequencing data from The Cancer Genome Atlas (TCGA) revealed a positive correlation between cohesion and interferon gene signatures (Fig. 8c), reminiscent of enhanced cohesion following WAPL siRNA enabling mitotic survival and interferon production.

### *BRCA2* mutations suppress mitotic death

Finally, PEO1 and PEO4 are ovarian cancer lines derived from the same patient, which respectively carry inactivating and rescuing *BRCA2* mutations^57^. Consistent with HR-dependent mitotic lethality, HR-incompetent PEO1 and HR-competent PEO4 were respectively resistant and susceptible to immediate mitotic arrest and death following 14 Gy IR (Fig. 8d,e).

## Discussion

We identify a direct correlation in irradiated cultures between cell cycle-dependent DSB repair and cell death mode, with the distinction between mitotic death and survival in the first cell cycle determining the extent of interferon production. This clarifies the cause of disparate mitotic catastrophe outcomes and provides a roadmap for enhancing immunogenicity following clinical DSB induction.

Mitotic death following IR was potentiated through SAC-dependent mitotic arrest and canonical intrinsic apoptotic pathways. We propose that passage of HR intermediates into M resulted in persistent SAC activation. In support, immediate mitotic arrest and death were promoted by ATR, BRCA2, PALB2 and RAD51, up-stream factors that promote HR strand invasion^26^, and counteracted by RTEL1, BTR and SLX4, downstream factors that prevent or resolve dHJs^27–29^. Depleting GEN1, the mitotic HJ resolvase^31^, did not affect immediate mitotic death, suggesting that mitotic arrest originates from interphase HR intermediates passed into mitosis before GEN1 is active. How HR intermediates maintain SAC activation is unknown. Notably, our evidence suggests that neither HR factors nor WAPL directly regulate SAC signalling. We favour the hypothesis that chromosome entanglements created by dHJs between non-sister chromatids prevent SAC satisfaction. Such anomalous events should increase in an IR dose-dependent manner proportional with the DSB number. Targeting WAPL may also improve cohesion and reinforce HR between sister chromatids.

We propose that a repair hierarchy, coupled with DSB load, serves as a reliable predictor of mitotic catastrophe outcomes (Fig. 8f and Extended Data Fig. 8c). NHEJ ligates G1 DSBs at the potential cost of multi-centric or ring chromosomes. When p53 is absent, damaged G1 chromatin can pass into S (ref. ^58^), where MMEJ and SSA repair become active^59^, at the potential cost of radials or sister chromatid fusions. HR is available in S once the affected genome segment is replicated. Should NHEJ, MMEJ and SSA fail to rectify a break before replication in the adjacent region, HR may be engaged. Once the count or nature of breaks acted upon by HR result in the persistence of recombination intermediates into mitosis, mitotic arrest and death occur. Most G1-irradiated cells survived the first mitosis, but a dose-dependent minority of G1 damaged cells experienced immediate mitotic death. This aligns with an increasing probability of G1 damage evading end joining as DSBs escalate. Because HR is readily engaged in S or G2, IR in these phases results in mitotic death at lower dosages. HR and SSA compete^39,60^, and we observed premature RAD51 foci in G1/S of irradiated RAD52-depleted cultures. We expect that without RAD52, the breaks normally demarcated for SSA are RAD51 loaded, corresponding with enhanced mitotic death.

cGAS and MAVS are activated by aberrant cell divisions that may result from chromosomal aberrations following NHEJ, MMEJ or SSA repair^4,6–8^. cGAS and IRF3 were also implicated in mitotic death induced by mitotic poisons^61^. Our data suggest that within the IR context, immediate mitotic death is independent of cGAS, MAVS and IRF3. However, cGAS, IRF3, MAVS and caspase-8 all contributed to delayed extrinsic lethality following IR.

Targeting mitotic death exerted distinct effects on interferon production. Depleting LIG4 or RAD52 magnified mitotic death with no increase in ISG transcription, whereas ATR inhibition or WAPL siRNA suppressed mitotic death and amplified interferon production. We note that ATR inhibition conferred more ISG transcription than WAPL depletion and speculate this stems from improved cytokinesis outcomes and fewer cytosolic nucleic acids in WAPL-depleted cells. The patient data supported enhanced cohesion corresponding with suppressed interferon signalling, suggestive of clinical implications that warrant further exploration.

Highly precise stereotactic ablative radiotherapy (SABR)^62,63^, administered in one to five fractions of 8–21 Gy (ref. ^64^), is well situated to leverage mitotic catastrophe outcomes for patient benefit. Driving mitotic death in NHEJ, MMEJ or SSA mutant cancers, or combining SABR with inhibitors of these pathways, may widen the therapeutic index between normal and malignant tissues. Alternatively, compromised HR may promote a stronger immunogenic SABR response. In agreement, POLθ inhibitors induced immunogenicity specifically in BRCA2 mutant cells^65^. A personalized therapeutic approach that predicts cell death outcomes based on tumour genetics could yield therapeutic benefit, and promoting immunogenicity via HR inhibition has the potential to benefit patients worldwide.

## Methods

### Cell cultures

H2B–eGFP HeLa^66^, 2F IMR90 (ref. ^3^) and *BAX* *BAK* DKO HeLa from a parental CCL-2 strain^3^ were all created previously. We obtained the following cell lines from: HeLa (human, female; RRID: CVCL_0030), M. Chircop (CMRI); HT1080 6TG (human, male; RRID: not available)^67^, E. Stanbridge (University of California); IMR90 E6E7 (human, female; RRID: not available)^58^, J. Karlseder (Salk Institute); HCT116 p53 KO (human, male; RRID: CVCL_HD97), B. Vogelstein (Johns Hopkins University); T98G (human, male; RRID: CVCL_0556) and A549 (human, male; RRID: CVCL_0023), R. Reddel (CMRI); PEO1 (human, female; RRID: CVCL_2686) and PEO4 (human, female; RRID: CVCL_2690), A. DeFazio (WIMR); and Phoenix-AMPHO (human, female; RRID: CVCL_H716), ATCC. The 3F and 2F cell lines used in this study are subject to a material transfer agreement between the Riken BioResource Research Center, A.J.C. and CMRI. The cell line identity and purity was verified by Cell Bank Australia using short tandem repeat profiling, and all the cultures were routinely mycoplasma tested and found to be negative (MycoAlert, LT07-118, Lonza). All lines except PEO1, PEO4 and Phoenix-AMPHO were cultured at 37 °C, 10% CO_2_ and 3% O_2_ in DMEM (Life Technologies) supplemented with 1% non-essential amino acids (Life Technologies) and 1% Glutamax (Life Technologies). HeLa, HT1080 6TG, HCT116 p53 KO and their derivatives were supplemented with 10% bovine growth serum (Hyclone). T98G, A549, IMR90 and their derivatives were supplemented with 10% foetal bovine serum (Life Technologies). PEO1 and PEO4 cells were cultured at 37 °C, 10% CO_2_ and 3% O_2_ in RPMI (Life Technologies) supplemented with 20 mM HEPES buffer (Sigma-Aldrich, H0887), 1% Glutamax and 10% foetal bovine serum. The Phoenix-AMPHO cells were cultured at 37 °C, 10% CO_2_ and 3% O_2_ in DMEM (Life Technologies) supplemented with 1% non-essential amino acids (Life Technologies) and 10% foetal bovine serum (Life Technologies).

### Cell treatments

The cells were respectively irradiated as a monolayer or in suspension using an X-RAD 320 (1 Gy min^−1^; Precision X-Ray) or GammaCell 3000 Elan (1.59 Gy min^−1^ ± 0.07; Best Theratronics) irradiator. The cells were treated with the following compounds: DMSO (Sigma-Aldrich, D8418), 0.5 µM reversine added 2 h before mock or IR treatment (Selleckchem, S7588), 40 µM Z-IETD-FMK (Selleckchem, S7314) added 30 min before mock or IR, 0.5 µM NU7441 added 30 min before mock or IR (Selleckchem, S2638), 50 µM RI(dl)-2 (MedChemExpress, HY-126972A), 10 µM 5-ethynyl-2′-deoxyuridine (EdU; Sigma-Aldrich, 900584), 10 µM RO3306 (Sigma-Aldrich, SML0569), 0.2 µM VE-822 added 1 h before mock or IR treatment (Selleckchem, S7102), 5 µg ml^−1^ taxol (Sigma-Aldrich, T7191), 100 ng ml^−1^ or 250 nM colcemid (Gibco ThermoFisher, 15212012), 3.3 µM nocodazole (Sigma-Aldrich, M1404), 0.5 µM hesperadin (Selleckchem, S1529) or 2 mM thymidine (Sigma-Aldrich, T1895). In Fig. 5b, colcemid or taxol were added 11 h post IR.

### Viral transduction and cell line generation

The 3F cells were created using tFucci(CA)2/pCSII-EF (RDB15446)^32^ provided by H. Miyoshi (Keio University) through RIKEN BRC. We used previously created caspase-2 (Sigma-Aldrich, TRCN0000003508; targeted sequence GTTGAGCTGTGACTACGACTT) and control short hairpin RNAs (shRNAs) (Addgene plasmid no. 1864; http://n2t.net/addgene:1864; RRID: Addgene_1864, a gift from D. Sabatini). The third generation lentivectors harbouring tFucci(CA)2/pCSII-EF, control or caspase-2 shRNAs were produced in the CMRI Vector and Genome Engineering Facility. The cultures were transduced for 24 (scrambled and caspase-2 shRNA) or 48 (3F HeLa) h with crude supernatants (scrambled and caspase-2 shRNA), viral supernatant diluted 1:1 in fresh media (caspase-2 shRNA) or purified lentivector particles at a multiplicity of infection of 3 (3F HeLa) in media supplemented with 4 µg ml^−1^ of polybrene. The scrambled and caspase-2 shRNA transduced HeLa cultures were selected with 1 µg ml^−1^ puromycin (Sigma-Aldrich, P8833) for 3 days. The 3F HeLa cells were cultured for 6 days and sorted at the Westmead Institute for Medical Research Flow Cytometry Facility for mCherry expression. The 3F cultures were expanded for 6 days in 1 µg ml^−1^ of normocin (Invivogen, ant-nr-1) supplemented media and appropriate cell cycle-dependent colorimetric signalling verified by live imaging.

pLXSN H2B–mCherry was provided by L. Crabbe^68^. We moved mCherry from the C terminus to the N terminus of H2B through polymerase chain reaction (PCR) amplification of the H2B, mCherry and pLSXN backbone, followed by infusion cloning (Takara, 638911). The primers are listed in Supplementary Table 1. The retroviral vectors were created by transfecting Phoenix-AMPHO cells with pLXSN mCherry–H2B using Lipofectamine 3000 (Invitrogen Thermo Fisher Scientific, L3000015) according to the manufacturer’s instructions. The viral supernatant was used to infect HeLa cells, and stable mCherry-H2B cultures were selected with 600 µg ml^−1^ G418 (Sigma-Aldrich, 4727878001). The fluorescent mCherry signal was confirmed by live imaging.

### CRISPR-Cas9 knockout cell line generation

*WAPL*-KO HT1080 6TG 3F were generated by reverse transfection with SpCas9 2NLS and the multi-guide Gene-Knockout Kit v2 targeting human *WAPL* (guide RNAs no. 1 5′-UCAUCACUAUCAAAUCCAAA, no. 2 5′-AUAAGAGGAACACUUAACCU and no. 3 5′-UUCGGAAUUUCUUGGAUAUC) according to the manufacturer’s protocol (Synthego). The KO pool was screened by Sanger sequencing (forward: 5′-AAGTGTCCTCCAAAGGGAAG; reverse: 3′-CACAGAGAATATGAAACTGGTGTCA), followed by a western blot confirmation. The *BAX BAK*-KO HeLa cells were generated previously^3^.

### Live imaging

All imaging was performed in the CMRI Telomere Analysis Center (ATAC). Live imaging was done at 37 °C, 10% CO_2_ and 3% O_2_ with imaging commencing as soon as possible after mock or IR. Events within the first ~60 min following IR were not recorded due to sample transport and imaging setup. The images were captured in 6 min intervals for the experiment duration. Differential interference contrast (DIC) with or without epifluorescence was acquired on a ZEISS Cell Observer inverted wide field microscope fitted with a 20× 0.8 numerical aperture (NA) air objective and Axiocam 506 monochromatic camera (ZEISS) using ZEN Blue v2.6 software (ZEISS). A ZEISS HXP 120C mercury short-arc lamp and compatible filter cubes were used to generate fluorescent images. The chromosome dynamics in mCherry–H2B were captured using a ZEISS Cell Observer SD spinning disc confocal microscope fitted with 40× 1.3 NA air objective, a 561 nm 50 mW solid state excitation laser (30% excitation power, 1× 1 binning, Electron Multiplication gain of 300) with appropriate filters and an Evolve Delta camera (Photometrics) using ZEN Blue v2 software (ZEISS). H2B–eGFP HeLa cultures were imaged on a Cell Voyager confocal microscope equipped with a 40× 0.95 NA objective (Olympus), 488 nm laser (10–20% excitation power, 1× 1 binning, 65% gain) with compatible filter cubes and a high sensitivity EMCCD camera using CV1000 software (Yokogawa). A total of 11–13 *Z* stacks were taken at 1 µm increments, and the outcomes were scored manually. For DIC, the outcomes were assigned on the basis of morphological features. The mitotic duration was calculated from the frame preceding nuclear envelope breakdown until cytokinesis completion, typically two or three frames after anaphase onset. For 3F and 2F cultures, the cell cycle was defined by a FUCCI colouration^32,35^. For mCherry–H2B and H2B–eGFP HeLa cultures, chromosome misalignments were scored by eye. All live cell imaging analyses were performed using ZEN Blue v2 or v2.6 or CV1000 software.

### Western blotting

The whole-cell extract preparation and western blots were performed as described previously^69^. Detection of BCL-xL on phostag gels (FujiFilm, catalogue number 192-18001) was done according to manufacturer’s protocol. Luminescence signals were visualized on an LAS 4000 imager (Fujifilm). Primary antibodies include: β-actin (AC-15, Sigma-Aldrich, A5441, 1:10,000), β-tubulin (Abcam, ab6046; 1:500), BCL-xL (54H6, Cell Signaling Technology, 2764, 1:1,000), BRCA2 (Ab-1, clone 2B, Sigma-Aldrich, OP95, 1:1,000), caspase-2 (C2, Cell Signalling Technology, 2224, 1:1,000), caspase-8 (D35G2, Cell Signalling Technology, 4790, 1:1,000), CHK1 (2G1D5, Cell Signaling Technology, 2360, 1:1,000), CHK1-pS345 (133D3, Cell Signaling Technology, 2348, 1:1,000), cleaved caspase-3 (Asp175, Cell Signaling Technology, 9661, 1:500), cleaved caspase-8 (Asp384, 11G10, Cell Signaling Technology, 9748, 1:1,000), cyclin A2 (EPR17351, Abcam, ab181591, 1:3,000), cyclin B1 (D5C10, Cell Signaling Technology, 12231, 1:2,000), DNA-PKcs (Y393, Abcam, ab32566, 1:1,000), GAPDH (D16H11, Cell Signaling Technology, 5174, 1:5,000), histone H2B (V119, Cell Signaling Technology, 8135, 1:1,000), histone H3-pS10 (D2C8, Cell Signaling Technology, 2224, 1:1,000), IFIT1 (D2X9Z, Cell Signaling Technology, 14769, 1:1,000), ISG15 (Cell Signaling Technology, 2743, 1:1,000), LIG4 (EPR16531, Abcam, ab193353, 1:1,000), MCL1 (Y37, Abcam, ab32087, 1:1,000), NOXA (D8L7U, Cell Signaling Technology, 14766, 1:1,000), PALB2 (Bethyl, A301-246A, 1:1,000), PARP (46D11, Signaling Technology, 9532, 1:1,000), POLθ (Thermo Fisher Scientific, PA5-69577, 1:250), STAT1 (Cell Signaling Technology, 9172, 1:1,000), STAT1-pY701 (58D6, Cell Signaling Technology, 9167, 1:1,000), RAD51 (14B4. Novus, NB100-148, 1:500), RAD52 (F-7, Santa Cruz, sc-365341, 1:500), RMI2 (Thermo Fisher Scientific, PA5-95632, 1:1,000), RTEL1 (Novus, NBP2-22360, 1:1,000), vinculin (hVIN-1, Sigma-Aldrich, V9131, 1:10,000) and WAPL (A-7, Santa Cruz, sc-365189, 1:1,000). The secondary antibodies include horseradish peroxidase conjugated goat anti-mouse (Dako, P0447, 1:5,000–1:20,000) and goat anti-rabbit (Dako, P0448, 1:5,000–1:20,000).

### Quantitative PCR with reverse transcription

The total RNA was isolated from cell pellets collected by trypsinization using the RNeasy Mini Kit (Qiagen, 74104) according to the manufacturer’s instructions. The genomic DNA was eliminated by on-column digestion for 30 min at 37 °C with DNase I (Thermo Fisher Scientific, EN0521). A total of 500 ng of total RNA as determined by NanoDrop spectrophotometer (Thermo Fisher Scientific, ND-1000) was reverse transcribed using the SensiFast cDNA Synthesis Kit (Meridian Bioscience, BIO-65054), according to the manufacturer’s instructions, and diluted five times. A quantitative PCR was performed using 2 µl of diluted cDNA, 200 nM gene-specific primers designed to span exon–exon junctions whenever possible (Supplementary Table 2 and ref. ^70^) and PowerUp SYBR Green Master Mix (Thermo Fisher Scientific, A25742). A total volume of 10 µl per reaction was run on a QuantStudio 6 Flex Real-Time PCR System (Applied Biosystems Thermo Fisher Scientific, 4485691) for 45 cycles (2 min at 50 °C and 2 min at 95 °C, then cycling for 1 s at 95 °C and 30 s at 60 °C). The data were acquired using QuantStudio Real-Time PCR Software v1.7.2 (Applied Biosystems and Thermo Fisher Scientific). The relative gene expression was normalized to GAPDH, and the fold change was calculated as 2^−ΔΔCt^ in Microsoft Excel v2308. The statistical analysis was performed using ΔΔCt values.

### siRNA transfection

A transient gene knockdown was achieved using individual or pooled siRNA (Supplementary Table 3) at the final concentration of 10 nM. A reverse siRNA transfection was performed 72 h before irradiation using Lipofectamine RNAiMAX (Invitrogen Thermo Fisher Scientific, 13778150) according to the manufacturer’s instructions.

### CRISPR-mediated repair assay

We measured NHEJ and MMEJ repair of a genomic loci through Tracking Indels by Decomposition (TIDE) using a clustered regularly interspaced short palindromic repeats (CRISPR) guide RNA (gRNA) targeting a single genomic location (LBR2) known to be repaired by NHEJ and MMEJ with different kinetics^71^. The LBR2 Alt-R gRNA was generated by Integrated DNA Technologies (GCCGATGGTGAAGTGGTAAG). The TracrRNA:gRNA duplex was transfected according to manufacturer’s protocol. Briefly, tracrRNA and gRNA were hybridized at 95 °C for 5 min in a 1:1 ratio. After hybridization, the mixture was combined with RNAiMAX diluted in Opti-MEM (Gibco Thermo Fisher Scientific, 11058021) and incubated at room temperature for 20 min. A total of 72 h post transfection, the cells were lysed with Direct-PCR lysis (Viagen, catalogue number 301-C) and incubated overnight with 0.2 mg ml^−1^ of Proteinase K (Roche, 03115801001) at 55 °C. A PCR spanning the LBR2 CRISPR site was performed using MyTaq Red Mix (Meridian, BIO-25043), and the PCR products were subjected to Sanger Sequencing. The editing efficiency and indel pattern of the LBR2 gRNA upon DNA-PKcs inhibition and POLθ depletion were measured using TIDE software^72^. The MMEJ and NHEJ ratios were calculated using the following formulas: frequency −7 deletion/((frequency +1 insertion) + (frequency −7 deletion)) and frequency +1 insertion/((frequency +1 insertion) + (frequency −7 deletion)), respectively.

### DSB repair reporter cell lines

The DSB repair reporter vectors pDRGFP (Addgene plasmid no. 26475; http://n2t.net/addgene:26475; RRID: Addgene_26475) and hprtSAGFP (Addgene plasmid no. 41594; http://n2t.net/addgene:41594; RRID: Addgene_41594) were provided by M. Jasin. The DSB reporter cell lines were generated by transfection of HeLa cells with reporter constructs using Lipofectamine 3000 (Invitrogen Thermo Fisher Scientific, L3000015) according to the manufacturer’s instructions. The stable integration of reporter constructs was confirmed by selection with puromycin (1 µg ml^−1^), which was added 48 h after transfection and maintained in cell media for 2 weeks.

To measure DSB repair, the reporter cell lines were transfected with pCBASce (Addgene plasmid no. 26477; http://n2t.net/addgene:26477; RRID: Addgene_26477, a gift from M. Jasin) plasmid using Lipofectamine 3000 according to the manufacturer’s instructions, resulting in production of the endonuclease I-SceI, which cleaves the stably integrated reporter constructs. The subsequent green fluorescent protein (GFP) expression acts as a measure of DSB repair^17,73^. The reporter cells were transfected in parallel with pCAGGS–mCherry (provided by P. Sharp; Addgene plasmid no. 41583; http://n2t.net/addgene:41583; RRID: Addgene_41583) to estimate transfection efficiency and calculate relative DSB repair activity. A total of 48 h post transfection, the cells were collected and GFP and mCherry expression quantified on FACSCantoII (BD Biosciences) using FACSDiva 6.1.3 software and analysed using FlowJo v10.8.0. The relative DSB repair capacity was calculated as a ratio of the GFP-positive cells (per cent, %) to mCherry-positive cells (per cent, %) and normalized to a control condition (equal 1). The gating strategies are shown in Supplementary Fig. 1.

### RAD51 foci labelling

A total of 72 h after siRNA transfection, the cells grown on sterile glass coverslips were treated with DMSO or 0.2 µM VE-822 and simultaneously pulse-labelled with 10 µM EdU for 1 h. The cells were fixed immediately (4% paraformaldehyde in PBS for 10 min at 4 °C) or irradiated and maintained for 2 h with 10 µM RO-3306 and DMSO or 0.2 µM VE-822 before fixation. All the wash steps were performed in PBS with 0.1% Tween. The samples were washed three times, permeabilized with 0.5% Triton X-100 in PBS + 0.1% Tween (PBST) for 5 min, washed three times again and blocked with 2% BSA buffer (in PBST) for 2 h. The samples were incubated overnight at 4 °C with primary anti-RAD51 antibody (Calbiochem, PC130, 1:500 in 2% BSA), then washed five times. All subsequent steps were performed in the dark. The coverslips were incubated with a click-iT EdU solution of 10 μM 6-carboxyfluorescein-TEG azide (Berry and Associates, FF 6110), 10 mM sodium ʟ-ascorbate (Sigma-Aldrich, A4034), 2 mM copper (II) sulphate (Sigma-Aldrich, 451657 in PBS) for 30 min. The cells were washed five times in 1% BSA (in PBST) before staining with Alexa Fluor 568-conjugated secondary antibody (Invitrogen, A11036, 1:500 in 2% BSA) for 1.5 h. The cells were washed three times, incubated with 4,6-diamidino-2-phenylindole (DAPI; Sigma-Aldrich, 10236276001) for 5 min (1:5000 in PBST), washed 1×, rinsed twice with milliQ H_2_O and sequentially dehydrated in 70%, 90% and 100% ethanol for 3 min each. The air-dried coverslips were mounted with Prolong Gold Antifade (Invitrogen, Thermo Fisher Scientific, P36934) and cured in the dark for ≥24 h. The fixed images were acquired with a ZEISS AxioImager Z.2 microscope fitted with a 40× 1.3 NA oil objective, Axiocam 506 monochromatic camera using ZEN Blue v2.3 pro software. A total of 19 *Z* stacks for each channel were taken at 0.24 µm increments.

### Image analysis of RAD51 foci

The extended depth of focus function in ZEN Blue v2.3 pro software was used to make maximum intensity projections of each image, then the .CZI files were converted to single-channel .TIFF files and imported into CellProfiler v4.2.1. Using custom image analysis pipelines, we pre-processed images using the CorrectIllumination and EnhanceOrSuppress feature functions. The nuclei and subnuclear foci were identified using the IdentifyPrimaryObjects functions with either Minimum Cross-Entropy or Otsu thresholding. The foci were associated with their respective nuclei using the Relate function, and the foci intensities were measured using the MeasureObjectIntensity function. The data were plotted in GraphPad Prism v9.3.1 using a double-gradient colourmap for the multiple variables plots.

### Cytogenetic chromosome spreads and fluorescent hybridization in situ

The cytogenetic chromosome spreads were performed as described^74^. The cells were arrested in mitosis with 250 nM colcemid (Gibco Thermo Fisher Scientific, 15212012) for 1 h, fixed with methanol and acetic acid (3:1), dropped onto glass slides, fixed with 2% paraformaldehyde and dehydrated in a graded ethanol series (70%, 90% and 100%). The slides were denatured at 80 °C for 10 min and hybridized in the dark overnight with peptide nucleic acid (PNA) probes against telomeric (Alexa488-OO-ccctaaccctaaccctaa; Panagene, F1004) and centromeric (Alexa647-OO-aaactagacagaagcatt-KK; Panagene, F3015) sequences. All the subsequent steps were performed in the dark. The slides were washed with PNA wash A (70% formamide and 10 mM Tris pH 7.5), followed by PNA wash B (50 mM Tris pH 7.5, 150 mM NaCl and 0.8% Tween 20), with DAPI added in the latter wash as a counterstain. The slides were mounted using Prolong Gold overnight before imaging. The metaphase searching and image acquisition was performed as described^58^ using Metafer4 v3.12.8 software combined with automated MetaSystems imaging platform on a ZEISS AxioImager Z.2 microscope fitted with a 63× 1.4 NA oil objective, appropriate filter cubes and a CoolCube1 camera (MetaSystems). For Fig. 5d and Extended Data Fig. 6d, the samples were collected 36 h post IR for control and WAPL-knockdown cells and 24 h post IR for RAD51 and RAD51/WAPL siRNA. Different timepoints were required because RAD51 depletion accelerated mitotic entry of S/G2-irradiated cells.

### Image analysis of cytogenetic chromosome spreads

Metaphase images were analysed using ISIS v5.8.8 software (MetaSystems) by eye. Metaphases containing a single chromatid-type fusion or radial chromosome were scored as positive for those events. The cohesion status was considered normal if less than three chromosomes exhibited aberrant cohesion, mild if greater than or equal to three chromosomes exhibited centromeric cohesion loss but retained cohesion between chromosomal arms, moderate if greater than or equal to three chromosomes exhibited centromeric cohesion loss together with separation of adjacent chromosomal regions, and severe if greater than or equal to three chromosomes exhibited complete loss of chromatid cohesion^3^. In practice, the cohesion status was typically consistent across all chromosomes.

### Cell cycle synchronization

The cells were synchronized at G1/S using a double thymidine block by treating with 2 mM thymidine for 16–18 h, releasing for 6–8 h and treating again with 2 mM thymidine for 16–18 h. The cell cycle phase enrichment was determined by flow cytometry or FUCCI colouration.

### Cell cycle analysis by flow cytometry

The cells were fixed in ice-cold 70% ethanol. Where required, the samples were stored overnight at −20 °C. The cells were pelleted, washed in PBS and resuspended in solution containing propidium iodide (1 mg ml^−1^) and RNase A (0.5 mg ml^−1^; Qiagen, 1007885). The cell cycle distribution was quantified on FACSCantoII (BD Biosciences) using FACSDiva 6.1.3 software and analysed using FlowJo v10.8.0. The gating strategies are shown in Supplementary Fig. 1.

### IncuCyte proliferation and apoptosis quantitation

The cultures were imaged using an IncuCyte live imaging system (Sartorius) at 37 °C, 10% CO_2_ and 3% O_2_, with the images captured at 10× magnification every 2 or 4 h for the experiment duration. The proliferation was calculated on the basis of a confluence mask generated with the IncuCyte Zoom software (Sartorius, version 2019B). We detected apoptosis using the NucView488 reagent (Biotium, 10402), which is a caspase-3 and caspase-7 enzyme substrate that emits green fluorescence upon apoptotic cleavage. A ratio of 1:1,000 NucView488 ± DMSO or 40 µM Z-IETD-FMK were added into FluoroBrite (Gibco ThermoFisher, A1896701) media ≥30 min before mock or IR. The caspase-3 and caspase-7 activity was calculated on the basis of generated cell mask and expressed as a total integrated intensity (for pharmacological treatments) or apoptotic cell count under conditions that affected cell size and/or morphology (genetic knockdowns). Apoptosis, as a temporal function of caspase-3 and caspase-7 activity, was calculated as an area under the curve relative to DMSO-treated cultures and normalized to 1 using GraphPad Prism v9.3.1.

### TCGA analysis

Gene expression and somatic mutation data were obtained for samples across cancer types from TCGA accessed through the Broad Institute portal Firebrowse (http://firebrowse.org/). RNA-Seq by Expectation-Maximization normalized, log_2_(*x* + 1)-transformed gene expression values were used for gene signature evaluation (signatures were taken as lists of genes from the Broad Institute database MSigDB), and the median of signature genes’ expression was taken as the signature score for each patient sample. Spearman correlations were computed using the ppcor package v1.1 in R v3.5 (https://cran.r-project.org/) between pairs of signature scores across TP53 mutated samples (non-synonymous, non-intronic and not in an intergenic region) for a given cancer type. The correlations were only calculated if both groups had at least ten non-NA values and only if at least ten samples were present with paired mutation and gene expression information for the cancer type. The cancer subtypes are: LIHC, liver hepatocellular carcinoma; PAAD, pancreatic adenocarcinoma; BLCA, bladder cancer; STAD, stomach adenocarcinoma; HNSC, head and neck squamous cell carcinoma; BRCA, breast cancer; OV, ovarian cancer; LUSC, lung squamous cell carcinoma; LUAD, lung adenocarcinoma; and PRAD, prostate adenocarcinoma.

### Statistics and reproducibility

All statistical analyses were performed using GraphPad Prism v9.3.1. Statistical methods and the number of cells (*N*), biological replicates (*n*) and error bars are described in the figure legends. The exact *P* values are provided for all statistically significant comparisons greater than *P* = 0.0001. No statistical method was applied to predetermine sample size. The sample sizes are consistent with published studies using similar experimentation^3,75,76^ and are reported in the figure legends. No data were excluded from the analyses. The experiments were performed with biologically consistent outcomes across eight cell lines in multiple independent biological replicates as described in figure legends. The violin plots represent a cumulative distribution of data points from all replicates per condition. Where *n* = 2 ± standard error of the mean (s.e.m.), the error bar caps are equal to the range of the replicate means. The representative data, whenever shown, are characteristic of similar results from the indicated number of independent biological replicates as described in figure legends. The samples were randomly allocated into experimental groups. The data analysis was randomized as follows. In live imaging experiments, the cells within the field of view were selected randomly before analysis and followed for the experiential duration. For cytogenetic analysis, chromosome spreads were chosen at random and captured through automated imaging. For quantitation of RAD51 foci, all interphase cells completely contained within the field views were analysed using automated methods. All experiments were performed a minimum of twice, unless specified otherwise, with quantification and statistics derived from *n* biological replicates or *N* cells, as described in the figure legends. The Fisher’s exact test was applied to categorical data. For continuous data, parametric (*t*-test, analysis of variance (ANOVA)) and non-parametric (Kruskal–Wallis, Mann–Whitney and Kolmogorov–Smirnov) tests were applied as a function of normal and non-normal data distribution assumptions that were not formally tested. The researchers were not blinded due to impracticability. The figures were prepared using Adobe Photoshop v25.0 and Illustrator v27.9.

### Reporting summary

Further information on research design is available in the Nature Portfolio Reporting Summary linked to this article.

## Online content

Any methods, additional references, Nature Portfolio reporting summaries, source data, extended data, supplementary information, acknowledgements, peer review information; details of author contributions and competing interests; and statements of data and code availability are available at 10.1038/s41556-024-01557-x.

## Supplementary information

Supplementary Information Supplementary Fig. 1

Reporting Summary

Peer Review File

Supplementary Video 1 Live imaging of 3F HeLa. Combined DIC and fluorescence live imaging of 3F HeLa cells treated with mock or 14 Gy IR. Cell cycle colouration as shown in Fig. 1a. Time is relative to imaging commencement within one hour of mock or IR treatment (representative of *n* = 3).

Supplementary Video 2 Cell outcomes following IR. Examples of cellular outcomes observed with combined DIC and fluorescence live imaging in 3F HeLa cultures treated with mock or 14 Gy IR. Time is hours:minutes relative to the start of the first image in the depicted outcome. Scale bars, 20 µm (representative of *n* = 3).

Supplementary Video 3 DNA-PKcs inhibition promotes mitotic death in G1-irradiated cells. DIC and fluorescence live imaging of 3F HeLa cells treated with 20 Gy IR ± 0.5 µM NU7441. Time is relative to imaging commencement within 1 hour of mock or IR treatment. NU7441 was added 30 min before irradiation (representative of *n* = 2).

Supplementary Video 4 RAD51 depletion rescues immediate mitotic death of G2-irradiated cells. DIC and fluorescence live imaging of G2-enriched 3F HeLa cells treated with control or RAD51 siRNA and 14 Gy IR. The cells were synchronized at G1/S boundary by double thymidine block, released for 6 h and irradiated in G2 phase as confirmed by 3F colouration. Time is relative to imaging commencement within 1 h of mock or IR treatment (representative of *n* = 3).

Supplementary Video 5 Chromosome segregation errors in cells that complete the first mitosis after genomic damage. Spinning disc confocal live imaging of H2B–eGFP HeLa cultures treated with mock or 20 Gy IR. Time is shown as hrs:min relative to imaging commencement within one hour of mock or IR treatment. Scale bars, 20 µm (representative of *n* = 2).

Supplementary Video 6 WAPL and RAD51 depletion rescue immediate mitotic death during through different mechanisms. Combined spinning disc confocal and DIC live imaging of G2-enriched mCherry–H2B HeLa cultures treated with the indicated siRNA and 14 Gy. The cells were synchronized at G1/S boundary using a double thymidine block, released and 14-Gy-irradiated 6 h later when the cells were in G2 (Fig. 6d). Time is relative to imaging commencement within 1 h of mock or IR treatment. Scale bars, 20 µm (representative of *n* = 3).

Supplementary Tables Supplementary Table 1. Primers for molecular cloning. Supplementary Table 2. Primers for quantitative PCR with reverse transcription. Supplementary Table 3. siRNAs used in this study.

## Source data

Source Data Figs. 1–8 and Extended Data Figs. 1–8 Statistical source data for all main figures and extended data figures with clearly named tabs for each relevant item.

Source Data Figs. 2, 3, 5, 7 and 8 and Extended Data Figs. 2, 3, 5, 6 and 7 Full-length unprocessed blots clearly labelled for each relevant figure.

## Data Availability

This paper analyses existing, publicly available TCGA data that can be located via http://firebrowse.org/ (LIHC, PAAD, BLCA, STAD, HNSC, BRCA, OV, LUSC, LUAD and PRAD). All other data supporting the findings of this study are available from the corresponding authors on reasonable request. Source data are provided with this paper.

## Code availability

All original code has been deposited via Zenodo at 10.5281/zenodo.1453559 (ref. ^77^) and is publicly available via GitHub at https://github.com/andrewdhawan/miRNA_hallmarks_of_cancer/blob/master/1_linear_model_miRNA_mRNA.R.

## References

[1] Vitale, I., Galluzzi, L., Castedo, M. & Kroemer, G. Mitotic catastrophe: a mechanism for avoiding genomic instability. *Nat. Rev. Mol. Cell Biol.* **12**, 385–392 (2011). 21527953 10.1038/nrm3115

[2] Galluzzi, L. et al. Molecular mechanisms of cell death: recommendations of the Nomenclature Committee on Cell Death 2018. *Cell Death Differ.* **25**, 486–541 (2018). 29362479 10.1038/s41418-017-0012-4 PMC5864239

[3] Masamsetti, V. P. et al. Replication stress induces mitotic death through parallel pathways regulated by WAPL and telomere deprotection. *Nat. Commun.* **10**, 4224 (2019). 31530811 10.1038/s41467-019-12255-w PMC6748914

[4] Chen, J. et al. Cell cycle checkpoints cooperate to suppress DNA- and RNA-associated molecular pattern recognition and anti-tumor immune responses. *Cell Rep.* **32**, 108080 (2020). 32877684 10.1016/j.celrep.2020.108080 PMC7530826

[5] Garner, E., Kim, Y., Lach, F. P., Kottemann, M. C. & Smogorzewska, A. Human GEN1 and the SLX4-associated nucleases MUS81 and SLX1 are essential for the resolution of replication-induced Holliday junctions. *Cell Rep.* **5**, 207–215 (2013). 24080495 10.1016/j.celrep.2013.08.041 PMC3844290

[6] Harding, S. M. et al. Mitotic progression following DNA damage enables pattern recognition within micronuclei. *Nature* **548**, 466–470 (2017). 28759889 10.1038/nature23470 PMC5857357

[7] Mackenzie, K. J. et al. cGAS surveillance of micronuclei links genome instability to innate immunity. *Nature* **548**, 461–465 (2017). 28738408 10.1038/nature23449 PMC5870830

[8] Feng, X. et al. ATR inhibition potentiates ionizing radiation-induced interferon response via cytosolic nucleic acid-sensing pathways. *EMBO J.* **39**, e104036 (2020). 32484965 10.15252/embj.2019104036 PMC7361286

[9] Scully, R., Panday, A., Elango, R. & Willis, N. A. DNA double-strand break repair-pathway choice in somatic mammalian cells. *Nat. Rev. Mol. Cell Biol.* **20**, 698–714 (2019). 31263220 10.1038/s41580-019-0152-0 PMC7315405

[10] Ceccaldi, R., Rondinelli, B. & D’Andrea, A. D. Repair pathway choices and consequences at the double-strand break. *Trends Cell Biol.* **26**, 52–64 (2016). 26437586 10.1016/j.tcb.2015.07.009 PMC4862604

[11] Llorens-Agost, M. et al. POLθ-mediated end joining is restricted by RAD52 and BRCA2 until the onset of mitosis. *Nat. Cell Biol.* **23**, 1095–1104 (2021). 34616022 10.1038/s41556-021-00764-0 PMC8675436

[12] Brambati, A. et al. RHINO directs MMEJ to repair DNA breaks in mitosis. *Science* **381**, 653–660 (2023). 37440612 10.1126/science.adh3694 PMC10561558

[13] Gelot, C. et al. Polθ is phosphorylated by PLK1 to repair double-strand breaks in mitosis. *Nature* **621**, 415–422 (2023). 37674080 10.1038/s41586-023-06506-6 PMC10499603

[14] Chiruvella, K. K., Liang, Z. & Wilson, T. E. Repair of double-strand breaks by end joining. *Cold Spring Harb. Perspect. Biol.* **5**, a012757 (2013). 23637284 10.1101/cshperspect.a012757 PMC3632057

[15] Bhargava, R., Onyango, D. O. & Stark, J. M. Regulation of single-strand annealing and its role in genome maintenance. *Trends Genet.* **32**, 566–575 (2016). 27450436 10.1016/j.tig.2016.06.007 PMC4992407

[16] Ramsden, D. A., Carvajal-Garcia, J. & Gupta, G. P. Mechanism, cellular functions and cancer roles of polymerase-theta-mediated DNA end joining. *Nat. Rev. Mol. Cell Biol.* **23**, 125–140 (2022). 34522048 10.1038/s41580-021-00405-2 PMC13537726

[17] Stark, J. M., Pierce, A. J., Oh, J., Pastink, A. & Jasin, M. Genetic steps of mammalian homologous repair with distinct mutagenic consequences. *Mol. Cell. Biol.* **24**, 9305–9316 (2004). 15485900 10.1128/MCB.24.21.9305-9316.2004 PMC522275

[18] Mateos-Gomez, P. A. et al. Mammalian polymerase theta promotes alternative NHEJ and suppresses recombination. *Nature* **518**, 254–257 (2015). 25642960 10.1038/nature14157 PMC4718306

[19] Johnson, R. D. & Jasin, M. Sister chromatid gene conversion is a prominent double-strand break repair pathway in mammalian cells. *EMBO J.* **19**, 3398–3407 (2000). 10880452 10.1093/emboj/19.13.3398 PMC313931

[20] Kadyk, L. C. & Hartwell, L. H. Sister chromatids are preferred over homologs as substrates for recombinational repair in Saccharomyces cerevisiae. *Genetics* **132**, 387–402 (1992). 1427035 10.1093/genetics/132.2.387 PMC1205144

[21] Buisson, R. et al. Coupling of homologous recombination and the checkpoint by ATR. *Mol. Cell* **65**, 336–346 (2017). 28089683 10.1016/j.molcel.2016.12.007 PMC5496772

[22] Ahlskog, J. K., Larsen, B. D., Achanta, K. & Sorensen, C. S. ATM/ATR-mediated phosphorylation of PALB2 promotes RAD51 function. *EMBO Rep.* **17**, 671–681 (2016). 27113759 10.15252/embr.201541455 PMC5341514

[23] Yang, H., Li, Q., Fan, J., Holloman, W. K. & Pavletich, N. P. The BRCA2 homologue Brh2 nucleates RAD51 filament formation at a dsDNA–ssDNA junction. *Nature* **433**, 653–657 (2005). 15703751 10.1038/nature03234

[24] Belan, O. et al. Visualization of direct and diffusion-assisted RAD51 nucleation by full-length human BRCA2 protein. *Mol. Cell* **83**, 2925–2940 e2928 (2023). 37499663 10.1016/j.molcel.2023.06.031 PMC7615647

[25] Forget, A. L. & Kowalczykowski, S. C. Single-molecule imaging brings Rad51 nucleoprotein filaments into focus. *Trends Cell Biol.* **20**, 269–276 (2010). 20299221 10.1016/j.tcb.2010.02.004 PMC2862779

[26] Wright, W. D., Shah, S. S. & Heyer, W. D. Homologous recombination and the repair of DNA double-strand breaks. *J. Biol. Chem.* **293**, 10524–10535 (2018). 29599286 10.1074/jbc.TM118.000372 PMC6036207

[27] Barber, L. J. et al. RTEL1 maintains genomic stability by suppressing homologous recombination. *Cell* **135**, 261–271 (2008). 18957201 10.1016/j.cell.2008.08.016 PMC3726190

[28] Bizard, A. H. & Hickson, I. D. The dissolution of double Holliday junctions. *Cold Spring Harb. Perspect. Biol.* **6**, a016477 (2014). 24984776 10.1101/cshperspect.a016477 PMC4067992

[29] Matos, J. & West, S. C. Holliday junction resolution: regulation in space and time. *DNA Repair* **19**, 176–181 (2014). 24767945 10.1016/j.dnarep.2014.03.013 PMC4065333

[30] Wyatt, H. D., Sarbajna, S., Matos, J. & West, S. C. Coordinated actions of SLX1–SLX4 and MUS81–EME1 for Holliday junction resolution in human cells. *Mol. Cell* **52**, 234–247 (2013). 24076221 10.1016/j.molcel.2013.08.035

[31] Chan, Y. W. & West, S. C. Spatial control of the GEN1 Holliday junction resolvase ensures genome stability. *Nat. Commun.* **5**, 4844 (2014). 25209024 10.1038/ncomms5844 PMC4172962

[32] Sakaue-Sawano, A. et al. Genetically encoded tools for optical dissection of the mammalian cell cycle. *Mol. Cell* **68**, 626–640 e625 (2017). 29107535 10.1016/j.molcel.2017.10.001

[33] Musacchio, A. The molecular biology of spindle assembly checkpoint signaling dynamics. *Curr. Biol.* **25**, R1002–R1018 (2015). 26485365 10.1016/j.cub.2015.08.051

[34] Santaguida, S., Tighe, A., D’Alise, A. M., Taylor, S. S. & Musacchio, A. Dissecting the role of MPS1 in chromosome biorientation and the spindle checkpoint through the small molecule inhibitor reversine. *J. Cell Biol.* **190**, 73–87 (2010). 20624901 10.1083/jcb.201001036 PMC2911657

[35] Sakaue-Sawano, A. et al. Visualizing spatiotemporal dynamics of multicellular cell-cycle progression. *Cell* **132**, 487–498 (2008). 18267078 10.1016/j.cell.2007.12.033

[36] Yim, E. K. & Park, J. S. The role of HPV E6 and E7 oncoproteins in HPV-associated cervical carcinogenesis. *Cancer Res. Treat.* **37**, 319–324 (2005). 19956366 10.4143/crt.2005.37.6.319 PMC2785934

[37] Leahy, J. J. et al. Identification of a highly potent and selective DNA-dependent protein kinase (DNA-PK) inhibitor (NU7441) by screening of chromenone libraries. *Bioorg. Med. Chem. Lett.* **14**, 6083–6087 (2004). 15546735 10.1016/j.bmcl.2004.09.060

[38] Feng, W. et al. Genetic determinants of cellular addiction to DNA polymerase theta. *Nat. Commun.* **10**, 4286 (2019). 31537809 10.1038/s41467-019-12234-1 PMC6753077

[39] Ochs, F. et al. 53BP1 fosters fidelity of homology-directed DNA repair. *Nat. Struct. Mol. Biol.* **23**, 714–721 (2016). 27348077 10.1038/nsmb.3251

[40] Bhat, K. P. & Cortez, D. RPA and RAD51: fork reversal, fork protection, and genome stability. *Nat. Struct. Mol. Biol.* **25**, 446–453 (2018). 29807999 10.1038/s41594-018-0075-z PMC6006513

[41] Lv, W., Budke, B., Pawlowski, M., Connell, P. P. & Kozikowski, A. P. Development of small molecules that specifically inhibit the D-loop activity of RAD51. *J. Med. Chem.* **59**, 4511–4525 (2016). 27049177 10.1021/acs.jmedchem.5b01762 PMC5473336

[42] Hauf, S. et al. The small molecule Hesperadin reveals a role for Aurora B in correcting kinetochore-microtubule attachment and in maintaining the spindle assembly checkpoint. *J. Cell Biol.* **161**, 281–294 (2003). 12707311 10.1083/jcb.200208092 PMC2172906

[43] Charrier, J. D. et al. Discovery of potent and selective inhibitors of ataxia telangiectasia mutated and Rad3 related (ATR) protein kinase as potential anticancer agents. *J. Med. Chem.* **54**, 2320–2330 (2011). 21413798 10.1021/jm101488z

[44] Daum, J. R. et al. Cohesion fatigue induces chromatid separation in cells delayed at metaphase. *Curr. Biol.* **21**, 1018–1024 (2011). 21658943 10.1016/j.cub.2011.05.032 PMC3119564

[45] Elmore, S. Apoptosis: a review of programmed cell death. *Toxicol. Pathol.* **35**, 495–516 (2007). 17562483 10.1080/01926230701320337 PMC2117903

[46] Igney, F. H. & Krammer, P. H. Death and anti-death: tumour resistance to apoptosis. *Nat. Rev. Cancer* **2**, 277–288 (2002). 12001989 10.1038/nrc776

[47] Lazebnik, Y. A., Kaufmann, S. H., Desnoyers, S., Poirier, G. G. & Earnshaw, W. C. Cleavage of poly(ADP-ribose) polymerase by a proteinase with properties like ICE. *Nature* **371**, 346–347 (1994). 8090205 10.1038/371346a0

[48] Wei, M. C. et al. Proapoptotic BAX and BAK: a requisite gateway to mitochondrial dysfunction and death. *Science* **292**, 727–730 (2001). 11326099 10.1126/science.1059108 PMC3049805

[49] Vitale, I., Manic, G., Castedo, M. & Kroemer, G. Caspase 2 in mitotic catastrophe: the terminator of aneuploid and tetraploid cells. *Mol. Cell Oncol.* **4**, e1299274 (2017). 28616577 10.1080/23723556.2017.1299274 PMC5462511

[50] Gascoigne, K. E. & Taylor, S. S. Cancer cells display profound intra- and interline variation following prolonged exposure to antimitotic drugs. *Cancer Cell* **14**, 111–122 (2008). 18656424 10.1016/j.ccr.2008.07.002

[51] Topham, C. H. & Taylor, S. S. Mitosis and apoptosis: how is the balance set? *Curr. Opin. Cell Biol.* **25**, 780–785 (2013). 23890995 10.1016/j.ceb.2013.07.003

[52] Flynn, P. J., Koch, P. D. & Mitchison, T. J. Chromatin bridges, not micronuclei, activate cGAS after drug-induced mitotic errors in human cells. *Proc. Natl Acad. Sci. USA* **118**, e2103585118 (2021). 34819364 10.1073/pnas.2103585118 PMC8640936

[53] Wu, J. & Chen, Z. J. Innate immune sensing and signaling of cytosolic nucleic acids. *Annu Rev. Immunol.* **32**, 461–488 (2014). 24655297 10.1146/annurev-immunol-032713-120156

[54] Hu, X., Li, J., Fu, M., Zhao, X. & Wang, W. The JAK/STAT signaling pathway: from bench to clinic. *Signal Transduct. Target Ther.* **6**, 402 (2021). 34824210 10.1038/s41392-021-00791-1 PMC8617206

[55] Rongvaux, A. et al. Apoptotic caspases prevent the induction of type I interferons by mitochondrial DNA. *Cell* **159**, 1563–1577 (2014). 25525875 10.1016/j.cell.2014.11.037 PMC4272443

[56] White, M. J. et al. Apoptotic caspases suppress mtDNA-induced STING-mediated type I IFN production. *Cell* **159**, 1549–1562 (2014). 25525874 10.1016/j.cell.2014.11.036 PMC4520319

[57] Sakai, W. et al. Functional restoration of BRCA2 protein by secondary BRCA2 mutations in BRCA2-mutated ovarian carcinoma. *Cancer Res.* **69**, 6381–6386 (2009). 19654294 10.1158/0008-5472.CAN-09-1178 PMC2754824

[58] Cesare, A. J., Hayashi, M. T., Crabbe, L. & Karlseder, J. The telomere deprotection response is functionally distinct from the genomic DNA damage response. *Mol. Cell* **51**, 141–155 (2013). 23850488 10.1016/j.molcel.2013.06.006 PMC3721072

[59] Yu, W. et al. Repair of G1 induced DNA double-strand breaks in S-G2/M by alternative NHEJ. *Nat. Commun.* **11**, 5239 (2020). 33067475 10.1038/s41467-020-19060-w PMC7567796

[60] Mladenov, E. et al. Strong suppression of gene conversion with increasing DNA double-strand break load delimited by 53BP1 and RAD52. *Nucleic Acids Res.* **48**, 1905–1924 (2020). 31832684 10.1093/nar/gkz1167 PMC7038941

[61] Zierhut, C. et al. The cytoplasmic DNA sensor cGAS promotes mitotic cell death. *Cell* **178**, 302–315 e323 (2019). 31299200 10.1016/j.cell.2019.05.035 PMC6693521

[62] Potters, L. et al. American Society for Therapeutic Radiology and Oncology and American College of Radiology practice guideline for the performance of stereotactic body radiation therapy. *Int. J. Radiat. Oncol. Biol. Phys.* **60**, 1026–1032 (2004). 15519771 10.1016/j.ijrobp.2004.07.701

[63] Beaton, L., Bandula, S., Gaze, M. N. & Sharma, R. A. How rapid advances in imaging are defining the future of precision radiation oncology. *Br. J. Cancer* **120**, 779–790 (2019). 30911090 10.1038/s41416-019-0412-y PMC6474267

[64] Demaria, S. et al. Radiation dose and fraction in immunotherapy: one-size regimen does not fit all settings, so how does one choose? *J. Immunother. Cancer* **9**, e002038 (2021). 33827904 10.1136/jitc-2020-002038 PMC8031689

[65] Oh, G. et al. POLQ inhibition elicits an immune response in homologous recombination-deficient pancreatic adenocarcinoma via cGAS/STING signaling. *J. Clin. Invest.* **133**, e165934 (2023). 36976649 10.1172/JCI165934 PMC10232002

[66] Lou, J. et al. Phasor histone FLIM-FRET microscopy quantifies spatiotemporal rearrangement of chromatin architecture during the DNA damage response. *Proc. Natl Acad. Sci. USA* **116**, 7323–7332 (2019). 30918123 10.1073/pnas.1814965116 PMC6462080

[67] Anderson, M. J., Casey, G., Fasching, C. L. & Stanbridge, E. J. Evidence that wild-type TP53, and not genes on either chromosome 1 or 11, controls the tumorigenic phenotype of the human fibrosarcoma HT1080. *Genes Chromosomes Cancer* **9**, 266–281 (1994). 7519049 10.1002/gcc.2870090407

[68] Crabbe, L., Cesare, A. J., Kasuboski, J. M., Fitzpatrick, J. A. & Karlseder, J. Human telomeres are tethered to the nuclear envelope during postmitotic nuclear assembly. *Cell Rep.* **2**, 1521–1529 (2012). 23260663 10.1016/j.celrep.2012.11.019 PMC3694759

[69] Van Ly, D. et al. Telomere loop dynamics in chromosome end protection. *Mol. Cell* **71**, 510–525 e516 (2018). 30033372 10.1016/j.molcel.2018.06.025

[70] Tigano, M., Vargas, D. C., Tremblay-Belzile, S., Fu, Y. & Sfeir, A. Nuclear sensing of breaks in mitochondrial DNA enhances immune surveillance. *Nature* **591**, 477–481 (2021). 33627873 10.1038/s41586-021-03269-w

[71] Brinkman, E. K. et al. Kinetics and fidelity of the repair of Cas9-induced double-strand DNA breaks. *Mol. Cell* **70**, 801–813 e806 (2018). 29804829 10.1016/j.molcel.2018.04.016 PMC5993873

[72] Brinkman, E. K., Chen, T., Amendola, M. & van Steensel, B. Easy quantitative assessment of genome editing by sequence trace decomposition. *Nucleic Acids Res.* **42**, e168 (2014). 25300484 10.1093/nar/gku936 PMC4267669

[73] Pierce, A. J., Johnson, R. D., Thompson, L. H. & Jasin, M. XRCC3 promotes homology-directed repair of DNA damage in mammalian cells. *Genes Dev.* **13**, 2633–2638 (1999). 10541549 10.1101/gad.13.20.2633 PMC317094

[74] Cesare, A. J., Heaphy, C. M. & O’Sullivan, R. J. Visualization of telomere integrity and function in vitro and in vivo using immunofluorescence techniques. *Curr. Protoc. Cytom.* **73**, 12.40.1–12.40.31 (2015). 26132175 10.1002/0471142956.cy1240s73 PMC4862373

[75] Hayashi, M. T., Cesare, A. J., Rivera, T. & Karlseder, J. Cell death during crisis is mediated by mitotic telomere deprotection. *Nature* **522**, 492–496 (2015). 26108857 10.1038/nature14513 PMC4481881

[76] Nassour, J. et al. Telomere-to-mitochondria signalling by ZBP1 mediates replicative crisis. *Nature* **614**, 767–773 (2023). 36755096 10.1038/s41586-023-05710-8 PMC9946831

[77] Dhawan, A. et al. Hallmarks of cancer. *Zenodo*10.5281/zenodo.1453559 (2018).

