# Supplementary material for: Homologous recombination promotes non-immunogenic mitotic cell death upon DNA damage
Source: Nat Cell Biol. 2025 Jan 13;27(1):59–72. doi: 10.1038/s41556-024-01557-x (PMC11735404; doi:10.1038/s41556-024-01557-x)
Supplement: Supplementary file 1 — Supplementary Fig. 1 [file 41556_2024_1557_MOESM1_ESM.pdf]

nature cell biology

Supplementary information https://doi.org/10.1038/s41556-024-01557-x

Homologous recombination promotes non-immunogenic mitotic cell death upon DNA damage

In the format provided by the authors and unedited

SUPPLEMENTARY FIGURE

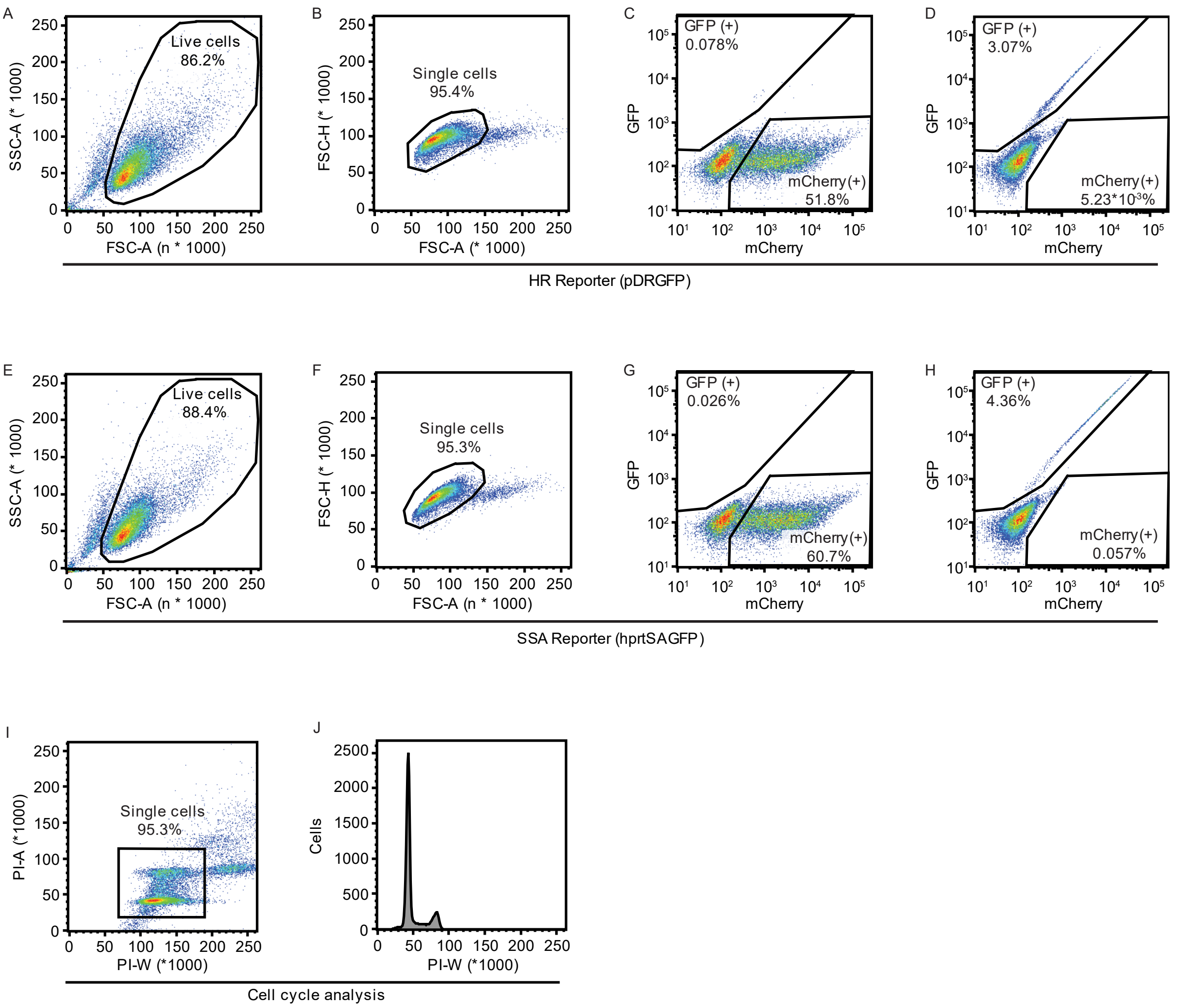

Supplementary Figure 1: Flow cytometry gating strategies. A-D) Homologous recombination (HR) reporter assays. For experiments using the pDRGFP reporter assay, outcomes were determined by gating A) live cell events followed by B) single cell events. Events were then gated for C) transfection efficiency using a generic mCherry expressing plasmid, or D) identified as GFP positive following transfection of an I-SceI expressing plasmid (representative of n = 3). **E-H)** Single strand annealing (SSA) reporter assays. For experiments using the hprtSAGFP reporter assay, outcomes were determined by gating E) live cell events followed by F) single cell events. Events were then gated for G) transfection efficiency using a generic mCherry expressing plasmid, or H) identified as GFP positive following transfection of an I-SceI expressing plasmid (representative of n = 3). **I, J)** Cell cycle analysis was performed by gating I) single cell events before J) plotting outcomes in a histogram (representative of n = 2). For all experiments > 10,000 events were collected for each sample measured.

SUPPLEMENTARY TABLES

Supplementary Table 1. Primers for molecular cloning

PRIMER	SEQUENCE (5' – 3')	SOURCE
H2B Forward	GATCCACCGGTCGCCACCATGCCAGAGCCAGCGAAG	This paper
H2B Reverse	ATTCCACAGCCGGATCTAAGCGCTGGTGTACTTGG	This paper
mCherry Forward	TCTAGGCGCCGGAATGGTGAGCAAGGGCGAGGAGG	This paper
mCherry Reverse	GGTGGCGACCGGTGGATCCTTGTACAGCTCGTCCATGC	This paper
pLXSN Forward	ATCCGGCTGTGGAATGTGTG	This paper
pLXSN Reverse	ATTCCGGCGCCTAGAGAAGG	This paper
crRNA sequence	GCCGATGGTGAAGTGGTAAG	Ref.71
LBR2 Forward	AAATGGCTGTCTTTCCCAGTAA	This paper
LBR2 Reverse	GTAGCCTTTCTGGCCCTAAAAT	Ref. 71

Supplementary Table 2. Primers for RT-qPCR

PRIMER	SEQUENCE (5' – 3')	SOURCE	IDENTIFIER
CCL5 Forward	ACAGCCTCTCCCACAGGTA	This paper	N/A
CCL5 Reverse	TGTGGTGTCCGAGGAATATGG		
cGAS Forward	AAGCAACTACGACTAAAGCCAT		
cGAS Reverse	GATAGCCGCCATGTTTCTTCTTG		
GAPDH Forward	AAGGTCGGAGTCAACGGATTTG		
GAPDH Reverse	TGAGGTCAATGAAGGGGTCAT		
GEN1 Forward	GGGAGTGAATGACTTGTGGCA		
GEN1 Reverse	CAGCTTTCAGCTTTGGTGGT		
IFIT1 Forward	CAGAACGGCTGCCTAATTTACA		
IFIT1 Reverse	TCCCACACTGTATTTGGTGTCT		
IFN1A Forward	GGAGGTTGTCAGAGCAGAAATC		
IFN1A Reverse	ATAGCAGGGGTGAGAGTCTTTG		
IFN1B Forward	TATGGGAGGATTCTGCATTACC		
IFN1B Reverse	GGCTAGGAGATCTTCAGTTTCG		
IRF3 Forward	CTCGTGATGGTCAAGGTTGTG		
IRF3 Reverse	AATGTGCAGGTCCACAGTATTC		
ISG15 Forward	TCTTTGCCAGTACAGGAGCTT		
ISG15 Reverse	CAGGGACACCTGGAATTCGTT		
ISG54 Forward	GAAGATTTCTGAAGAGTGCAGC		
ISG54 Reverse	ATCAAGTTCCAGGTGAAATGGC		
LIG4 Forward	CTGGAACTGTATTGCCTGCTTT		
LIG4 Reverse	TGATGAATCTTCTCGTTTAACTGG		
MAVS Forward	AGAGAGAAGGAGCCAAGTTACC		
MAVS Reverse	AGGGCTTGCTCTGAATTCTCT		
MDA5 Forward	ATGCAACCAGAGAAGATCCATT		
MDA5 Reverse	AAACACGTTCTTTGCGATTTCC		
OAS1 Forward	CTGGATTCTGCTGGCTGAAAG		
OAS1 Reverse	TGTGCTGGGTCTATGAGAGAAA		
POLθ Forward	CTGCTGTTTATGCAGGGATGAT		
POLθ Reverse	CACAGTATGAAAGCCAGAAGCA		
RAD52 Forward	GCTCAGTGTTATGCTTTGGACA		
RAD52 Reverse	CCTCAATGTAGCACACCTTCTG		
SLX4 Forward	ATGGCGGGGAATGTGTATGG		
SLX4 Reverse	GGTCACGTTCATGGCTGAGA		
WAPL Forward	GATTCCCAGCACCATCAGAATC		
WAPL Reverse	GAAGCATCTTGTTCCAGTTCCA		
RIG-I Forward	TGCGAATCAGATCCCAGTGTA	Reference 70	N/A
RIG-I Reverse	TGCCTGTAACTCTATACCCATGT		

Supplementary Table 3. siRNAs used in this study

siRNA	SOURCE	IDENTIFIER
ON-TARGETplus Non-targeting Control SMARTpool	Dharmacon	D-001810-10
ON-TARGETplus Human BRCA2 SMARTpool	Dharmacon	L-003462-00
ON-TARGETplus Human DDX58 (RIG-I) SMARTpool	Dharmacon	L-012511-00
ON-TARGETplus Human GEN1 SMARTpool	Dharmacon	L-018757-02-0005
ON-TARGETplus Human IFIH1 (MDA5) SMARTpool	Dharmacon	L-013041-00
ON-TARGETplus Human IRF3 SMARTpool	Dharmacon	L-006875-00
ON-TARGETplus Human LIG4 SMARTpool	Dharmacon	L-004254-00
ON-TARGETplus Human MAVS SMARTpool	Dharmacon	L-024237-00
ON-TARGETplus Human MB21D1 (cGAS) SMARTpool	Dharmacon	L-015607-02
ON-TARGETplus Human PALB2 SMARTpool	Dharmacon	L-012928-01
ON-TARGETplus Human POLθ SMARTpool	Dharmacon	L-015180-01
ON-TARGETplus Human PRKDC (DNA-PKcs) SMARTpool	Dharmacon	L-005030-00
ON-TARGETplus Human RMI2 SMARTpool	Dharmacon	L-015684-01-0005
ON-TARGETplus Human RTEL1 SMARTpool	Dharmacon	L-013379-00-0005
ON-TARGETplus Human SLX4 SMARTpool	Dharmacon	L-014895-00-0005
ON-TARGETplus Human WAPL SMARTpool	Dharmacon	L-026287-01
Silencer Select Negative Control #1	Ambion; Thermo Fisher Scientific	4390843
Silencer Select Human RAD51	Ambion; Thermo Fisher Scientific	4392420, s11735
Silencer Select Human RAD52	Ambion; Thermo Fisher Scientific	4392420, s11747
