# Supplementary material for: Homologous recombination promotes non-immunogenic mitotic cell death upon DNA damage
Source: Nat Cell Biol. 2025 Jan 13;27(1):59–72. doi: 10.1038/s41556-024-01557-x (PMC11735404; doi:10.1038/s41556-024-01557-x)
Supplement: Supplementary file 2 — Reporting Summary [file 41556_2024_1557_MOESM2_ESM.pdf]

nature portfolio

Corresponding author(s): Anthony Cesare and Harriet Gee

Last updated by author(s): Sep 26, 2024

Reporting Summary

Nature Portfolio wishes to improve the reproducibility of the work that we publish. This form provides structure for consistency and transparency in reporting. For further information on Nature Portfolio policies, see our Editorial Policies and the Editorial Policy Checklist.

Statistics

For all statistical analyses, confirm that the following items are present in the figure legend, table legend, main text, or Methods section.

n/a	Confirmed	
☐	☒	The exact sample size (*n*) for each experimental group/condition, given as a discrete number and unit of measurement
☐	☒	A statement on whether measurements were taken from distinct samples or whether the same sample was measured repeatedly
☐	☒	The statistical test(s) used AND whether they are one- or two-sided<br>*Only common tests should be described solely by name; describe more complex techniques in the Methods section.*
☒	☐	A description of all covariates tested
☐	☒	A description of any assumptions or corrections, such as tests of normality and adjustment for multiple comparisons
☐	☒	A full description of the statistical parameters including central tendency (e.g. means) or other basic estimates (e.g. regression coefficient) AND variation (e.g. standard deviation) or associated estimates of uncertainty (e.g. confidence intervals)
☐	☒	For null hypothesis testing, the test statistic (e.g. *F*, *t*, *r*) with confidence intervals, effect sizes, degrees of freedom and *P* value noted<br>*Give P values as exact values whenever suitable.*
☒	☐	For Bayesian analysis, information on the choice of priors and Markov chain Monte Carlo settings
☒	☐	For hierarchical and complex designs, identification of the appropriate level for tests and full reporting of outcomes
☒	☐	Estimates of effect sizes (e.g. Cohen's *d*, Pearson's *r*), indicating how they were calculated

Our web collection on statistics for biologists contains articles on many of the points above.

Software and code

Policy information about availability of computer code

Data collection	Live imaging - IncuCyte Zoom v2019B (Sartorius), Zen Blue v2 or 2.6 (ZEISS), Cell Voyager CV1000 (Yokogawa)<br>RT-qPCR: QuantStudio™ Real-Time PCR Software v1.7.2 (Applied Biosystems; Thermo Fisher Scientific)<br>Flow cytometry: FACSDiva 6.1.3 (BD Biosciences)<br>RAD51 foci analysis: Zen Blue v2.3 pro (ZEISS)<br>Cytogenetic analysis: Metafer4 v3.12.8
Data analysis	Live imaging - IncuCyte Zoom v2019B (Sartorius), Zen Blue v3.5 (ZEISS), Cell Voyager CV1000 (Yokogawa)<br>RT-qPCR: Microsoft Excel v2308<br>Flow cytometry: FlowJo v10.8.0<br>RAD51 foci analysis: CellProfiler v4.2.1<br>Cytogenetic analysis: ISIS v5.8.8<br>TIDE analysis: TIDE software (https://tide.nki.nl)<br>TCGA analysis: ppcor package v1.1 and R v3.5 (both available on https://cran.r-project.org/)<br>Statistical analysis: GraphPad Prism v9.3.1<br>Figure preparation: Adobe Illustrator v27.9 and Photoshop v25.0<br><br>All original code has been deposited at Zenodo and is publicly available here: https://github.com/andrewdhawan/miRNA_hallmarks_of_cancer/blob/master/1_linear_model_miRNA_mRNA.R (DOI: 10.5281/zenodo.1453559) (DOI: 10.5281/zenodo.1453559).

For manuscripts utilizing custom algorithms or software that are central to the research but not yet described in published literature, software must be made available to editors and reviewers. We strongly encourage code deposition in a community repository (e.g. GitHub). See the Nature Portfolio guidelines for submitting code & software for further information.

Data

Policy information about availability of data

All manuscripts must include a data availability statement. This statement should provide the following information, where applicable:

- Accession codes, unique identifiers, or web links for publicly available datasets
- A description of any restrictions on data availability
- For clinical datasets or third party data, please ensure that the statement adheres to our policy

This paper analyzes existing, publicly available TCGA data that can be located here: http://firebrowse.org/ (LIHC, PAAD, BLCA, STAD, HNSC, BRCA, OV, LUSC, LUAD, PRAD). Blot and quantitative source data have been provided in Source Data. All other data supporting the findings of this study are available from the corresponding authors on reasonable request.

Research involving human participants, their data, or biological material

Policy information about studies with human participants or human data. See also policy information about sex, gender (identity/presentation), and sexual orientation and race, ethnicity and racism.

Reporting on sex and gender	N/A
Reporting on race, ethnicity, or other socially relevant groupings	N/A
Population characteristics	N/A
Recruitment	N/A
Ethics oversight	N/A

Note that full information on the approval of the study protocol must also be provided in the manuscript.

Field-specific reporting

Please select the one below that is the best fit for your research. If you are not sure, read the appropriate sections before making your selection.

☒ Life sciences ☐ Behavioural & social sciences ☐ Ecological, evolutionary & environmental sciences

For a reference copy of the document with all sections, see nature.com/documents/nr-reporting-summary-flat.pdf

Life sciences study design

All studies must disclose on these points even when the disclosure is negative.

Sample size	No statistical method was applied to predetermine sample size. Sample sizes are consistent with published studies using similar experimentation (Hayashi et al, Nature 2015; Masamsetti et al Nature Communications 2019, Nassour et al Nature 2023) and are reported in the figure legends.
Data exclusions	No data were excluded.
Replication	Experiments were performed with biologically consistent outcomes across 8 cell lines in multiple independent replicates as described in the figure legends.
Randomization	Samples were randomly allocated into experimental groups. Data analysis was randomized as follows. In live imaging experiments, cells within the field of view were selected randomly prior to analysis and followed for the experiential duration. For cytogenetic analysis, chromosome spreads were chosen at random and captured through automated imaging. For quantitation of RAD51 foci, all interphase cells completely contained within the field views were analyzed using automated methods.
Blinding	Researchers were not blinded due to impracticality.

Reporting for specific materials, systems and methods

We require information from authors about some types of materials, experimental systems and methods used in many studies. Here, indicate whether each material, system or method listed is relevant to your study. If you are not sure if a list item applies to your research, read the appropriate section before selecting a response.

Materials & experimental systems

n/a	Involved in the study
☐	☒ Antibodies
☐	☒ Eukaryotic cell lines
☒	☐ Palaeontology and archaeology
☒	☐ Animals and other organisms
☒	☐ Clinical data
☒	☐ Dual use research of concern
☒	☐ Plants

Methods

n/a	Involved in the study
☒	☐ ChIP-seq
☐	☒ Flow cytometry
☒	☐ MRI-based neuroimaging

Antibodies

Antibodies used

Within the methods section "Western Blotting"
Primary antibodies used in the study: β-Actin (AC-15, Sigma-Aldrich, A5441, 1:10,000), β-Tubulin (Abcam, ab6046; 1:500), BCL-xL (54H6, Cell Signaling Technology, 2764, 1:1000), BRCA2 (Ab-1, clone 2B, Sigma-Aldrich, OP95, 1:1000), Caspase-2 (C2, Cell Signalling Technology, 2224, 1:1000), Caspase-8 (D35G2, Cell Signalling Technology, 4790, 1:1000), CHK1 (2G1D5, Cell Signaling Technology, 2360, 1:1000), CHK1-pS345 (133D3, Cell Signaling Technology, 2348, 1:1000), Cleaved Caspase-3 (Asp175, Cell Signaling Technology, 9661, 1:500), Cleaved Caspase-8 (Asp384, 11G10, Cell Signaling Technology, 9748, 1:1000), Cyclin A2 (EPR17351, Abcam, ab181591, 1:3000), Cyclin B1 (D5C10, Cell Signaling Technology, 12231, 1:2000), DNA-PKcs (Y393, Abcam, ab32566, 1:1000), GAPDH (D16H11, Cell Signaling Technology, 5174, 1:5000), Histone H2B (V119, Cell Signaling Technology, 8135, 1:1000), Histone H3-pS10 (D2C8, Cell Signaling Technology, 2224, 1:1000), IFIT1 (D2X9Z, Cell Signaling Technology, 14769, 1:1000), ISG15 (Cell Signaling Technology, 2743, 1:1000), LIG4 (EPR16531, Abcam, ab193353, 1:1000), MCL-1 (Y37, Abcam, ab32087, 1:1000), NOXA (D8L7U, Cell Signaling Technology, 14766, 1:1000), PALB2 (Bethyl, A301-246A, 1:1000), PARP (46D11, Signaling Technology, 9532, 1:1000), POLθ (Thermo Fisher Scientific, PA5-69577, 1:250), STAT1 (Cell Signaling Technology, 9172, 1:1000, STAT1-pY701 (58D6, Cell Signaling Technology, 9167, 1:1000), RAD51 (14B4. Novus, NB100-148, 1:500), RAD52 (F-7, Santa Cruz, sc-365341, 1:500), RMI2 (Thermo Fisher Scientific, PA5-95632, 1:1000), RTEL1 (Novus, NBP2-22360 1:1000), Vinculin (hVIN-1, Sigma-Aldrich, V9131, 1:10,000), and WAPL (A-7, Santa Cruz, sc-365189, 1:1000). Secondary antibodies used in the study: Goat Anti-Mouse HRP (Dako, P0447, 1:5000 – 1:20,000) and Goat Anti-Rabbit HRP (Dako, P0448, 1:5000 – 1:20,000).

Within the methods section "RAD51 foci labelling"
primary anti-RAD51 antibody (Calbiochem, PC130, 1:500), Alexa Fluor 568-conjugated secondary antibody (Invitrogen, A11036, 1:500). Secondary antibodies used in the study: Alexa-568 goat anti-mouse (A11031, Invitrogen, 1:10,000).

Validation

1. Antibodies validated in this study using siRNA depletion or CRISPR deletion: RAD51 (14B4. Novus, NB100-148), RAD52 (F-7, Santa Cruz, sc-365341), BRCA2 (Ab-1, clone 2B, Sigma-Aldrich, OP95), PALB2 (Bethyl, A301-246A), WAPL (A-7, Santa Cruz, sc-365189), DNA-PKcs (Y393, Abcam, ab32566), LIG4 (EPR16531, Abcam, ab193353), POLθ (Thermo Fisher Scientific, PA5-69577), RTEL1 (Novus, NBP2-22360), RMI2 (Thermo Fisher Scientific, PA5-95632), STAT1 (Cell Signaling Technology, 9172), Caspase-8 (D35G2, Cell Signalling Technology, 4790), Caspase-2 (C2, Cell Signalling Technology, 2224).

2. Antibodies validated previously through controlled induction and suppression of a DNA damage response: CHK1-pS345 (133D3, Cell Signaling Technology, 2348) validated for western blot detection in human samples by Lamm et al. (2020): https://doi.org/10.1038/s41556-020-00605-6.

3. Antibodies validated through induction of a well described phenotype using relevant biological stimulus, suitable for western blotting in human samples:
- PARP (46D11, Signaling Technology, 9532) antibody according to the manufacturer "recognizes a full-length PARP-1 and its cleaved form (89 kDa) produced by Caspase mediated cleavage during apoptotic cell death". Validated for western blot detection in human samples by the manufacturer: https://www.cellsignal.com/products/primary-antibodies/parp-46d11-rabbit-mab/9532

- Cleaved Caspase-3 Asp175, Cell Signaling Technology, 9661) antibody according to the manufacturer "detects endogenous levels of the large fragment (17/19 kDa) of activated Caspase-3 resulting from cleavage adjacent to Asp175". Validated for western blot detection in human samples by the manufacturer: https://www.cellsignal.com/products/primary-antibodies/cleaved-caspase-3-asp175-antibody/9661

- Cleaved Caspase-8 (Asp384, 11G10, Cell Signaling Technology, 9748) antibody according to the manufacturer "detects endogenous levels of the small fragment of activated Caspase-8 resulting from cleavage at Asp384". Validated for western blot detection in human samples by the manufacturer: https://www.cellsignal.com/products/primary-antibodies/cleaved-caspase-8-asp384-11g10-mouse-mab/9748

- STAT1-pY701 (58D6, Cell Signaling Technology, 9167) antibody according to the manufacturer "detects endogenous levels of Stat1 only when phosphorylated at tyrosine 701. The antibody detects phosphorylated tyrosine 701 of p91 Stat1 and also the p84 splice variant". Validated for western blot detection in human samples by the manufacturer: https://www.cellsignal.com/products/primary-antibodies/phospho-stat1-tyr701-58d6-rabbit-mab/9167. Additionally, STAT1 phosphorylation on Y701 following IR was validated using whole cell extracts from human cells through western blotting by Harding et al. (2017): https://doi.org/10.1038/nature23470

- Histone H3-pS10 (D2C8, Cell Signaling Technology) antibody detects phosphorylation of histone H3 on serine 10 that correlates with chromosome condensation during mitosis. Validated for western blot detection in human samples by the manufacturer: https://www.cellsignal.com/products/primary-antibodies/phospho-histone-h3-ser10-d2c8-xp-rabbit-mab/3377

- NOXA (D8L7U, Cell Signaling Technology, 14766) antibody validated for western blot detection in human samples following TPA

treatment or its overexpression by the manufacturer: https://www.cellsignal.com/products/primary-antibodies/noxa-d8l7u-rabbit-mab/14766

- IFIT1 (D2X9Z, Cell Signaling Technology, 14769) antibody validated for western blot detection in human samples by the manufacturer: https://www.cellsignal.com/products/primary-antibodies/ifit1-d2x9z-rabbit-mab/14769

- ISG15 (Cell Signaling Technology, 2743) antibody validated for western blot detection in human samples by the manufacturer: https://www.cellsignal.com/products/primary-antibodies/isg15-antibody/2743

- MCL-1 (Y37, Abcam, ab32087) antibody recognizes MCL-1 that undergoes gradual degradation during mitotic death as show by Haschka et al. (2015): https://doi.org/10.1038/ncomms7891. Validated for western blot detection in human samples using genetic knockout by the manufacturer: https://www.abcam.com/en-us/products/primary-antibodies/mcl1-antibody-y37-ab32087#

- BCL-xL (54H6, Cell Signaling Technology, 2764) antibody recognizes BCL-xL that is phosphorylated during mitotic death as shown by Haschka et al. (2015): https://doi.org/10.1038/ncomms7891. Validated for western blot detection in human samples using siRNA depletion by the manufacturer: https://www.cellsignal.com/products/primary-antibodies/bcl-xl-54h6-rabbit-mab/2764

4. Antibodies validated by the manufacturer for immunofluorescent detection in human samples: RAD51 (Calbiochem, PC130): https://www.merckmillipore.com/AU/en/product/Anti-Rad51-Ab-1-Rabbit-pAb,EMD_BIO-PC130

5. Commonly used loading controls validated by the manufactures for western blotting using human samples:
- Histone H2B (V119, Cell Signaling Technology, 8135): https://www.cellsignal.com/products/primary-antibodies/histone-h2b-v119-antibody/8135
- GAPDH (D16H11, Cell Signaling Technology, 5174): https://www.cellsignal.com/products/primary-antibodies/gapdh-d16h11-xp-rabbit-mab/5174
- Vinculin (Sigma-Aldrich, V9131) validated through miR depletion by the manufacturer: https://www.sigmaaldrich.com/catalog/product/sigma/v9131
- β-Actin (AC-15, Sigma-Aldrich, A5441): https://www.sigmaaldrich.com/AU/en/product/sigma/a5441
- β-Tubulin (Abcam, ab6046): https://www.abcam.com/en-us/products/primary-antibodies/beta-tubulin-antibody-loading-control-ab6046#

6. Commonly used commercial secondary antibodies: Goat anti-mouse HRP (P044701, DAKO Agilent); Goat anti-Rabbit HRP (P044801, DAKO Agilent); Alexa-568 goat anti-mouse (A11031, Invitrogen)

Eukaryotic cell lines

Policy information about cell lines and Sex and Gender in Research

Cell line source(s)

H2B-eGFP HeLa (PMID: 30918123), 2-color FUCCI (2F) IMR90 (PMID: 31530811), and BAX and BAK double knockout (DKO) HeLa from a parental CCL-2 strain (PMID: 31530811), were all created previously. We obtained the following cell lines from: HeLa (human, female; RRID: CVCL_0030), Megan Chircop (CMRI, Sydney, Australia); HT1080 6TG (human, male; RRID: N/A; PMID: 7519049), Eric Stanbridge (University of California, Irvine, US); IMR90 E6E7 (human, female; RRID: N/A; PMID: 26108857), Jan Karlseder (Salk Institute, La Jolla US); HCT116 p53 KO (human, male; RRID: CVCL_HD97), Bert Vogelstein (Johns Hopkins University, Baltimore, US); T98G (human, male; RRID: CVCL_0556) and A549 (human, male; RRID:CVCL_0023), Roger Reddel (CMRI); PEO1 (human, female; RRID: CVCL_2686) and PEO4 (human, female; RRID: CVCL_2690), Anna DeFazio (WIMR, Sydney, Australia); Phoenix-AMPHO (human, female; RRID:CVCL_H716), ATCC.

Three color (3F) FUCCI cells were created using the tFucci(CA)2/pCSII-EF (RDB15446)(PMID: 29107535) construct kindly provided by Dr Hiroyuki Miyoshi (Keio University) through RIKEN BRC. To generate Caspase 2 knockdown and corresponding control lines, HeLa cells were transduced at CMRI with 3rd generation lentivectors harbouring Caspase-2 (Sigma-Aldrich, TRCN0000003508; targeted sequence GTTGAGCTGTGACTACGACTT) or control shRNAs (Addgene plasmid # 1864; http://n2t.net/addgene:1864 ; RRID:Addgene_1864, was a gift from David Sabatini).

Authentication

As described in Methods, Cell Culture: The identity and purity of all cell lines was verified by Cell Bank Australia using short tandem repeat (STR) profiling.

Mycoplasma contamination

All cultures were routinely mycoplasma tested and found to be negative (MycoAlert, LT07-118, Lonza).

Commonly misidentified lines
(See ICLAC register)

None of this cell lines in this study are included in the ICLAC register of misidentified lines.

Flow Cytometry

Plots

Confirm that:

☒ The axis labels state the marker and fluorochrome used (e.g. CD4-FITC).

☒ The axis scales are clearly visible. Include numbers along axes only for bottom left plot of group (a 'group' is an analysis of identical markers).

☒ All plots are contour plots with outliers or pseudocolor plots.

☒ A numerical value for number of cells or percentage (with statistics) is provided.

Methodology

Sample preparation	Methods, DSB repair reporter lines: The DSB repair reporter vectors pDRGFP (Addgene plasmid #26475; http://n2t.net/addgene:26475; RRID: Addgene_26475) and hprtSAGFP (Addgene plasmid #41594; http://n2t.net/addgene:41594; RRID: Addgene_41594), were a gift from Maria Jasin. DSB reporter cell lines were generated by transfection of HeLa cells with reporter constructs using Lipofectamine 3000 (Invitrogen Thermo Fisher Scientific, L3000015) according to the manufacturer's instructions. Stable integration of reporter constructs (containing a puromycin resistance gene) was confirmed by selection with puromycin (1µg/ml), which was added 48 hours after transfection and maintained in cell media for 2 weeks.<br><br>To measure DSB repair, reporter cell lines were transfected with pCBASce (Addgene plasmid #26477; http://n2t.net/addgene:26477; RRID: Addgene_26477, a gift from Maria Jasin) plasmid using Lipofectamine 3000 according to the manufacturer's instructions, resulting in production of the endonuclease I-SceI. I-SceI induces a DSB within stably integrated reporter constructs, and subsequent GFP expression acts as a measure of DSB repair (PMID: 15485900; PMID: 10541549). Reporter cells were transfected in parallel with pCAGGS-mCherry (a gift from Phil Sharp; Addgene plasmid #41583; http://n2t.net/addgene:41583; RRID: Addgene_41583) as an internal control to estimate transfection efficiency and calculate relative DSB repair activity. At 48 hours post transfection, cells were collected and GFP and mCherry expression quantified on FACSCantoII (BD Biosciences) using FACSDiva 6.1.3 software and analyzed using FlowJo v10.8.0. Relative DSB repair capacity was calculated as a ratio of the GFP positive cells (%) to mCherry positive cells (%) and normalized to a control condition (equal 1). Representative gating strategies are shown in Supplementary Figure 1.<br><br>Methods, Cell cycle analysis by flow cytometry<br>Cells were fixed in ice-cold 70% ethanol and immediately processed or stored overnight at – 20 °C. Cells were pelleted, washed in PBS, and resuspended in a propidium iodide (1mg/ml) and RNase A (0.5mg/ml; Qiagen, 1007885) staining solution. Cell cycle distribution was quantified on FACSCantoII (BD Biosciences) using FACSDiva 6.1.3 software and analyzed using FlowJo v10.8.0. Representative gating strategies are shown in Supplementary Figure 1.
Instrument	FACSCantoII (BD Biosciences)
Software	Data collected using FACSDiva 6.1.3 software and analyzed using FlowJo v10.8.0.
Cell population abundance	We sorted HeLa and HT1080 3-colour FUCCI, and HeLa mCherry-H2B, on mCherry. Appropriate cell-cycle dependent coloration for the FUCCI cells, or mcherry-H2B expression, were confirmed by live imaging.
Gating strategy	As shown in Supplementary Figure 1: For experiments using the pDRGFP or hprtSAGFP reporter assays, live cell events were gated based on SSC-A and FSC-A (A, E), followed by single cell events gated based on FSC-H and FSC-A (B, F). Events were then gated for transfection efficiency using a generic mCherry expressing plasmid (C, G), or identified as GFP positive following transfection of an I-SceI expressing plasmid (D, H).<br>Cell cycle analysis was performed by gating single cell events based on PI-A and PI-W (I) before plotting outcomes in a histogram (J).<br>For all experiments >20,000 events were collected for each sample measured.

☒ Tick this box to confirm that a figure exemplifying the gating strategy is provided in the Supplementary Information.
