# Supplementary material for: Homologous recombination promotes non-immunogenic mitotic cell death upon DNA damage
Source: Nat Cell Biol. 2025 Jan 13;27(1):59–72. doi: 10.1038/s41556-024-01557-x (PMC11735404; doi:10.1038/s41556-024-01557-x)

Figure 2D

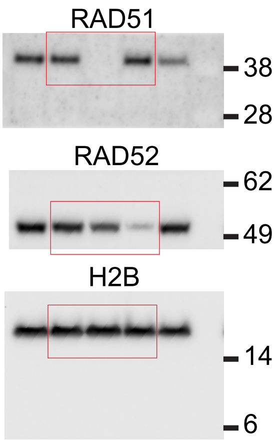

Figure 3A

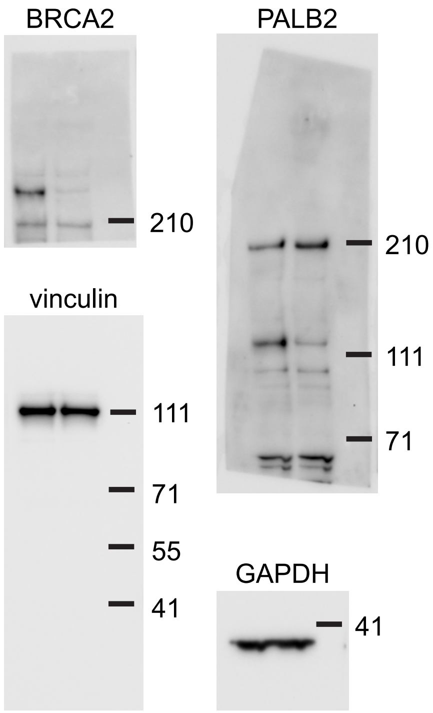

Figure 3D

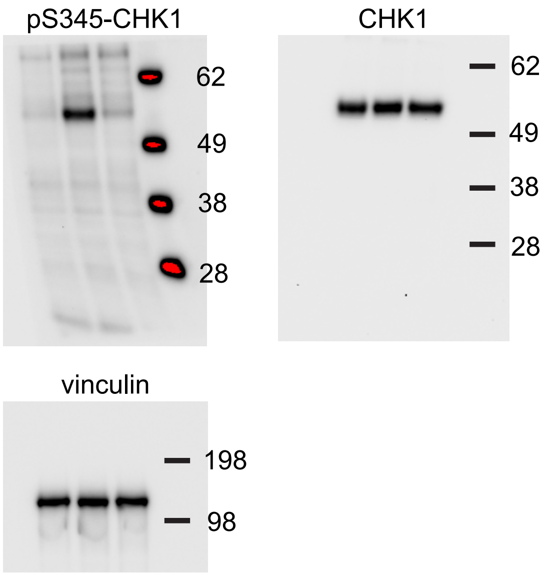

Figure 5C

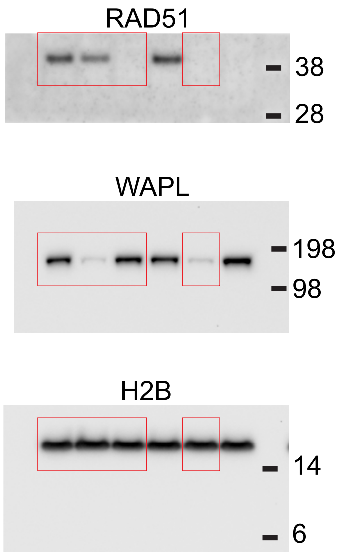

Figure 7B

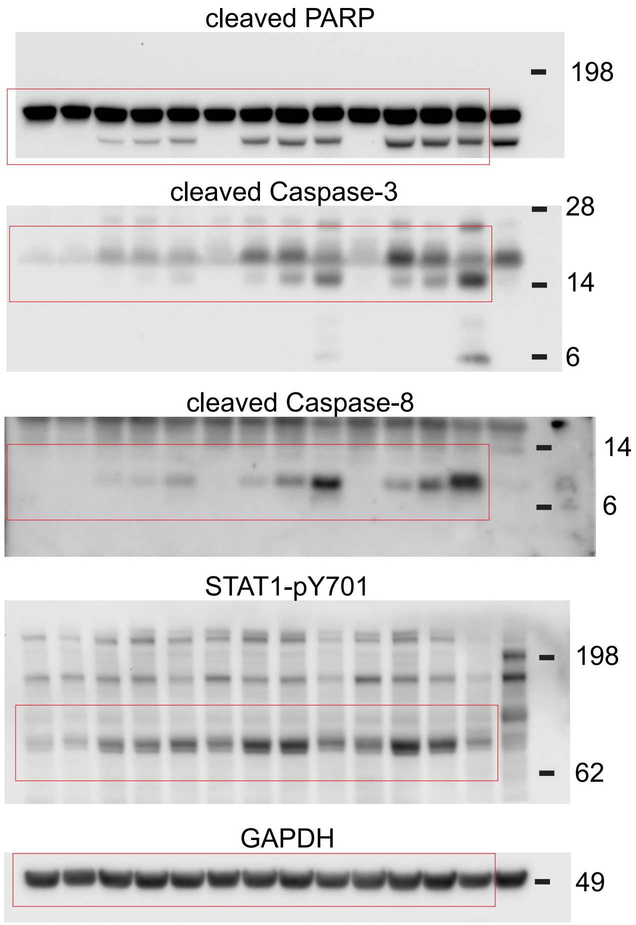

Figure 7F

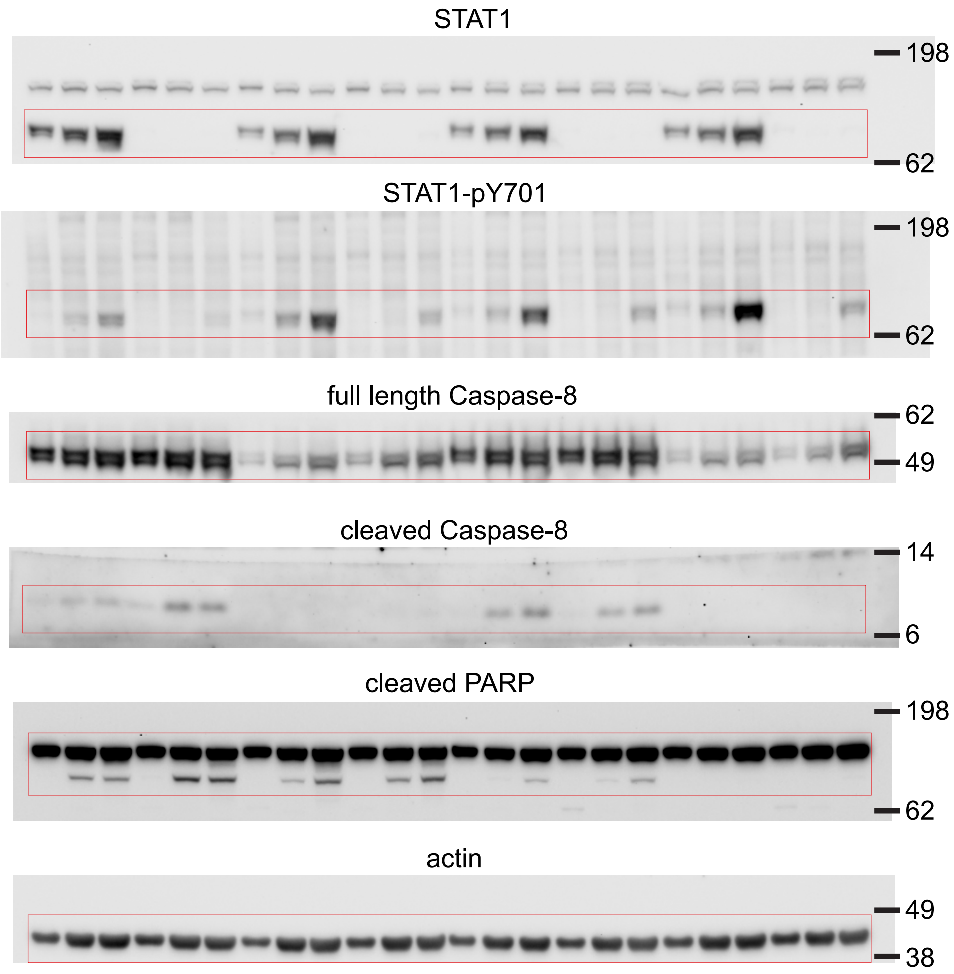

Figure 7G

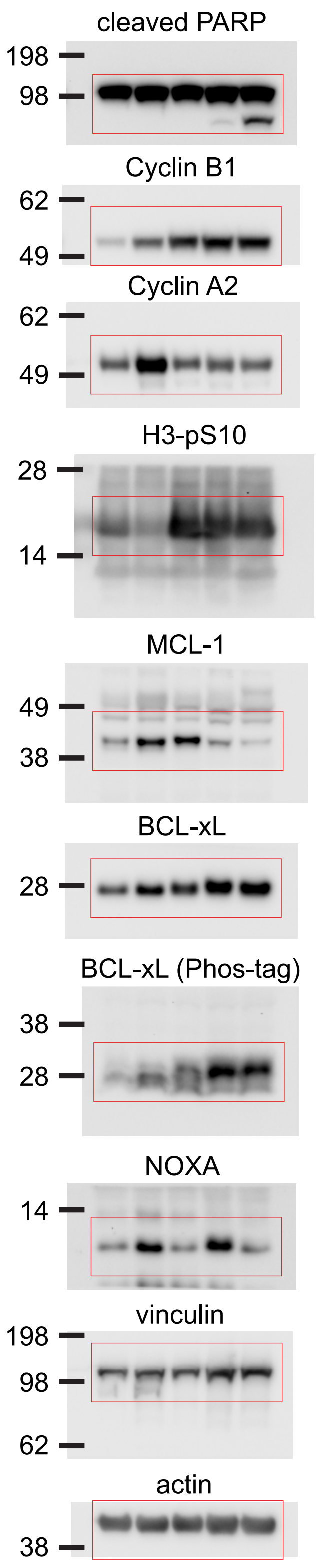

Figure 8B

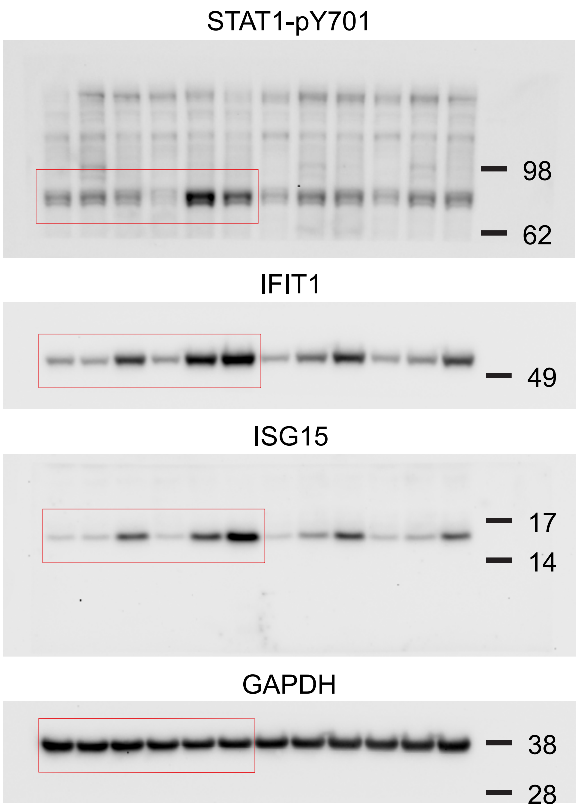

Extended Data Fig. 2C

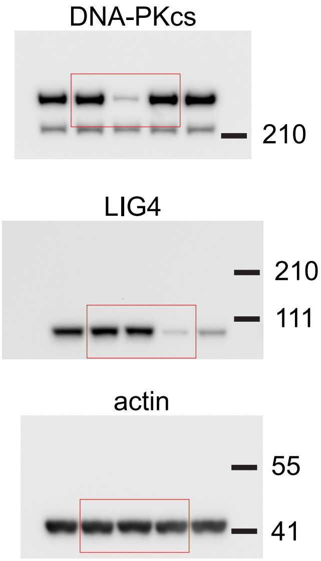

Extended Data Fig. 3A

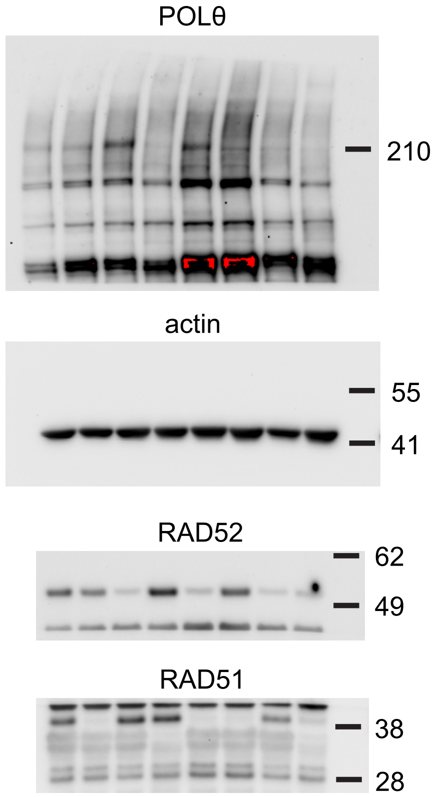

Extended Data Fig. 3B

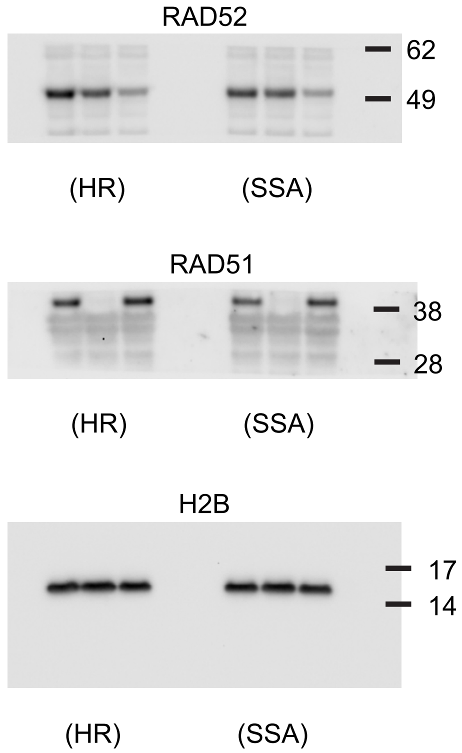

Extended Data Fig. 5A

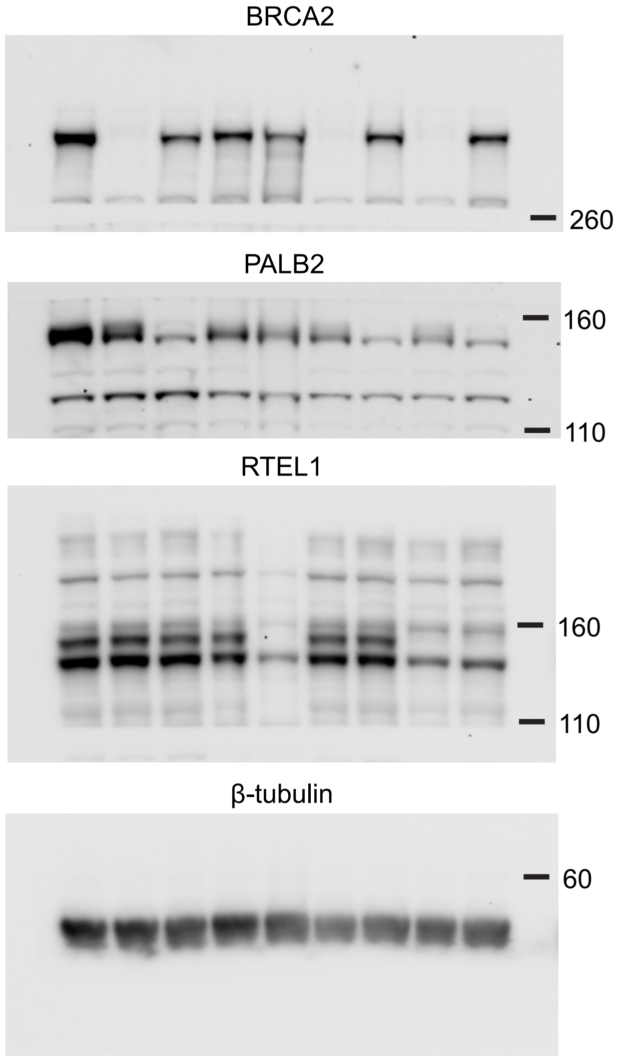

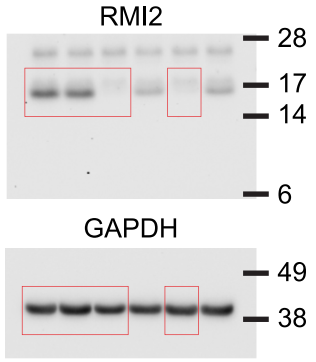

Extended Data Fig. 6C

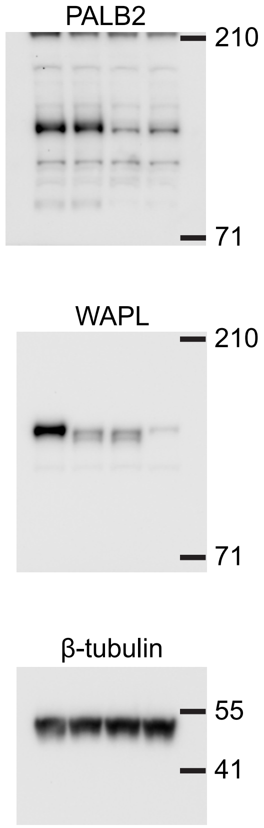

Extended Data Fig. 7C

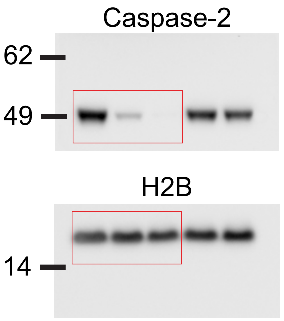

Supplement: Supplementary file 12 — Full-length unprocessed blots clearly labelled for each relevant figure. [file 41556_2024_1557_MOESM12_ESM.pdf]
