# Supplementary material for: Homologous recombination promotes non-immunogenic mitotic cell death upon DNA damage
Source: Nat Cell Biol. 2025 Jan 13;27(1):59–72. doi: 10.1038/s41556-024-01557-x (PMC11735404; doi:10.1038/s41556-024-01557-x)
Supplement: Supplementary file 3 — Peer Review File [file 41556_2024_1557_MOESM3_ESM.pdf]

nature portfolio

Peer Review File

Homologous recombination promotes non-immunogenic mitotic cell death upon DNA damage

Corresponding Author: Professor Anthony Cesare

Parts of this Peer Review File have been redacted as indicated to maintain the confidentiality of unpublished data.

Version 0:

Decision Letter:

*Please delete the link to your author homepage if you wish to forward this email to co-authors.

Dear Professor Cesare,

Thank you again for submitting your manuscript, "Homologous recombination promotes mitotic death to suppress the innate immune response", to Nature Cell Biology. It has now been seen by 3 referees, who are experts in DNA repair (Referee #1); mitosis (Referee #2); and cell death (Referee #3). As you will see from their comments (attached below), they found this work of potential interest but have raised substantial concerns, which in our view would need to be addressed with considerable revisions before we can consider publication in Nature Cell Biology.

As per our standard process, we have now discussed the referee reports in detail within the editorial team, including the chief editor, to identify key referee points that should be addressed with priority, and requests that are overruled as being beyond the scope of the current study. To guide the scope of the revisions, I have listed these points below. Our standard revision period is six months, and we are committed to providing a fair and constructive peer-review process, so please feel free to contact me if you would like to discuss any of the referee comments further or if you anticipate any issues or delays addressing the reviews.

The reviewers provided consistent feedback that the conclusions were not currently sufficiently strongly supported by the data. We found their points valid and significant and stress that they would need to be addressed with experiments and data. Reconsideration of the study for this journal and re-engagement of referees will depend on the strength of these revisions. In our view, it is essential to dedicate efforts in revision to address the following issues:

A- All reviewers felt that the phenotypic analyses of mitosis were too limited, and in particular, mitotic duration and its impact on cell death need to be studied more carefully.

Rev#1 point #3
Rev#2 main point #1 and minor points #1, #3
Rev#3 “I encourage the authors to look more carefully into the link between mitotic duration, slippage and cell death rates, as it would potentially explain some of their observations…” paragraph

B- The implication of particular DNA repair pathways and dHJs requires further evidence, as per the reviewers, and further work should better tie in these data with the analyses of chromosome aberrations:
Rev#1 points #2-3
Rev#2 point #3, #4, #6

C- The analyses connecting DNA repair pathways to mitotic cell death and the cell death apparatus need to be stronger and conclusions adjusted to better reflect the data, including with regards to the induction of innate immune gene products and ISGs, as opposed to an innate immune response:
Rev#1 point #4
Rev#3 paragraphs “There is no mechanistic insight, how HR (or inhibition of NHEJ) would “promote” mitotic..”; “Moreover, statements like “The innate immune response promotes delayed lethality” are not supported by the data shown,..”; “The

authors propose that the delayed death in G1 is mediated by caspase-8,..” and ‘Last but not least, the authors build their claims on "suppression of innate immunity by HR"..”

D- Please attend to all the reviewers’ technical concerns, minor points, and suggestions to strengthen existing data, including Rev#3’s request for validation of key data in another model and Rev#2’s concern about the over-reliance on siRNA (#2)

E- Finally, please pay close attention to our guidelines on statistical and methodological reporting (listed below) as failure to do so may delay the reconsideration of the revised manuscript. In particular, please provide:

- a Supplementary Figure including unprocessed images of all gels/blots in the form of a multi-page pdf file. Please ensure that blots/gels are labeled and the sections presented in the figures are clearly indicated.

- a Supplementary Table including all numerical source data in Excel format, with data for different figures provided as different sheets within a single Excel file. The file should include source data giving rise to graphical representations and statistical descriptions in the paper and for all instances where the figures present representative experiments of multiple independent repeats, the source data of all repeats should be provided.

We would be happy to consider a revised manuscript that would satisfactorily address these points, unless a similar paper is published elsewhere, or is accepted for publication in Nature Cell Biology in the meantime.

When revising the manuscript please:

- ensure that it conforms to our format instructions and publication policies (see below and https://www.nature.com/nature/for-authors).

- provide a point-by-point rebuttal to the full referee reports verbatim, as provided at the end of this letter.

- provide the completed Reporting Summary (found here https://www.nature.com/documents/nr-reporting-summary.pdf). This is essential for reconsideration of the manuscript will be available to editors and referees in the event of peer review. For more information see http://www.nature.com/authors/policies/availability.html or contact me.

When submitting the revised version of your manuscript, please pay close attention to our href="https://www.nature.com/nature-portfolio/editorial-policies/image-integrity">Digital Image Integrity Guidelines. and to the following points below:

-- that unprocessed scans are clearly labelled and match the gels and western blots presented in figures.
-- that control panels for gels and western blots are appropriately described as loading on sample processing controls
-- all images in the paper are checked for duplication of panels and for splicing of gel lanes.

Finally, please ensure that you retain unprocessed data and metadata files after publication, ideally archiving data in perpetuity, as these may be requested during the peer review and production process or after publication if any issues arise.

Nature Cell Biology is committed to improving transparency in authorship. As part of our efforts in this direction, we are now requesting that all authors identified as ‘corresponding author’ on published papers create and link their Open Researcher and Contributor Identifier (ORCID) with their account on the Manuscript Tracking System (MTS), prior to acceptance. ORCID helps the scientific community achieve unambiguous attribution of all scholarly contributions. You can create and link your ORCID from the home page of the MTS by clicking on ‘Modify my Springer Nature account’. For more information please visit please visit www.springernature.com/orcid.

This journal strongly supports public availability of data. Please place the data used in your paper into a public data repository, or alternatively, present the data as Supplementary Information. If data can only be shared on request, please explain why in your Data Availability Statement, and also in the correspondence with your editor. Please note that for some data types, deposition in a public repository is mandatory - more information on our data deposition policies and available repositories appears below.

Please submit the revised manuscript files and the point-by-point rebuttal to the referee comments using this link:

Link Redacted

*This url links to your confidential home page and associated information about manuscripts you may have submitted or be reviewing for us. If you wish to forward this email to co-authors, please delete the link to your homepage.

We hope that you will find our referees' comments and editorial guidance helpful. Please do not hesitate to contact me if there is anything you would like to discuss. Thank you again for considering our journal for your work.

Best wishes,

Melina

Melina Casadio, PhD
Senior Editor, Nature Cell Biology
ORCID ID: https://orcid.org/0000-0003-2389-2243

Reviewers' Comments:

Reviewer #1:
Remarks to the Author:

The authors present a detailed story that implicates DNA repair mechanism as a key determinant of mitotic cell death, which dictates pattern recognition of missegregated chromosomal DNA. Based on supportive data, the authors propose that HR driven double Holliday Junctions elicit mitotic cell death and this suppresses pattern recognition by cGAS and MAVs. Moreover, ATR and cohesion fatigue contributes to these responses. BRCA2, PALB2, and RAD51 knockdown in S/G2 irradiated cells prevents mitotic cell death and led to increased inflammatory signaling. Conversely, DNA-PK inhibition, or knockdown of Lig4, RAD52, or Pol theta increased it in a manner that depended on Rad51. Knockdown of SLX4 or RTEL1 is used as evidence for dHJs being the toxic lesion that causes mitotic cell death. Cohesion fatigue due to prolonged mitotic duration promoted mitotic cell death, while WAPL knockdown prevented mitotic cell death and increased inflammatory signaling.

This is an interesting study that brings a new perspective to the understand how genomic instability communicates with innate immune signaling. An intriguing concept put forth by the authors is that unresolved HR intermediates are the cause of mitotic cell death and suppress inflammatory signals. This data may help explain prior observations that NHEJ loss prevents cGAS-STING signaling following IR. They also invoke suppression of HR as a means by which ATRi increases inflammatory signaling. However, there are aspects of the model that are insufficiently supported at this point and examples in the literature that may conflict with the model. The authors should resolve these issues in a revised manuscript.

1. The authors show that Pol theta loss increases mitotic cell death and prevents inflammatory signaling. Pol theta inhibitors have been shown to increase cGAS-STING signaling. The authors should discuss this in light of their own data.
2. The data to support dHJs as critical to mitotic cell death and suppression of cGAS responses is insufficiently supported. The authors show that SLX4 and RTEL1 loss increases mitotic cell death and suppresses inflammatory signals. There are several ways this model could be better tested. BLM helicase, SLX4, and GEN1 are more involved in dissolving/resolving dHJs. Knockdown of these factors alone, and in combination should be performed rather than RTEL1. In addition, expression of the bacterial HJ resolvase RuvC could be used to see if this suppresses the response. RUVC has ben expressed in human cells and can resolve HJ intermediates PMID: 34776211
3. An alternative explanation to several findings is that cells without ligase 4 have reduced mitotic progression due to excessive damage that results in prolonged G2 arrest. In addition, that ATR inhibition increases inflammatory signaling by premature entry into mitosis and chromosome missegregation rather than suppression of HR. The authors should delineate whether some of their results can be explained by this rather than by mitotic cell death and include discussion in the text.
4. MPS1 inhibitors increase chromosome missegregation and inflammatory signals. Do mitotic checkpoint errors bypass this proposed dHJ mitotic cell death checkpoint mechanisms?

Reviewer #2:
Remarks to the Author:
In their article entitled “Homologous recombination promotes mitotic death to suppress the innate immune response”, Szmyd et al. present evidence that specific homologous recombination repair of DSBs can lead to mitotic arrest and subsequent cell death. In contrast, other repair pathways do not lead to this phenomenon. Because these repair pathways are cell cycle regulated, the timing of DSB formation and repair can impact the behaviour of cells during subsequent mitoses. This is an interesting and stimulating manuscript that has the potential to be an important contribution to our understanding of the cellular consequences of DNA damage. However, in its current version, the manuscript suffers somewhat from an over-reliance on siRNA, which makes especially the interpretation of double-knockdowns and effect sizes difficult. Furthermore, I think that a possible involvement of the length of mitotic arrest is currently not investigated sufficiently. Overall, the points outlined below would need to be addressed before publication.

Major points:
1. A major working model of mitotic arrest is that the slow degradation of Cyclin B competes with a build-up of apoptotic activity for determining cell fate as long as the spindle checkpoint cannot be satisfied (PMID 18656424). Therefore, mitotic cell death frequently is a function of time. A stronger spindle checkpoint stimulus will keep cells arrested for longer, and

therefore potentially lead to a higher fraction of cells dying in mitosis. It would therefore be interesting to establish how mitotic arrest duration is affected by the authors' treatments. For many experiments, this information is provided, but for many experiments it is not (for example Fig 1G, 2B, 2E, etc.), and for some experiments (like Fig. 3C), it is not clear if the timing plots are done for all cells or deconvolved for radiation within a specific cell cycle stage. A further reason for doing this is that some DNA damage response factors like CHK2, ATR, BRCA1, BRCA2 are thought to potentially have functions in activating/counteracting the SAC (see for example PMID 20364141, 22340495, 29170278). Furthermore, specific repair defects could have varying impact on the strength of the SAC. For example, it looks from the analysis of Fig. 3C that the increased survival of siRAD51, BRCA2, PALB2 might primarily come from a subset of cells that do not arrest anymore. Timing is also relevant for Fig. 5 - perhaps the more severe chromosome alignment defects correlate with or cause a longer mitotic arrest, thereby allowing for more time to die. It would be great if this could systematically be assessed, as it may impact on the conclusions the authors would draw/the working model they can derive from their experiments. Importantly, I believe that the outcome of these experiments wouldn't impact the importance of the overall conclusions the authors make.
2. There is somewhat of an over-reliance on siRNA. In general, I don't think every single experiment needs to be repeated with CRISPR, but especially for double mutants, this should to be done. For example, when discussing the results of Fig. 4E-H/S4G, the authors make conclusions about Rad51 and WAPL having "independent mechanisms of mitotic death rescue", based on siRNA-mediated epistasis analysis. However, the combinatorial effects of incomplete depletion of two proteins can be very hard to predict, and it is possible that what they observe are additive effects of partial depletion of two proteins that are in the same pathway.
3. Whilst not entirely likely (at least not for all factors), there is a possibility that at least some of the DNA repair factors can carry out DNA repair functions during mitosis, and exert their critical function therefore during mitosis, and not the preceding interphase. This is not addressed by the siRNA experiments. Where available, chemical inhibitors could be used to address this (ie, according to the model, addition in mitosis should not give a phenotype, but addition during the relevant cell cycle stage should mimic loss throughout the cell cycle).
4. I am slightly confused about the chromosome aberration data (Fig. 4/S4). Are radials/chromatid fusions primarily made by S/G2 repair and therefore HR, and are multi-centric/circular chromosomes primarily made in G1 by NHEJ? If so, this should be explained and referenced (and ideally also shown by the authors own experiments by irradiating G1 vs S/G2 cells).
5. The data on cohesion fatigue: It looks like siRAD51 and control have similar amounts of cohesion defects (Fig. 4F), but there are differences in mitotic arrest duration/survival. Does that not mean that cohesion defects are unlikely to be the cause of mitotic arrest/survival differences? It seems to me that the simplest hypothesis to explain the impact of WAPL loss is that it impacts on the strength of the spindle checkpoint (either by leading to the generation of different DNA repair structures that do not generate as strong a SAC signal, or by directly impacting the SAC). Indeed, the data from Fig. 4H indicate that a substantial fraction of cells depleted for WAPL don't arrest in mitosis at all following irradiation). This should be discussed at the very least. In addition, some information seems to be missing – for example it does not seem to be indicated when the snapshot of Fig. 4F was taken. Similarly, the kinetics of cohesion fatigue are not shown. Personally, I would suggest removing the cohesion fatigue aspect from the manuscript, as it seems to dilute the main point of the study.
6. The model from Fig. 7E is a bit confusing. Does it suggest that there is a subset of DSB repair products that cause mitotic arrest? Or is the subset of DSBs whose repair isn't finished by the time cells enter mitosis critical? If the latter, can mitotic lethality be rescued by extending G2? This can be tested by addition of RO3306 to cells for various periods of time before washout and release into mitosis. (Note that this experiment might require careful addition of RO3306 specifically after S phase, as previous work (PMID 35443178) indicated that RO3306 can cause replication stress in certain contexts).

Minor points:
1. The usage of the term "mitotic catastrophe". This is a problematic term, as it can refer to many different things. For example, Vitale et al. (PMID 21527953) say "…'mitotic catastrophe' has been widely used to describe a form of cell death affecting higher eukaryotes, and several attempts have been made to precisely define mitotic catastrophe. For instance, mitotic catastrophe has been delineated as a type of cell death that: results from abnormal mitosis and is associated with spontaneous premature chromosome condensation and the formation of large cells with multiple micronuclei; derives from premature or inappropriate entry of cells into mitosis; or occurs either during or shortly after a dysregulated or failed mitosis.". Because this is such an imprecise term, and refers to so many discrete phenomena, I would therefore prefer it if the authors used more precise terms like "mitotic cell death" and "delayed interphase cell death" (or similar) for the bulk of the manuscript. (although it is obviously fine to discuss "mitotic catastrophe" in the introduction and discussion, and where necessary in the results).
2. The Rad51 foci number plots (like Fig. 3E) are often so small that is hard to see the relevant data.
3. To interpret the mitotic death experiments with the combination of siRAD52 and VE-822 addition would require the controls from Fig. S3B/C to also be carried out with a combination treatment.
4. I'm slightly confused about Fig. S6F – it looks like loss of MAVS, cGAS, IRF3 gets rid of the first peak of apoptosis, which I understand represents mitotic death in the first cell cycle after IR, but then in Fig. 6J, it looks like death is not impacted in the first mitosis.
5. When discussing Fig 6I/J, the authors refer to a possible discrepancy to PMID 31299200. The authors should point out that that study used a different means of causing mitotic arrest (spindle poisons, APC inhibitor) in contrast to DNA damage, and different cell line models. This could be the underlying reasons for the apparent discrepancy.
6. There is a typo in line 1095

Reviewer #3:
Remarks to the Author:
In their study, Szmyd and colleague establish a correlation between different DNA repair pathways activated in response to

irradiation damage, cell cycle status and the propensity of p53-deficient model cancer cells (HeLa) to die either in the next mitosis, along the intrinsic mitochondrial pathway (BAX/BAK mediated), or later in the next G1 phase, after experiencing mitotic defects and slippage triggering CIN, by BAX/BAK independent mechanisms involving caspase-8 (presumably extrinsic apoptosis). The latter phenomenon coincides with increased mRNA expression of inflammatory genes prior cell death, induced by the asymmetric distribution of DNA content and cytosolic nucleic acid sensing (presumably due to micronuclei formation and their breakdown, and/or the accumulation of extra centrosomes). The authors conclude that the DNA-repair machineries dictate cell fate and that a combination of radiotherapy with certain inhibitors of DNA repair pathways may improve response rates (due to the activation of innate immunity pathways) and that the response of cancer patients that show certain DNA repair deficiencies can be better guided based on this new knowledge.

By live-cell imaging of HeLa cells expressing the three factor FUCCI system, allowing the discrimination of cells in S, G2/M and G1 phase, in combination with different strategies to perturb various DNA repair pathways, the authors provide strong evidence that the homology repair (HR) machinery promotes mitotic cell death in cells that experience double strand breaks while in S or G2 phase, while Inhibition of the HR pathway allows cell survival along with escape to the next G1 phase and activation of inflammatory target genes, prior to delayed cell death. In contrast, error prone repair pathways, such as non-homologous end joining (NHEJ) or single strand annealing (SSA), allow cells that experience DSBs in G1 to pass through mitosis, at the expense of increasing rates of DNA segregation errors, promoting inflammation. Suppressing these error prone repair pathways and favouring activation of HR components, promotes mitotic cell death, while inhibiting HR pathways favours escape from mitotic arrest and inflammatory gene expression, along with delayed cell death.

Together these experiments establish an highly interesting interrelation between mitotic and “postmitotic” cell death, the cell cycle phase DSBs are experienced, and the respective repair machineries that are subsequently activated. As pointed out in their discussion, these observations may have clinical implications, pending such interdependencies are also operative in cancerous tissues of patients.

I believe the study is technically sound and the experiments are well-designed and (mostly) elegantly controlled. My main critique arises from the very DNA repair-centric view of things and the conclusions drawn, that are not always substantiated by the data shown. The claims made are simply too bold, starting with the title and continuing throughout the manuscript. This is simply misleading and actually not needed, as the data is very interesting.

Main concerns.

There is no mechanistic insight, how HR (or inhibition of NHEJ) would “promote” mitotic death, nor how non-HR repair pathways would suppress it. I rather believe that many of the phenomena observed might be explained by the time cells spend in mitosis rather than DNA repair pathways “dictating” if cells die or not, and how. Direct connections between DNA repair genes and the cell death machinery are not established in any of the paradigms tested, which is a clear shortcoming of an otherwise very elegant study.

I encourage the authors to look more carefully into the link between mitotic duration, slippage and cell death rates, as it would potentially explain some of their observations. Activation of BAX/BAK in mitosis depends clearly on inhibition and degradation of BCL2 family proteins, most notably BCLX and MCL1, respectively (at least in HeLa). MCL1 acts as a molecular timer in arrested cells, underlining the competing network model put forward by Taylor and colleagues. Interfering with HR may facilitate slippage that promotes cell survival, while inhibiting NHEJ may lead to extended mitotic arrest and death in mitosis (PMID: 18656424; PMID: 29987118; PMID: 23890995; PMID: 25922916, PMID: 29459486). Changes of MCL1 & BIM/NOXA levels or BCLX-phospho status could be assessed in some experimental settings to make a better connection to the BCL2 network in response to the different perturbations tested. I would also like to encourage the authors to discuss observations made that suggest G2 checkpoint override after IR damage by inhibition of CHK1 (and ATRi) promotes cell death in HeLa cells (PMID: 18510930, PMID: 22854598), while here the opposite phenomenon is observed. Maybe I miss an aspect that would explain this discrepancy.

Moreover, statements like “The innate immune response promotes delayed lethality” are not supported by the data shown, I feel, as there is no experiment conducted that would document that claim. Also, to consider Fludarabine as a selective STAT1 inhibitor is a bit of stretch. At the very best, what the authors show is that escape from mitotic arrest increases cell survival rates in the presence of CIN (an established phenomenon), that promotes inflammatory gene expression downstream of sensing of nucleic acids (also known for a while and confirmed here). Whether this in the end really triggers an “innate immune response” in its actual sense, has not been tested in this study. Maybe the authors could state that they see signs of sterile inflammation, based on their own data, but not more.

The authors propose that the delayed death in G1 is mediated by caspase-8, based on the use of Z-IETD-fmk, and that this death requires STAT1 signaling, presumably downstream of IFN receptors, using Fludarabine as an inhibitor of STAT1. While I deem Fludarabine to be highly unspecific, exerting a range of effects, I would also dare to say that 40µM of IETD will block other caspases, next to caspase-8; e.g. maybe caspase-2, implicated in mitotic catastrophe since a long time. Also, while all other data is beautifully quantified, here, “relative apoptosis” is shown, which makes it hard to judge what this means in real numbers. So, I suggest to provide some solid experimental evidence and deplete caspase-8 (which HeLa cells tolerate, as they don't express RIP3), to show this death is C8 dependent.

Last but not least, the authors build their claims on "suppression of innate immunity by HR" on the induction of a number of ISGs mRNAs when HR is blocked, but they never show that these transcripts are translated into protein. Given that the peak in mRNA of ISGs is seen at times where cells have fully activated caspases, that can block translation, e.g. by cleaving

translation initiation factors, there is reason to doubt that these proteins are expressed and secreted in order promote sterile inflammation, but I am happy to see data that argues against my point of view.

Finally, it would be also nice, if some of the key findings are confirmed in an additional model cell line, not just the overall trend, as shown in figure 1H.

GUIDELINES FOR MANUSCRIPT SUBMISSION TO NATURE CELL BIOLOGY

READABILITY OF MANUSCRIPTS – Nature Cell Biology is read by cell biologists from diverse backgrounds, many of whom are not native English speakers. Authors should aim to communicate their findings clearly, explaining technical jargon that might be unfamiliar to non-specialists, and avoiding non-standard abbreviations. Titles and abstracts should concisely communicate the main findings of the study, and the background, rationale, results and conclusions should be clearly explained in the manuscript in a manner accessible to a broad cell biology audience. Nature Cell Biology uses British spelling.

MANUSCRIPT FORMAT – please follow the guidelines listed in our Guide to Authors regarding manuscript formats at Nature Cell Biology.

TITLE – should be no more than 100 characters including spaces, without punctuation and avoiding technical terms, abbreviations, and active verbs..

AUTHOR NAMES – should be given in full.

AUTHOR AFFILIATIONS – should be denoted with numerical superscripts (not symbols) preceding the names. Full addresses should be included, with US states in full and providing zip/post codes. The corresponding author is denoted by: "Correspondence should be addressed to [initials]."

ABSTRACT AND MAIN TEXT – please follow the guidelines that are specific to the format of your manuscript, as listed in our Guide to Authors (http://www.nature.com/ncb/pdf/ncb_gta.pdf) Briefly, Nature Cell Biology Articles, Resources and Technical Reports have 3500 words, including a 150 word abstract, and the main text is subdivided in Introduction, Results, and Discussion sections. Nature Cell Biology Letters have up to 2500 words, including a 180 word introductory paragraph (abstract), and the text is not subdivided in sections.

ACKNOWLEDGEMENTS – should be kept brief. Professional titles and affiliations are unnecessary. Grant numbers can be listed.

AUTHOR CONTRIBUTIONS – must be included after the Acknowledgements, detailing the contributions of each author to the paper (e.g. experimental work, project planning, data analysis etc.). Each author should be listed by his/her initials.

FINANCIAL AND NON-FINANCIAL COMPETING INTERESTS – the authors must include one of three declarations: (1) that they have no financial and non-financial competing interests; (2) that they have financial and non-financial competing interests; or (3) that they decline to respond, after the Author Contributions section. This statement will be published with the article, and in cases where financial and non-financial competing interests are declared, these will be itemized in a web supplement to the article. For further details please see https://www.nature.com/licenceforms/nrg/competing-interests.pdf.

REFERENCES – are limited to a total of 70 for Articles, Resources, Technical Reports; and 40 for Letters. This includes references in the main text and Methods combined. References must be numbered sequentially as they appear in the main text, tables and figure legends and Methods and must follow the precise style of Nature Cell Biology references. References only cited in the Methods should be numbered consecutively following the last reference cited in the main text. References only associated with Supplementary Information (e.g. in supplementary legends) do not count toward the total reference limit and do not need to be cited in numerical continuity with references in the main text. Only published papers can be cited, and each publication cited should be included in the numbered reference list, which should include the manuscript titles. Footnotes are not permitted.

METHODS – Nature Cell Biology publishes methods online. The methods section should be provided as a separate Word document, which will be copyedited and appended to the manuscript PDF, and incorporated within the HTML format of the paper.

Methods should be written concisely, but should contain all elements necessary to allow interpretation and replication of the results. As a guideline, Methods sections typically do not exceed 3,000 words. The Methods should be divided into

subsections listing reagents and techniques. When citing previous methods, accurate references should be provided and any alterations should be noted. Information must be provided about: antibody dilutions, company names, catalogue numbers and clone numbers for monoclonal antibodies; sequences of RNAi and cDNA probes/primers or company names and catalogue numbers if reagents are commercial; cell line names, sources and information on cell line identity and authentication. Animal studies and experiments involving human subjects must be reported in detail, identifying the committees approving the protocols. For studies involving human subjects/samples, a statement must be included confirming that informed consent was obtained. Statistical analyses and information on the reproducibility of experimental results should be provided in a section titled “Statistics and Reproducibility”.

All Nature Cell Biology manuscripts submitted on or after March 21 2016 must include a Data availability statement as a separate section after Methods but before references, under the heading "Data Availability”. . For Springer Nature policies on data availability see http://www.nature.com/authors/policies/availability.html; for more information on this particular policy see http://www.nature.com/authors/policies/data/data-availability-statements-data-citations.pdf. The Data availability statement should include:

• Accession codes for primary datasets (generated during the study under consideration and designated as "primary accessions") and secondary datasets (published datasets reanalysed during the study under consideration, designated as "referenced accessions"). For primary accessions data should be made public to coincide with publication of the manuscript. A list of data types for which submission to community-endorsed public repositories is mandated (including sequence, structure, microarray, deep sequencing data) can be found here http://www.nature.com/authors/policies/availability.html#data.

• Unique identifiers (accession codes, DOIs or other unique persistent identifier) and hyperlinks for datasets deposited in an approved repository, but for which data deposition is not mandated (see here for details http://www.nature.com/sdata/data-policies/repositories).

• At a minimum, please include a statement confirming that all relevant data are available from the authors, and/or are included with the manuscript (e.g. as source data or supplementary information), listing which data are included (e.g. by figure panels and data types) and mentioning any restrictions on availability.

• If a dataset has a Digital Object Identifier (DOI) as its unique identifier, we strongly encourage including this in the Reference list and citing the dataset in the Methods.

We recommend that you upload the step-by-step protocols used in this manuscript to the Protocol Exchange. More details can found at www.nature.com/protocolexchange/about.

DISPLAY ITEMS – main display items are limited to 6-8 main figures and/or main tables for Articles, Resources, Technical Reports; and 5 main figures and/or main tables for Letters. For Supplementary Information see below.

FIGURES – Colour figure publication costs $600 for the first, and $300 for each subsequent colour figure. All panels of a multi-panel figure must be logically connected and arranged as they would appear in the final version. Unnecessary figures and figure panels should be avoided (e.g. data presented in small tables could be stated briefly in the text instead).

All imaging data should be accompanied by scale bars, which should be defined in the legend.
Cropped images of gels/blots are acceptable, but need to be accompanied by size markers, and to retain visible background signal within the linear range (i.e. should not be saturated). The boundaries of panels with low background have to be demarked with black lines. Splicing of panels should only be considered if unavoidable, and must be clearly marked on the figure, and noted in the legend with a statement on whether the samples were obtained and processed simultaneously. Quantitative comparisons between samples on different gels/blots are discouraged; if this is unavoidable, it should only be performed for samples derived from the same experiment with gels/blots were processed in parallel, which needs to be stated in the legend.

Figures should be provided at approximately the size that they are to be printed at (single column is 86 mm, double column is 170 mm) and should not exceed an A4 page (8.5 x 11"). Reduction to the scale that will be used on the page is not necessary, but multi-panel figures should be sized so that the whole figure can be reduced by the same amount at the smallest size at which essential details in each panel are visible. In the interest of our colour-blind readers we ask that you avoid using red and green for contrast in figures. Replacing red with magenta and green with turquoise are two possible colour-safe alternatives. Lines with widths of less than 1 point should be avoided. Sans serif typefaces, such as Helvetica (preferred) or Arial should be used. All text that forms part of a figure should be rewritable and removable.

We accept files from the following graphics packages in either PC or Macintosh format:

- For line art, graphs, charts and schematics we prefer Adobe Illustrator (.AI), Encapsulated PostScript (.EPS) or Portable Document Format (.PDF). Files should be saved or exported as such directly from the application in which they were made, to allow us to restyle them according to our journal house style.

- We accept PowerPoint (.PPT) files if they are fully editable. However, please refrain from adding PowerPoint graphical effects to objects, as this results in them outputting poor quality raster art. Text used for PowerPoint figures should be

Helvetica (preferred) or Arial.

- We do not recommend using Adobe Photoshop for designing figures, but we can accept Photoshop generated (.PSD or .TIFF) files only if each element included in the figure (text, labels, pictures, graphs, arrows and scale bars) are on separate layers. All text should be editable in ‘type layers’ and line-art such as graphs and other simple schematics should be preserved and embedded within 'vector smart objects' - not flattened raster/bitmap graphics.

- Some programs can generate Postscript by 'printing to file' (found in the Print dialogue). If using an application not listed above, save the file in PostScript format or email our Art Editor, Allen Beattie for advice (a.beattie@nature.com).

Regardless of format, all figures must be vector graphic compatible files, not supplied in a flattened raster/bitmap graphics format, but should be fully editable, allowing us to highlight/copy/paste all text and move individual parts of the figures (i.e. arrows, lines, x and y axes, graphs, tick marks, scale bars etc.). The only parts of the figure that should be in pixel raster/bitmap format are photographic images or 3D rendered graphics/complex technical illustrations.

All placed images (i.e. a photo incorporated into a figure) should be on a separate layer and independent from any superimposed scale bars or text. Individual photographic images must be a minimum of 300+ DPI (at actual size) or kept constant from the original picture acquisition and not decreased in resolution post image acquisition. All colour artwork should be RGB format.

FIGURE LEGENDS – must not exceed 350 words for each figure to allow fit on a single printed NCB page together with the figure. They must include a brief title for the whole figure, and short descriptions of each panel with definitions of the symbols used, but without detailing methodology.

TABLES – main tables should be provided as individual Word files, together with a brief title and legend. For supplementary tables see below.

SUPPLEMENTARY INFORMATION – Supplementary information is material directly relevant to the conclusion of a paper, but which cannot be included in the printed version in order to keep the manuscript concise and accessible to the general reader. Supplementary information is an integral part of a Nature Cell Biology publication and should be prepared and presented with as much care as the main display item, but it must not include non-essential data or text, which may be removed at the editor's discretion. All supplementary material is fully peer-reviewed and published online as part of the HTML version of the manuscript. Supplementary Figures and Supplementary Notes are appended at the end of the main PDF of the published manuscript.

Supplementary items should relate to a main text figure, wherever possible, and should be mentioned sequentially in the main manuscript, designated as Supplementary Figure, Table, Video, or Note, and numbered continuously (e.g. Supplementary Figure 1, Supplementary Figure 2, Supplementary Table 1, Supplementary Table 2 etc.).

Unprocessed scans of all key data generated through electrophoretic separation techniques need to be presented in a supplementary figure that should be labelled and numbered as the final supplementary figure, and should be mentioned in every relevant figure legend. This figure does not count towards the total number of figures and is the only figure that can be displayed over multiple pages, but should be provided as a single file, in PDF or TIFF format. Data in this figure can be displayed in a relatively informal style, but size markers and the figures panels corresponding to the presented data must be indicated.

The total number of Supplementary Figures (not including the “unprocessed scans” Supplementary Figure) should not exceed the number of main display items (figures and/or tables (see our Guide to Authors and March 2012 editorial http://www.nature.com/ncb/authors/submit/index.html#suppinfo; http://www.nature.com/ncb/journal/v14/n3/index.html#ed). No restrictions apply to Supplementary Tables or Videos, but we advise authors to be selective in including supplemental data.

Each Supplementary Figure should be provided as a single page and as an individual file in one of our accepted figure formats and should be presented according to our figure guidelines (see above). Supplementary Tables should be provided as individual Excel files. Supplementary Videos should be provided as .avi or .mov files up to 50 MB in size. Supplementary Figures, Tables and Videos much be accompanied by a separate Word document including titles and legends.

GUIDELINES FOR EXPERIMENTAL AND STATISTICAL REPORTING

REPORTING REQUIREMENTS – We are trying to improve the quality of methods and statistics reporting in our papers. To that end, we are now asking authors to complete a reporting summary that collects information on experimental design and reagents. The Reporting Summary can be found here https://www.nature.com/documents/nr-reporting-summary.pdf)If you would like to reference the guidance text as you complete the template, please access these flattened versions at http://www.nature.com/authors/policies/availability.html.

STATISTICS – Wherever statistics have been derived the legend needs to provide the n number (i.e. the sample size used to derive statistics) as a precise value (not a range), and define what this value represents. Error bars need to be defined in the legends (e.g. SD, SEM) together with a measure of centre (e.g. mean, median). Box plots need to be defined in terms of minima, maxima, centre, and percentiles. Ranges are more appropriate than standard errors for small data sets. Wherever statistical significance has been derived, precise p values need to be provided and the statistical test used needs to be stated in the legend. Statistics such as error bars must not be derived from n<3. For sample sizes of n<5 please plot the individual data points rather than providing bar graphs. Deriving statistics from technical replicate samples, rather than biological replicates is strongly discouraged. Wherever statistical significance has been derived, precise p values need to be provided and the statistical test stated in the legend.

Information on how many times each experiment was repeated independently with similar results needs to be provided in the legends and/or Methods for all experiments, and in particular wherever representative experiments are shown.

We strongly recommend the presentation of source data for graphical and statistical analyses as a separate Supplementary Table, and request that source data for all independent repeats are provided when representative experiments of multiple independent repeats, or averages of two independent experiments are presented. This supplementary table should be in Excel format, with data for different figures provided as different sheets within a single Excel file. It should be labelled and numbered as one of the supplementary tables, titled “Statistics Source Data”, and mentioned in all relevant figure legends.

--------- Please don't hesitate to contact NCB@nature.com should you have queries about any of the above requirements ---------

Version 1:

Decision Letter:

Our ref: NCB-A52141A

22nd August 2024

Dear Dr. Cesare,

Thank you for submitting your revised manuscript "Homologous recombination promotes mitotic death to suppress interferon production following genome damage" (NCB-A52141A). It has now been seen by the original referees and their comments are below. The reviewers find that the conclusions were strengthened in revision, and therefore we'll be happy in principle to publish it in Nature Cell Biology, pending minor revisions to satisfy the referees' final requests in the text of the manuscript and to comply with our editorial and formatting guidelines.

Please note that the current version of your manuscript is in a PDF format. Could you please email us a copy of the file in an editable format (Microsoft Word or LaTex) as we can not proceed with PDFs at this stage? Thank you in advance for your attention to this point.

With the Word file in-hand, we will be performing detailed checks on your paper and will send you a checklist detailing our editorial and formatting requirements in about 1-2 weeks. Please do not upload the final materials and make any revisions until you receive this additional information from us.

Thank you again for your interest in Nature Cell Biology. Please do not hesitate to contact me if you have any questions.

Sincerely,

Melina

Melina Casadio, PhD
Senior Editor, Nature Cell Biology
ORCID ID: https://orcid.org/0000-0003-2389-2243

Reviewer #1 (Remarks to the Author):

The authors have done a substantial amount of work to strengthen the conclusions that HR intermediates trigger mitotic cell death after IR. The exact mechanism can be a subject for future study. This is important work that links DNA repair mechanism to inflammatory signaling.

Reviewer #2 (Remarks to the Author):

In their revised manuscript entitled ‘Homologous recombination promotes mitotic death to suppress interferon production following genome damage’, Szmyd et al. show that specific DNA repair pathways can impact on whether a cell encountering

DNA damage will arrest in a subsequent mitosis, or not. In turn, this decision can impact on the strength of cGAS- and MAVS-dependent innate immune signalling. Overall, the authors have satisfied all my essential concerns, and have put together a highly compelling study that should have considerable impact on many fields relating to genome instability.

Whilst not essential, I highly recommend two minor points to be considered by the authors.
1. They should mention that mitotic arrest and cell death after DNA damage were already reported by (at least) three studies, including one of their own: PMIDs 24080495, 31530811, 32877684)
2. I'm not entirely sure that they have excluded that WAPL may act through the spindle checkpoint. The experiment the authors have added (Ext data Fig. 7l) used a very high amount of nocodazole, which may generate a stronger SAC stimulus than DNA damage by-products. Whilst it's fine if this remains as is for this manuscript, ideally they would use lower nocodazole concentrations, which can generate weaker SAC stimuli.

Reviewer #3 (Remarks to the Author):

My concerns have been met. I would still remove the reference to mitotic catastrophe at the end of the intro, as also a second reviewer is not really happy with this terminology (line 88), maybe "different modes of cell death" or cell death signalling events...

Version 2:

Decision Letter:

Dear Dr Cesare,

I am pleased to inform you that your manuscript, "Homologous recombination promotes non-immunogenic mitotic cell death upon DNA damage", has now been accepted for publication in Nature Cell Biology.

Thank you for sending us the final manuscript files to be processed for print and online production, and for returning the manuscript checklists and other forms. Your manuscript will now be passed to our production team who will be in contact with you if there are any questions with the production quality of supplied figures and text.

Over the next few weeks, your paper will be copyedited to ensure that it conforms to Nature Cell Biology style. Once your paper is typeset, you will receive an email with a link to choose the appropriate publishing options for your paper and our Author Services team will be in touch regarding any additional information that may be required.

After the grant of rights is completed, you will receive a link to your electronic proof via email with a request to make any corrections within 48 hours. If, when you receive your proof, you cannot meet this deadline, please inform us at rjsproduction@springernature.com immediately.

You will not receive your proofs until the publishing agreement has been received through our system.

Due to the importance of these deadlines, we ask that you please let us know now whether you will be difficult to contact over the next month. If this is the case, we ask you provide us with the contact information (email, phone and fax) of someone who will be able to check the proofs on your behalf, and who will be available to address any last-minute problems.

If you have any questions about our publishing options, costs, Open Access requirements, or our legal forms, please contact ASJournals@springernature.com

Once your paper has been scheduled for online publication, the Nature press office will be in touch to confirm the details. An online order form for reprints of your paper is available at https://www.nature.com/reprints/author-reprints.html. All co-authors, authors' institutions and authors' funding agencies can order reprints using the form appropriate to their geographical region.

Publication is conditional on the manuscript not being published elsewhere and on there being no announcement of this work to any media outlet until the online publication date in Nature Cell Biology.

You may wish to make your media relations office aware of your accepted publication, in case they consider it appropriate to organize some internal or external publicity. Once your paper has been scheduled you will receive an email confirming the publication details. This is normally 3-4 working days in advance of publication. If you need additional notice of the date and time of publication, please let the production team know when you receive the proof of your article to ensure there is sufficient time to coordinate. Further information on our embargo policies can be found here:
https://www.nature.com/authors/policies/embargo.html

Please note that *Nature Cell Biology* is a Transformative Journal (TJ). Authors may publish their research with us through the traditional subscription access route or make their paper immediately open access through payment of an article-processing charge (APC). Authors will not be required to make a final decision about access to their article until it has been accepted. Find out more about Transformative Journals

Authors may need to take specific actions to achieve compliance with funder and institutional open access mandates. If your research is supported by a funder that requires immediate open access (e.g. according to Plan S principles) then you should select the gold OA route, and we will direct you to the compliant route where possible. For authors selecting the subscription publication route, the journal's standard licensing terms will need to be accepted, including self-archiving policies. Those licensing terms will supersede any other terms that the author or any third party may assert apply to any version of the manuscript.

To assist our authors in disseminating their research to the broader community, our SharedIt initiative provides you with a unique shareable link that will allow anyone (with or without a subscription) to read the published article. Recipients of the link with a subscription will also be able to download and print the PDF.

If your paper includes color figures, please be aware that in order to help cover some of the additional cost of four-color reproduction, Nature Portfolio charges our authors a fee for the printing of their color figures. Please contact our offices for exact pricing and details.

As soon as your article is published, you will receive an automated email with your shareable link.

If you have not already done so, we strongly recommend that you upload the step-by-step protocols used in this manuscript to protocols.io (https://protocols.io), an open online resource that allows researchers to share their detailed experimental know-how. All uploaded protocols are made freely available and are assigned DOIs for ease of citation. Protocols and Nature Portfolio journal papers in which they are used can be linked to one another, and this link is clearly and prominently visible in the online versions of both. Authors who performed the specific experiments can act as primary authors for the Protocol as they will be best placed to share the methodology details, but the Corresponding Author of the present research paper should be included as one of the authors. By uploading your Protocols onto protocols.io, you are enabling researchers to more readily reproduce or adapt the methodology you use, as well as increasing the visibility of your protocols and papers. You can also establish a dedicated workspace to collect your lab Protocols. Further information can be found at https://www.protocols.io/help/publish-articles.

You can use a single sign-on for all your accounts, view the status of all your manuscript submissions and reviews, access usage statistics for your published articles and download a record of your refereeing activity for the Nature Portfolio.

Please feel free to contact us if you have any questions.

With kind regards,

Melina Casadio, PhD
Senior Editor, Nature Cell Biology
Consulting Editor, Nature Structural & Molecular Biology
ORCID ID: https://orcid.org/0000-0003-2389-2243

Click here if you would like to recommend Nature Cell Biology to your librarian
http://www.nature.com/subscriptions/recommend.html#forms

** Visit the Springer Nature Editorial and Publishing website at www.springernature.com/editorial-and-publishing-jobs for more information about our career opportunities. If you have any questions please click here.**

Open Access This Peer Review File is licensed under a Creative Commons Attribution 4.0 International License, which permits use, sharing, adaptation, distribution and reproduction in any medium or format, as long as you give appropriate credit to the original author(s) and the source, provide a link to the Creative Commons license, and indicate if changes were made.
In cases where reviewers are anonymous, credit should be given to 'Anonymous Referee' and the source.
The images or other third party material in this Peer Review File are included in the article's Creative Commons license, unless indicated otherwise in a credit line to the material. If material is not included in the article's Creative Commons license and your intended use is not permitted by statutory regulation or exceeds the permitted use, you will need to obtain permission directly from the copyright holder.
To view a copy of this license, visit https://creativecommons.org/licenses/by/4.0/

Point by point response to the reviewers' comments.

We sincerely express our gratitude to the reviewers for their thoughtful assessment of our work. Their comments were well received, and the resulting changes have improved manuscript quality.

Our responses to their comments/questions are in blue. The response to Reviewer 1 starts on Page 1, Reviewer 2 on Page 5, and Reviewer 3 on page 10.

Author list - As a result of her contribution during revision, Dr. Melanie Walter was added to the author list. This change was agreed upon by all authors.

Reviewer #1:

The authors present a detailed story that implicates DNA repair mechanism as a key determinant of mitotic cell death, which dictates pattern recognition of missegregated chromosomal DNA. Based on supportive data, the authors propose that HR driven double Holliday Junctions elicit mitotic cell death and this suppresses pattern recognition by cGAS and MAVs. Moreover, ATR and cohesion fatigue contributes to these responses. BRCA2, PALB2, and RAD51 knockdown in S/G2 irradiated cells prevents mitotic cell death and led to increased inflammatory signaling. Conversely, DNA-PK inhibition, or knockdown of Lig4, RAD52, or Pol theta increased it in a manner that depended on Rad51. Knockdown of SLX4 or RTEL1 is used as evidence for dHJs being the toxic lesion that causes mitotic cell death. Cohesion fatigue due to prolonged mitotic duration promoted mitotic cell death, while WAPL knockdown prevented mitotic cell death and increased inflammatory signaling.

This is an interesting study that brings a new perspective to the understand how genomic instability communicates with innate immune signaling. An intriguing concept put forth by the authors is that unresolved HR intermediates are the cause of mitotic cell death and suppress inflammatory signals. This data may help explain prior observations that NHEJ loss prevents cGAS-STING signaling following IR. They also invoke suppression of HR as a means by which ATRi increases inflammatory signaling. However, there are aspects of the model that are insufficiently supported at this point and examples in the literature that may conflict with the model. The authors should resolve these issues in a revised manuscript.

We thank the reviewer for their efforts and have addressed each point below.

Note: We numbered the reviewer comments and subdivided when a comment contained multiple questions/points.

Major points:

1. The authors show that Pol theta loss increases mitotic cell death and prevents inflammatory signaling. Pol theta inhibitors have been shown to increase cGAS-STING signaling. The authors should discuss this in light of their own data.

We have included reference to the relevant paper (Oh et al, JCI 2023, reference #69) and discussed the pertinent findings in the final paragraph of the discussion. The paper in question demonstrates that POLθ inhibition induces interferon signalling specifically in the context of BRCA2 deficiency. This is consistent with our findings that HR deficiency rescues mitotic death to promote inflammatory signalling.

2a. The data to support dHJs as critical to mitotic cell death and suppression of cGAS responses is insufficiently supported. The authors show that SLX4 and RTEL1 loss increases mitotic cell death and suppresses inflammatory signals. There are several ways this model could be better tested. BLM helicase, SLX4, and GEN1 are more involved in dissolving/resolving dHJs. Knockdown of these factors alone, and in combination should be performed rather than RTEL1.

Due to the pleiotropic functions of BLM, we elected to deplete RMI2, a key component of the BLM-TOP3A-RMI1/2 double Holliday Junction (dHJ) dissolution complex. Like RTEL1 and SLX4 depletion, RMI2 depletion induced a significant increase in immediate mitotic death in 2 Gy irradiated G2 cells (Fig. 3I and Extended Data Fig. 5D). Combinatorial RMI2 and SLX4 depletion enhanced this effect consistent with our interpretation that unresolved dHJs promote mitotic cell death (Extended Data Fig. 5D).

We also depleted GEN1 but found no effect on 2 Gy irradiated G2 cultures (Extended Data Fig. 5D). We note BTR and SLX4 resolve dHJs in interphase or at the G2/M transition, while GEN1 is mitosis specific. We

interpret these data to indicate that mitotic arrest is propagated by recombination intermediates that are passed into mitosis, prior to GEN1-dependent resolution.

We discuss the above in the relevant results and discussion sections.

2b. In addition, expression of the bacterial HJ resolvase RuvC could be used to see if this suppresses the response. RUVC has been expressed in human cells and can resolve HJ intermediates PMID: 34776211.

We tried exhaustively to complete this experiment but encountered technical limitations. We generated a nuclear localization signal (NLS) and fluorescently tagged RuvC (RuvC-NLS-miRFP670) but could only detect nuclear localized far-red signal in a minor subset of cells. Further, the NLS-miRFP670 signal waned quickly and before mitotic entry. We note control cells overexpressing mCherry-NLS from the same backbone-maintained expression throughout the imaging, and in prior efforts we routinely used miRFP670 without photo-bleaching (PMID: 33257806). Exogenous RuvC-NLS-miRFP670 expression did not impact mitotic outcomes following IR. However, this is likely a result of inefficient protein expression and/or degradation during the experiment. In agreement, we failed to secure any evidence that RuvC-NLS-miRFP670 impacted HR dynamics. Unfortunately, this was a failed experiment.

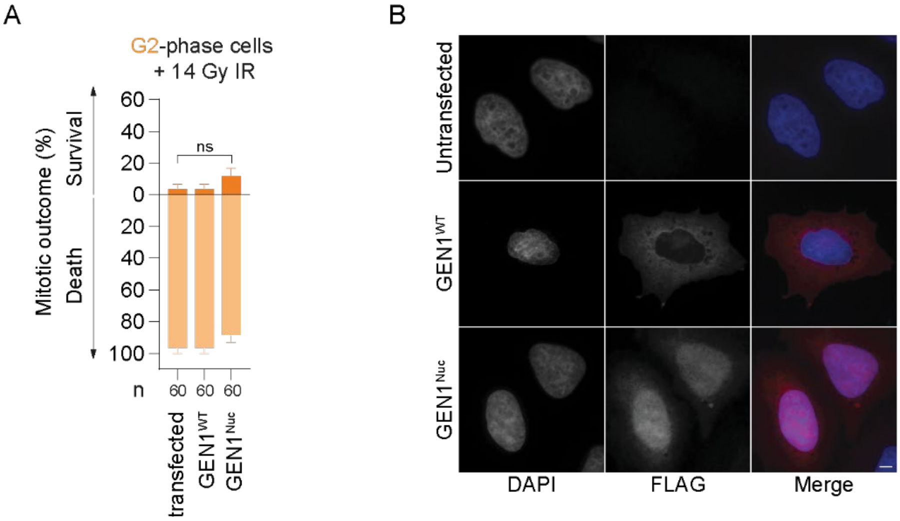

Response Fig. 1. **A)** First mitosis outcome measured by live imaging following 14 Gy IR of G2 enriched 3F HeLa cells transfected with the indicated constructs. Mean ± SEM of 2 biological replicates; Fisher's exact test. **C)** Immunofluorescence of HeLa cultures 30 hours post-transfection with the indicated constructs.

We attempted a similar experiment using an NLS tagged GEN1. GEN1 is excluded from interphase nuclei and gains access to chromatin in mitosis following nuclear envelope breakdown. We transfected HeLa with Wild Type (WT) or NLS-tagged GEN1, enriched for G2, 14 Gy irradiated, and observed mitotic outcomes. NLS-GEN1 induced a non-significant rescue of immediate mitotic death (Response Fig. 1A) However, only 30% of transfected cells expressed WT- or NLS-GEN1 as indicated by immunofluorescence (Response Fig. 1B). The subdued mitotic death rescue was likely a result of NLS-GEN1 expression in only a minor cell population. We attempted to create stable cell lines, but continuously failed to detect WT- or NLS-GEN1 expression following antibiotic selection. We anticipate exogenous GEN1 is toxic (it is also possible that RuvC-NLS-miRFP670 was toxic). While the NLS-GEN1 experiment initially looked promising, this too was a failed experiment as we could never maintain a cell population with stable NLS-GEN1 expression. We hope the reviewer appreciates the biological limitations encountered.

We note the data from 2 Gy irradiated cells depleted for RTEL1, SLX4, and/or RMI2 ± BRCA2/PALB2 siRNA strongly suggest that unresolved HR intermediates precipitate subsequent mitotic arrest and death.

3. An alternative explanation to several findings is that cells without ligase 4 have reduced mitotic progression due to excessive damage that results in prolonged G2 arrest. In addition, that ATR inhibition increases inflammatory signalling by premature entry into mitosis and chromosome missegregation rather than suppression of HR. The authors should delineate whether some of their results can be explained by this rather than by mitotic cell death and include discussion in the text.

[REDACTED]

4. MPS1 inhibitors increase chromosome missegregation and inflammatory signals. Do mitotic checkpoint errors bypass this proposed dHJ mitotic cell death checkpoint mechanisms?

Yes, suppressing the spindle assembly (i.e. mitotic) checkpoint inhibits HR-dependent mitotic death. In response to all three reviewers, we have edited the text to better covey that HR-dependent mitotic cell death is a function of SAC-dependent mitotic arrest. We also show this through the following data.

- Inclusion of new multi-dimensional plots for 3F HeLa cultures treated with Reversine (the MPS1 inhibitor) ± 14 Gy, demonstrating complete suppression of mitotic death when the SAC is silenced (Fig. 1F).
- Inclusion of new data demonstrating that Reversine completely rescues mitotic death even under conditions of increased HR in 14 Gy irradiated and RAD52 depleted cells (Fig. 3G).
- Demonstration of a direct correlation between mitotic arrest and mitotic death.
  - Fig. 1D, 2C, 3C, 5E, 6C, 8E
  - Extended Data Fig. 1E, 3C, 3I, 4C, 4I, 5E-G, 9E
- New data in Extended Data Fig. 4E and 7I demonstrating that RAD51, BRCA2, PALB2, or WAPL depletion does not silence SAC activation by the microtubule poison Nocodazole. Conversely, Hesperadin, an inhibitor of the SAC component Aurora B Kinase, rescued mitotic death and shortened mitotic arrest in Nocodazole treated cells. This demonstrates that RAD51, BRCA2, and PALB2 regulate mitotic death through their HR functions and not through direct SAC regulation.

Together our data indicate that: i) HR acts upstream of the SAC, ii) SAC activity is required for mitotic arrest and mitotic death, and iii) inhibiting HR rescues mitotic death by preventing mitotic arrest.

Reviewer #2:

In their article entitled "Homologous recombination promotes mitotic death to suppress the innate immune response", Szmyd et al. present evidence that specific homologous recombination repair of DSBs can lead to mitotic arrest and subsequent cell death. In contrast, other repair pathways do not lead to this phenomenon. Because these repair pathways are cell cycle regulated, the timing of DSB formation and repair can impact the behaviour of cells during subsequent mitoses. This is an interesting and stimulating manuscript that has the potential to be an important contribution to our understanding of the cellular consequences of DNA damage. However, in its current version, the manuscript suffers somewhat from an over-reliance on siRNA, which makes especially the interpretation of double-knockdowns and effect sizes difficult. Furthermore, I think that a possible involvement of the length of mitotic arrest is currently not investigated sufficiently. Overall, the points outlined below would need to be addressed before publication.

We thank the reviewer for their efforts and have addressed each point below.

Note: We numbered the reviewer comments and subdivided when a comment contained multiple questions/points.

Major points:

1a. A major working model of mitotic arrest is that the slow degradation of Cyclin B competes with a build-up of apoptotic activity for determining cell fate as long as the spindle checkpoint cannot be satisfied (PMID 18656424). Therefore, mitotic cell death frequently is a function of time. A stronger spindle checkpoint stimulus will keep cells arrested for longer, and therefore potentially lead to a higher fraction of cells dying in mitosis. It would therefore be interesting to establish how mitotic arrest duration is affected by the authors' treatments. For many experiments, this information is provided, but for many experiments it is not (for example Fig 1G, 2B, 2E, etc.), and for some experiments (like Fig. 3C), it is not clear if the timing plots are done for all cells or deconvolved for radiation within a specific cell cycle stage.

The reviewer is correct RE: mitotic arrest and death. To address this comment, we added several experiments/analyses that directly address this point (listed below). Collectively our data indicate that HR-dependent immediate mitotic death is executed through intrinsic apoptosis, as a function of persistent and SAC-dependent mitotic arrest. We have edited the text throughout to better convey this message and the specifics of the underlying data presented in each plot.

New analyses/experiments.

- Inclusion of new multi-dimensional plots for 3F HeLa cultures treated with Reversine (the MPS1 inhibitor) ± 14 Gy, demonstrating complete suppression of mitotic death when the SAC is silenced (Fig. 1F).
- Inclusion of new data demonstrating that Reversine completely rescues mitotic death even under conditions of increased HR in 14 Gy irradiated and RAD52 depleted cells (Fig. 3G).
- Demonstration of a direct correlation between mitotic arrest and mitotic death.
    - Fig. 1D, 2C, 3C, 5E, 6C, 8E
    - Extended Data Fig. 1E, 3C, 3I, 4C, 4I, 5E-G, 9E
- Demonstrate of intrinsic apoptotic signalling as a function of mitotic duration in G2 irradiated cells (Fig. 7G; see Rev 3, point 2)

1b. A further reason for doing this is that some DNA damage response factors like CHK2, ATR, BRCA1, BRCA2 are thought to potentially have functions in activating/counteracting the SAC (see for example PMID 20364141, 22340495, 29170278). Furthermore, specific repair defects could have varying impact on the strength of the SAC. For example, it looks from the analysis of Fig. 3C that the increased survival of siRAD51, BRCA2, PALB2 might primarily come from a subset of cells that do not arrest anymore. Timing is also relevant for Fig. 5 - perhaps the more severe chromosome alignment defects correlate with or cause a longer mitotic arrest, thereby allowing for more time to die. It would be great if this could systematically be assessed, as it may impact on the conclusions the authors would draw/the working model they can derive from their experiments. Importantly, I believe that the outcome of these experiments wouldn't impact the importance of the overall conclusions the authors make.

We addressed this insightful suggestion as follows.

First - We directly examined potential direct SAC regulation by BRCA2, PALB2, RAD51, or WAPL independent of IR. We treated cells with 3.3μM Nocodazole, a concentration that completely inhibits microtubule polymerization, resulting in permanent SAC activation (PMID: 21407176). We then assayed mitotic duration and outcome through live imaging. Hesperadin, an inhibitor of the SAC regulator Aurora B Kinase, was

used as a positive control. These experiments were done in the absence of IR. As expected, Hesperadin suppressed mitotic arrest and promoted mitotic survival. Neither RAD51, BRCA2, PALB2, nor WAPL depletion influenced Nocodazole-induced mitotic arrest or mitotic death. Therefore, none of the examined HR factors, nor WAPL, are direct SAC regulators. These new data are included as Extended Data Fig. 4E and 7I and discussed in the text.

Second – we added a new panel (Fig. 6C) that demonstrates the relationship between chromosome alignment, mitotic duration, and mitotic outcome. This analysis demonstrates that mitotic death correlates with severe chromosome misalignment and significant mitotic arrest, while mitotic survival correlates with mild to moderate chromosome alignment and normal mitotic duration.

The reviewer is correct that throughout the paper, mitotic death rescue corresponds with a reduction in the percentage of cells that experiencing mitotic arrest. Our experiments targeting dHJ resolution pathways (Fig. 3I and Extended Data Fig. 5C-F) suggest that SAC activation following IR results from passage of HR intermediates into mitosis. We interpret the findings to indicate that suppressing HR engagement prevents the HR intermediates that cause subsequent SAC-dependent mitotic arrest. We elaborate on this concept in the manuscript text.

2. There is somewhat of an over-reliance on siRNA. In general, I don't think every single experiment needs to be repeated with CRISPR, but especially for double mutants, this should be done. For example, when discussing the results of Fig. 4E-H/S4G, the authors make conclusions about Rad51 and WAPL having "independent mechanisms of mitotic death rescue", based on siRNA-mediated epistasis analysis. However, the combinatorial effects of incomplete depletion of two proteins can be very hard to predict, and it is possible that what they observe are additive effects of partial depletion of two proteins that are in the same pathway.

We appreciate the reviewer's comment and generated WAPL knockout (KO) 3F HT1080 6TG cells using CRIPSR (Extended Data Fig. 7D). We also treated 3F HT1080 WAPL KO cells ± PALB2 or RAD51 siRNA. All results from WAPL KO and siRNA were consistent. Specifically:

- WAPL KO suppressed cohesion fatigue (Extended Data Fig. 7E)
- WAPL KO + BRCA2 siRNA improve mitotic survival compared to BRCA2 or WAPL alone (Extended Data Fig. 7F)
- WAPL KO promoted survival by suppressing cohesion fatigue, while RAD51 enabled survival despite cohesion fatigue (Extended Data Fig. 7E, F)
- WAPL and BRCA2 co-depletion in 3F HT1080 6TG phenocopied WAPL KO + BRCA2 siRNA (Extended Data Fig. 7D, F)

The data collectively indicate that WAPL and HR promote mitotic death via independent mechanisms that are universal across different cell lines. These findings are discussed in the text.

We spent considerable effort attempting to create PALB2 or BRCA2 knockouts in 3F HeLa and 3F HT1080 6TG. Using different approaches and gRNAs, we routinely isolated clones that harboured truncation mutants that retained some level of HR functionality as measured by RAD51 foci formation. We observed some mitotic death rescue in BRCA2 or PALB2 cells with diminished but not absent RAD51 foci. However, due to the uncharacterized nature of the truncation mutants, we elected to exclude these data from the manuscript. We note BRCA2 and PALB2 are essential for development, as is RAD51. We anticipate BRCA2 or PALB deletion in the cell lines examined may be lethal due to the role these factors play in replication stress mitigation.

For the epistatic experiments investigating cell death, we employed CRISPR knockouts of BAX and BAK.

3. Whilst not entirely likely (at least not for all factors), there is a possibility that at least some of the DNA repair factors can carry out DNA repair functions during mitosis, and exert their critical function therefore during mitosis, and not the preceding interphase. This is not addressed by the siRNA experiments. Where available, chemical inhibitors could be used to address this (ie, according to the model, addition in mitosis should not give a phenotype, but addition during the relevant cell cycle stage should mimic loss throughout the cell cycle).

To address this comment, we performed live imaging experiments where NHEJ was inhibited with 0.5μM of the DNA-PKcs inhibitor NU7441, or HR with 50μM of the RAD51 inhibitor RI(dI)-2.

For the NHEJ experiment, we added NU7441 or DMSO vehicle one hour before treating with 14 Gy IR. 24 hours post-IR (a time before cells enter mitosis), we exchanged the media with fresh media containing NU7441 or DMSO. Live imaging revealed that NHEJ inhibition at the time of IR, but not 24 hours later, promoted mitotic death in the G1 irradiated cells. Thus, NHEJ function at DSB induction, but not later in interphase or mitosis, was critical for survival of G1 irradiated cells. These data are presented in Extended Data Fig. 2F, G and discussed in the corresponding text.

The RAD51 inhibitor 50µM RI(dI)-2 suppressed HR with minimal toxicity. For this experiment cells were G1/S synchronized, released, and treated as follows: 5 hours post-release, DMSO or RI(dI)-2 were added to the culture media; 6 hours post-release, the cells were 14 Gy irradiated while in G2; 18 hours post-IR/24 hours posts-release, the media was exchanged with fresh media containing either DMSO or RI(dI)-2. Cells typically entered mitosis within four hours of media exchange. Adding RI(dI)-2 1-hour prior to IR and washing out at 18 hours later, or adding RI(dI)-2 18 hours post-IR rescued mitotic death to the same extent. Maintaining RI(dI)-2 from before IR throughout the remainder of the experiment conferred the greatest mitotic death rescue. If mitotic HR was responsible for mitotic lethality, adding RI(dI)-2 at 18 hrs post IR or should rescue mitotic death to the same extent as maintaining RI(dI)-2 in the media throughout the experiment. We did not observe this outcome. Our finding that maintaining RI(dI)-2 in the media from damage through mitosis conferred the greatest mitotic death rescue, suggests that HR engagement at any point until mitotic entry can promote mitotic death. These data are presented in Extended Data Fig. 3F-I and discussed in the corresponding text.

4. I am slightly confused about the chromosome aberration data (Fig. 4/S4). Are radials/chromatid fusions primarily made by S/G2 repair and therefore HR, and are multi-centric/circular chromosomes primarily made in G1 by NHEJ? If so, this should be explained and referenced (and ideally also shown by the authors own experiments by irradiating G1 vs S/G2 cells).

Radials/chromatid-type fusions are associated with post-replicative S/G2 repair, while NHEJ mediated G1 repair is associated with formation of multi-centric chromosomes. This readout is commonly employed in telomere protection experiments (PMID: 28959974). To determine if the same occurs following IR, we provided new data related to the cell cycle-dependency of different chromosomal rearrangements following 14 Gy IR. We prepared cytogenetic chromosome spreads from synchronized HeLa cultures irradiated with 14 Gy in G1, S, or G2. This was consistent with radial chromosomes and chromatid-type fusions being predominantly formed by S/G2 repair (Fig. 4B), and multi-centric and circular chromosomes formed through G1 repair (Fig. 4C).

5a. The data on cohesion fatigue: It looks like siRAD51 and control have similar amounts of cohesion defects (Fig. 4F – now figure 5B), but there are differences in mitotic arrest duration/survival. Does that not mean that cohesion defects are unlikely to be the cause of mitotic arrest/survival differences?

Cohesion fatigue results from microtubule pulling forces exerted during prolonged mitosis, indicating that cohesion fatigue is a consequence, not a cause, of mitotic arrest. We have added new data which demonstrate this point and clarified in the text (new Extended Data Fig. 7B).

The reviewer is correct that in 14 Gy irradiated cells, control and RAD51 siRNA confer similar levels of cohesion fatigue. Our characterization of severe chromosome misalignment (Fig. 6A) includes spontaneous loss of metaphase plate integrity during mitotic arrest: an outcome consistent with cohesion fatigue (PMID: 31530811). New analysis included in the manuscript demonstrates that severe chromosome misalignment in cells with functional HR strongly correlates with mitotic arrest and death (Fig. 6C). This is indicative of cohesion fatigue preceding mitotic death when HR is functional.

Cohesion fatigue, chromosome alignment defects, and mitotic death are all rescued by WAPL; suggesting that cohesion fatigue precipitates mitotic lethality. HR deficiency, however, enables mitotic escape despite cohesion fatigue through a yet undefined mechanism. The additive effects on mitotic survival of targeting WAPL and HR suggests independent pathways of mitotic death induction; one of which we interpret to be triggered by cohesion fatigue.

5b. It seems to me that the simplest hypothesis to explain the impact of WAPL loss is that it impacts on the strength of the spindle checkpoint (either by leading to the generation of different DNA repair structures that do not generate as strong a SAC signal, or by directly impacting the SAC). Indeed, the data from Fig. 4H

indicate that a substantial fraction of cells depleted for WAPL don't arrest in mitosis at all following irradiation). This should be discussed at the very least.

This astute observation prompted inclusion of a new control experiment as described in the response to Point 1B. WAPL depleted cells still arrested and died following treatment with the spindle poison Nocodazole, though we note WAPL depleted cells did perish more quickly during mitotic arrest (Extended Data Fig 7I). WAPL therefore does not directly influence the SAC activation or maintenance. In agreement, irradiated WAPL depleted cells that die in mitosis do so with the same duration as siRNA controls (i.e. WAPL depletion also did not impact the SAC in irradiated cells)(Fig 5E).

We expect that reinforcing cohesion between sister chromatids in WAPL KO/siRNA promote DNA repair outcomes that prevent SAC activation in the first place, as mentioned in the discussion.

5c. In addition, some information seems to be missing – for example it does not seem to be indicated when the snapshot of Fig. 4F (now Fig 5B) was taken.

This is now clarified in the figure legend and methods. The data in Fig. 5B was taken at 36 hours post-IR for control and WAPL knockdown cells, and 24 hours post-IR for RAD51 and RAD51/WAPL siRNA. The different timepoints were required because RAD51 depletion accelerated mitotic entry of S/G2 irradiated cells (Extended Data Fig. 7H; discussed in Rev 1 point 3 and Rev 2 point 6b). At the time points analysed, the examined mitotic cells had spent an equivalent time, on average, in cell division.

5d. Similarly, the kinetics of cohesion fatigue are not shown. Personally, I would suggest removing the cohesion fatigue aspect from the manuscript, as it seems to dilute the main point of the study.

We provided new data demonstrating the kinetics of cohesion fatigue following IR. This shows cohesion fatigue is function of mitotic arrest duration (Extended Data Fig. 7B).

We appreciate the suggestion to remove the cohesion fatigue data. We elected to leave it in because, in our opinion, these data explain how chromosome segregation errors in HR depleted cells lead to a strong interferon response through aberrant cytokinesis. Further, it demonstrates a mode of resistance to mitotic death and the influence this has in interferon signalling; findings with potential clinical implications.

6a. The model from Fig. 7E is a bit confusing. Does it suggest that there is a subset of DSB repair products that cause mitotic arrest? Or is the subset of DSBs whose repair isn't finished by the time cells enter mitosis critical?

We thank the reviewer for pointing out the confusion. Our data are consistent with the latter: mitotic arrest is caused by a subset of DSBs whose repair isn't finished by the time cells enter mitosis. Specifically, the data suggest double Holliday Junctions (dHJs) from unfinished HR are the culprit that promote SAC activation. This is highlighted by the induction of mitotic death with low dose IR when RMI2, SLX4, or RTEL1 are depleted (Fig. 3I and Extended Data Fig. 5C, D). Depleting these factors promotes dHJ formation (RTEL1) or prevents dHJ resolution (RMI2, SLX4). We can suppress mitotic death in RTEL1 or SLX4 depleted cells by preventing upstream strand-invasion and HR initiation. This indicates that mitotic arrest results from a process where HR begins but is not finalized. We anticipate passage of dHJs into mitosis promotes SAC-dependent arrest and the resulting mitotic lethality. We acknowledge that at present it is unclear how dHJs promote mitotic arrest. We offer our working hypothesis in the discussion.

6b. If the latter, can mitotic lethality be rescued by extending G2? This can be tested by addition of RO3306 to cells for various periods of time before washout and release into mitosis. (Note that this experiment might require careful addition of RO3306 specifically after S phase, as previous work (PMID 35443178) indicated that RO3306 can cause replication stress in certain contexts).

[REDACTED]

Minor points:

1. The usage of the term "mitotic catastrophe". This is a problematic term, as it can refer to many different things. For example, Vitale et al. (PMID 21527953) say "…'mitotic catastrophe' has been widely used to describe a form of cell death affecting higher eukaryotes, and several attempts have been made to precisely define mitotic catastrophe. For instance, mitotic catastrophe has been delineated as a type of cell death that: results from abnormal mitosis and is associated with spontaneous premature chromosome condensation and the formation of large cells with multiple micronuclei; derives from premature or inappropriate

entry of cells into mitosis; or occurs either during or shortly after a dysregulated or failed mitosis.”. Because this is such an imprecise term, and refers to so many discrete phenomena, I would therefore prefer it if the authors used more precise terms like “mitotic cell death” and “delayed interphase cell death” (or similar) for the bulk of the manuscript. (although it is obviously fine to discuss “mitotic catastrophe” in the introduction and discussion, and where necessary in the results).

We concur. The text has been edited throughout as suggested.

2. The Rad51 foci number plots (like Fig. 3E) are often so small that is hard to see the relevant data.

We have changed the size of these panels to make them more discernible.

3. To interpret the mitotic death experiments with the combination of siRAD52 and VE-822 addition would require the controls from Fig. S3B/C (now Extended Data Fig 4F, G) to also be carried out with a combination treatment.

Fig. 4F, G are I-SceI reporter assays. Here an I-SceI restriction site is located within a genetic cassette, that when repaired in a specific manner, results in GFP production. The indicated panels simply show the impact of ATR inhibition on HR or SSA repair using the appropriate corresponding published reporters. We are not measuring cell death in this specific assay and only mean to show that inhibiting ATR has a greater effect on HR than SSA.

4. I’m slightly confused about Fig. S6F – it looks like loss of MAVS, cGAS, IRF3 gets rid of the first peak of apoptosis, which I understand represents mitotic death in the first cell cycle after IR, but then in Fig. 6J, it looks like death is not impacted in the first mitosis.

While the IncuCyte experiments are a useful measure of relative apoptotic induction, they lack the resolution (spatially and temporally) of the DIC + fluorescent imaging. The higher resolution method shows no impact on the first cell cycle outcomes in irradiated cultures depleted for cGAS, MAVS, or IRF3 (Fig. 6K and Extended Data Fig. 9L).

5. When discussing Fig 6I/J, the authors refer to a possible discrepancy to PMID 31299200. The authors should point out that that study used a different means of causing mitotic arrest (spindle poisons, APC inhibitor) in contrast to DNA damage, and different cell line models. This could be the underlying reasons for the apparent discrepancy.

We agree and have edited the text accordingly to clarify.

6. There is a typo in line 1095.

Corrected.

Reviewer #3:

In their study, Szmyd and colleague establish a correlation between different DNA repair pathways activated in response to irradiation damage, cell cycle status and the propensity of p53-deficient model cancer cells (HeLa) to die either in the next mitosis, along the intrinsic mitochondrial pathway (BAX/BAK mediated), or later in the next G1 phase, after experiencing mitotic defects and slippage triggering CIN, by BAX/BAK independent mechanisms involving caspase-8 (presumably extrinsic apoptosis). The latter phenomenon coincides with increased mRNA expression of inflammatory genes prior cell death, induced by the asymmetric distribution of DNA content and cytosolic nucleic acid sensing (presumably due to micronuclei formation and their breakdown, and/or the accumulation of extra centrosomes). The authors conclude that the DNA-repair machineries dictate cell fate and that a combination of radiotherapy with certain inhibitors of DNA repair pathways may improve response rates (due to the activation of innate immunity pathways) and that the response of cancer patients that show certain DNA repair deficiencies can be better guided based on this new knowledge.

By live-cell imaging of HeLa cells expressing the three factor FUCCI system, allowing the discrimination of cells in S, G2/M and G1 phase, in combination with different strategies to perturb various DNA repair pathways, the authors provide strong evidence that the homology repair (HR) machinery promotes mitotic cell death in cells that experience double strand breaks while in S or G2 phase, while Inhibition of the HR pathway allows cell survival along with escape to the next G1 phase and activation of inflammatory target genes, prior to delayed cell death. In contrast, error prone repair pathways, such as non-homologous end joining (NHEJ) or single strand annealing (SSA), allow cells that experience DSBs in G1 to pass through mitosis, at the expense of increasing rates of DNA segregation errors, promoting inflammation. Suppressing these error prone repair pathways and favouring activation of HR components, promotes mitotic cell death, while inhibiting HR pathways favours escape from mitotic arrest and inflammatory gene expression, along with delayed cell death.

Together these experiments establish a highly interesting interrelation between mitotic and “postmitotic” cell death, the cell cycle phase DSBs are experienced, and the respective repair machineries that are subsequently activated. As pointed out in their discussion, these observations may have clinical implications, pending such interdependencies are also operative in cancerous tissues of patients.

I believe the study is technically sound and the experiments are well-designed and (mostly) elegantly controlled. My main critique arises from the very DNA repair-centric view of things and the conclusions drawn, that are not always substantiated by the data shown. The claims made are simply too bold, starting with the title and continuing throughout the manuscript. This is simply misleading and actually not needed, as the data is very interesting.

We sincerely thank the reviewer for their efforts which improved paper quality. Guided by the reviewer’s feedback we have changed the title and softened claims throughout.

Note: We numbered the reviewer comments and subdivided when a comment contained multiple questions/points.

Major points:

1. There is no mechanistic insight, how HR (or inhibition of NHEJ) would “promote” mitotic death, nor how non-HR repair pathways would suppress it. I rather believe that many of the phenomena observed might be explained by the time cells spend in mitosis rather than DNA repair pathways “dictating” if cells die or not, and how. Direct connections between DNA repair genes and the cell death machinery are not established in any of the paradigms tested, which is a clear shortcoming of an otherwise very elegant study.

We agree with the reviewer that mitotic death is a function of mitotic arrest. Moreover, we agree this was not adequately described in the initial submission (we note a similar comment by Reviewer #2, Major point 1a). To clarify, we have included several new data/analyses (listed below) and edited the text throughout. We now clearly describe that HR-dependent mitotic death is a function of SAC-dependent mitotic arrest/increased mitotic duration.

Our data suggest double Holliday Junctions (dHJs) from unfinished HR are the culprit that promote SAC-dependent mitotic arrest and subsequent mitotic death. This is highlighted by the induction of mitotic death with low dose IR when RMI2, SLX4, or RTEL1 are depleted (Fig. 3I, Extended Data Fig. 5C, D). Depleting these factors promotes dHJ formation (RTEL1) or prevents dHJ resolution (RMI2, SLX4). We can

suppress mitotic death in RTEL1 or SLX4 depleted cells by preventing upstream strand-invasion and HR initiation with PALB2 or BRCA2 depletion (Fig. 3I). These findings indicate that mitotic arrest results from a process where HR begins but is not finalized. We interpret these data to indicate that passage of dHJs from G2 into mitosis promotes SAC-dependent arrest, and the resulting mitotic lethality. We acknowledge that it remains unclear how dHJs maintain the SAC but offer a working hypothesis in the discussion. The data indicate that inhibiting SSA results in mitotic death following irradiation, because inhibiting SSA drives greater HR activity in response to IR-induced DSBs.

New data presented in Extended Data Fig. 4E and 7I Demonstrate that RAD51, BRCA2, PALB2, or WAPL depletion does not silence SAC activation by the microtubule poison Nocodazole. Conversely, Hesperadin, an inhibitor of the SAC component Aurora B Kinase, rescued mitotic death and shortened mitotic arrest in Nocodazole treated cells. This demonstrates that RAD51, BRCA2, and PALB2 regulate mitotic death through their HR functions and not through direct SAC regulation.

Together our data indicate that: i) Unresolved HR intermediates promote SAC activation, ii) SAC activity is required for mitotic arrest and mitotic death, iii) HR factors do not directly regulate the SAC, and iv) inhibiting HR rescues mitotic death by preventing mitotic arrest.

The new analyses/experiments directly showing the link between mitotic arrest and mitotic death are as follows and are addressed in the corresponding text.

- Inclusion of new multi-dimensional plots for 3F HeLa cultures treated with Reversine (the MPS1 inhibitor) ± 14 Gy, demonstrating complete suppression of mitotic death when the SAC is silenced (Fig. 1F).
- Inclusion of new data demonstrating that Reversine completely rescues mitotic death even under conditions of increased HR in 14 Gy irradiated and RAD52 depleted cells (Fig. 3G).
- Demonstration of a direct correlation between mitotic arrest and mitotic death.
    - Fig. 1D, 2C, 3C, 5E, 6C, 8E
    - Extended Data Fig. 1E, 3C, 3I, 4C, 4I, 5E-G, 9E

2. I encourage the authors to look more carefully into the link between mitotic duration, slippage and cell death rates, as it would potentially explain some of their observations. Activation of BAX/BAK in mitosis depends clearly on inhibition and degradation of BCL2 family proteins, most notably BCLX and MCL1, respectively (at least in HeLa). MCL1 acts as a molecular timer in arrested cells, underlining the competing network model put forward by Taylor and colleagues. Interfering with HR may facilitate slippage that promotes cell survival, while inhibiting NHEJ may lead to extended mitotic arrest and death in mitosis (PMID: 18656424; PMID: 29987118; PMID: 23890995; PMID: 25922916, PMID: 29459486). Changes of MCL1 & BIM/NOXA levels or BCLX-phospho status could be assessed in some experimental settings to make a better connection to the BCL2 network in response to the different perturbations tested.

We are thankful for this clear experimental suggestion that improved manuscript quality. To test this directly, we G1/S synchronized 3F Hela with a double thymidine block, released, and irradiated with mock or 14 Gy 6-hours post-release when cells were in G2. We collected mitotic cells from mock or irradiated cultures via mitotic shake-off, as well as non-irradiated asynchronous and G2 enriched cultures. In whole cell extracts prepared form these samples, we observed the following mitotic arrest-dependent outcomes: MCL-1 degradation, BCL-xL phosphorylation, and transient NOXA accumulation. Additionally, Cyclin B1 was not degraded during mitotic arrest following IR. Together, these data are wholly consistent with the pathway described by the reviewer. These new data are included as Fig. 7G and discussed in the associated text.

Except for BAX/BAK DKO cells that had exceptionally long mitotic arrest, mitotic slippage was rare (Fig 7E). While targeting RAD51, RAD52, and/or POLθ yielded some differences in mitotic duration, mitotic death always correlated with significant mitotic arrest, and we observed no significant induction of slippage. We measured mitotic duration in cells treated with the NHEJ inhibitor NU7441. This failed to significantly extend mitotic arrest in the irradiated cells that died in mitosis (Fig. 2C).

3. I would also like to encourage the authors to discuss observations made that suggest G2 checkpoint override after IR damage by inhibition of CHK1 (and ATRi) promotes cell death in HeLa cells (PMID: 18510930, PMID: 22854598), while here the opposite phenomenon is observed. Maybe I miss an aspect that would explain this discrepancy.

We acknowledge the reviewer's comment and understand how these results could appear in contradiction. When examined in detail we do not observe a discrepancy.

The referenced studies use static measurements at fixed time points. Whereas we use dynamic live imaging to measure cell death across the experimental continuum. We observed that ATR inhibition (ATRi) abrogated the G2/M checkpoint and rescued immediate mitotic death in 14 Gy irradiated cells (Fig. 3F, Response Fig. 3A, B). However, immediate mitotic death rescue in 14 Gy + ATRi cells was followed by robust delayed interphase lethality (Fig. 3H, Response Fig. 3A, B). Within our live-imaging experiment, we observe both events (mitotic death rescue, then delayed lethality). In the static ensemble measurements within the referenced papers, the discrepancy between mitotic death and interphase death are not assessed. Both our paper and the referenced papers capture the increased delayed lethality that occurs with IR and either CHK1 or ATR inhibition. Our data does not contradict the previous studies that utilised fixed end points in bulk cell populations, but rather complements them with a single cell resolution of real-time live imaging which uncovered previously masked events.

Additionally, the referenced papers demonstrate that irradiated HeLa under CHK1 inhibition died mainly in G1/S through an ATR/ATM-Caspase-2-dependent pathway (PMID: 18510930). This is consistent with our results where ATR inhibition suppresses mitotic death, to foster delayed interphase lethality. Conversely, in the referenced papers HeLa cultures irradiated with 10 Gy alone perished predominantly in G2/M phase, consistent with our results of immediate mitotic death in G2/M arrest competent cells.

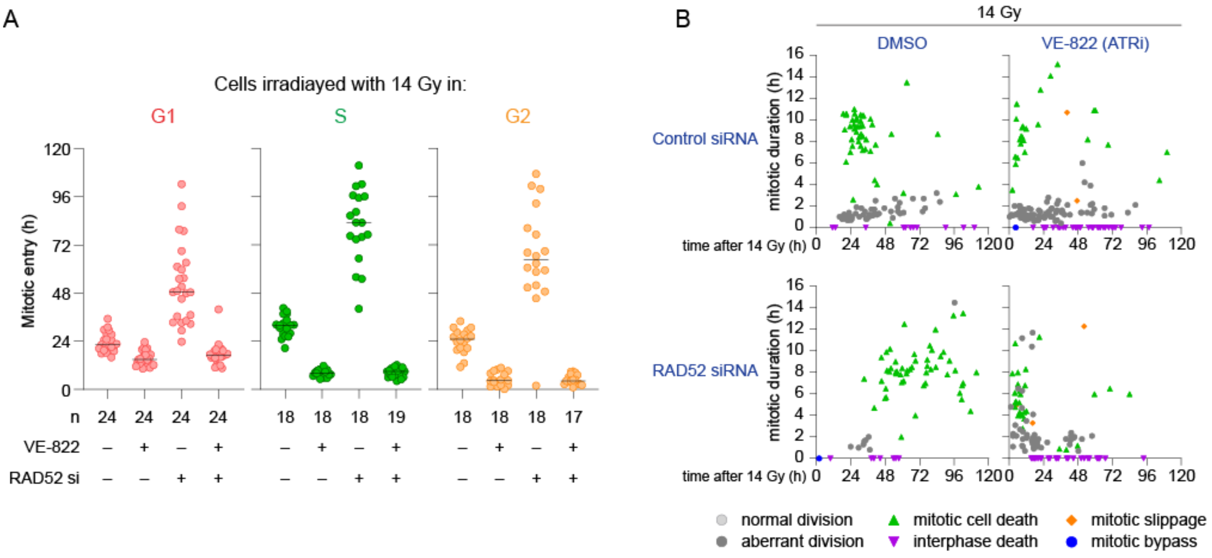

Response Fig. 3. A) Time from irradiation to mitotic entry for data in Fig. 3F. **B)** 3-dimensional plots for data in Fig. 3G.

4a. Moreover, statements like "The innate immune response promotes delayed lethality" are not supported by the data shown, I feel, as there is no experiment conducted that would document that claim. Also, to consider Fludarabine as a selective STAT1 inhibitor is a bit of stretch. At the very best, what the authors show is that escape from mitotic arrest increases cell survival rates in the presence of CIN (an established phenomenon), that promotes inflammatory gene expression downstream of sensing of nucleic acids (also known for a while and confirmed here).

This astute comment prompted our evaluation of STAT1 signalling and its role in delayed lethality. Contrary to Fludarabine, STAT1 depletion had no impact on delayed lethality (measured by Caspase-8 and PARP cleavage) in BAX/BAK double knockout (DKO) cultures refractory to immediate mitotic death (Fig. 7F). Further, STAT1 siRNA enhanced Caspase-8 and PARP cleavage following 14 Gy in wild type background. We therefore removed Fludarabine data from the manuscript. We also edited the text to accurately convey that STAT1 is activated contemporaneously with Caspase-8 cleavage following IR, but that STAT1 is dispensable for delayed lethality. We thank the reviewer for this observation which improved manuscript quality.

4b. Whether this in the end really triggers an "innate immune response" in its actual sense, has not been tested in this study. Maybe the authors could state that they see signs of sterile inflammation, based on their own data, but not more.

Our claim of innate immune response activation was based on cytoplasmic nucleic acid signalling initiating the process. We apologize if this was used incorrectly and thank that reviewer for clarifying. We now use “interferon signalling” or “inflammatory response” and have changed the text throughout.

5. The authors propose that the delayed death in G1 is mediated by caspase-8, based on the use of Z-IETD-fmk, and that this death requires STAT1 signaling, presumably downstream of IFN receptors, using Fludarabine as an inhibitor of STAT1. While I deem Fludarabine to be highly unspecific, exerting a range of effects, I would also dare to say that 40μM of IETD will block other caspases, next to caspase-8; e.g. maybe caspase-2, implicated in mitotic catastrophe since a long time. Also, while all other data is beautifully quantified, here, “relative apoptosis” is shown, which makes it hard to judge what this means in real numbers. So, I suggest to provide some solid experimental evidence and deplete caspase-8 (which HeLa cells tolerate, as they don't express RIP3), to show this death is C8 dependent.

Again, we thank the reviewer for an experimental suggestion which helped improve the paper.

As described in Point 4a, we depleted STAT1 in parental and BAX/BAK DKO HeLa cultures. This demonstrated that STAT1 was dispensable for Caspase-8 cleavage and delayed lethality (measured by PARP cleavage) following IR (Fig. 7F). We also depleted Caspase-8, either alone or on combination with STAT1, in parental and BAK/BAK DKO cells. Irrespective of STAT1, Caspase-8 depletion completely abolished PARP cleavage in irradiated BAX/BAK DKO cultures. This indicating that delayed lethality is mediated by Caspase-8 dependent extrinsic apoptosis. Finally, we also investigated putative roles for Caspase 2 in mitotic death, and we note Caspase 2 depletion does not affect immediate mitotic death in irradiated HeLa cells (Extended Data Fig. 9C-E).

6. Last but not least, the authors build their claims on "suppression of innate immunity by HR" on the induction of a number of ISGs mRNAs when HR is blocked, but they never show that these transcripts are translated into protein. Given that the peak in mRNA of ISGs is seen at times where cells have fully activated caspases, that can block translation, e.g. by cleaving translation initiation factors, there is reason to doubt that these proteins are expressed and secreted in order promote sterile inflammation, but I am happy to see data that argues against my point of view.

Again, we thank the reviewer for the pertinent experimental suggestion. As requested, we examined ISG protein expression following IR. We are pleased to report that the protein products of IFIT1 and ISG15 are highly upregulated at 3- and 5-days post-IR in the presence of ATR inhibitor, VE-822 (Fig. 8B). This is consistent with increased ISG expression and protein production in HR-deficient cells following IR.

7. Finally, it would be also nice, if some of the key findings are confirmed in an additional model cell line, not just the overall trend, as shown in figure 1H.

We attempted several key experiments in the 3F HT1080 6TG cell line. All outcomes were consistent with our observations in 3F HeLa. Specifically:

- HT1080 6TG displayed the same relationship between cell cycle phase at IR and cell death mode as 3F HeLa (Extended Data Fig. 1F).
- BRCA2 or PALB2 depletion suppressed mitotic arrest and immediate mitotic death in G2 irradiated 3F HT1080 6TG cultures (Extended Data Fig. 4B, C and 7D).
- WAPL deletion rescued cohesion fatigue and mitotic death in 14 Gy irradiated 3F HT1080 6TG cells (Extended Data Fig. 7D, F).
- Co-targeting WAPL and HR enhanced first mitotic survival in G2 irradiated 3F HT1080 6TG cells more than targeting WAPL or HR alone (Extended Data Fig. 7D).

Point by Point rebuttal. Please see the below responses in blue.

Reviewer #1:
The authors have done a substantial amount of work to strengthen the conclusions that HR intermediates trigger mitotic cell death after IR. The exact mechanism can be a subject for future study. This is important work that links DNA repair mechanism to inflammatory signaling.

We thank the reviewer for their efforts in assisting with our manuscript.

Reviewer #2:
In their revised manuscript entitled 'Homologous recombination promotes mitotic death to suppress interferon production following genome damage', Szmyd et al. show that specific DNA repair pathways can impact on whether a cell encountering DNA damage will arrest in a subsequent mitosis, or not. In turn, this decision can impact on the strength of cGAS- and MAVS-dependent innate immune signalling. Overall, the authors have satisfied all my essential concerns, and have put together a highly compelling study that should have considerable impact on many fields relating to genome instability.

We thank the reviewer for their helpful comments what have improved our manuscript.

Whilst not essential, I highly recommend two minor points to be considered by the authors.

1. They should mention that mitotic arrest and cell death after DNA damage were already reported by (at least) three studies, including one of their own: PMIDs 24080495, 31530811, 32877684)

We agree and have added these references, numbered 3-5 to the second sentence in the introduction.

2. I'm not entirely sure that they have excluded that WAPL may act through the spindle checkpoint. The experiment the authors have added (Ext data Fig. 7I) used a very high amount of nocodazole, which may generate a stronger SAC stimulus than DNA damage by-products. Whilst it's fine if this remains as is for this manuscript, ideally they would use lower nocodazole concentrations, which can generate weaker SAC stimuli.

We appreciate the reviewer's concern. We note that in this experiment the SAC was inhibited by treatment with the AURKB inhibitor Hesperadin, suggesting the SAC resulting from high nocodazole treatment can be overcome when signalling in this pathway is targeted. Because the reviewer is fine with the result remaining in the paper, we have elected to not change the data. We will keep this in mind for future experiments.

Reviewer #3:
My concerns have been met. I would still remove the reference to mitotic catastrophe at the end of the intro, as also a second reviewer is not really happy with this terminology (line 88), maybe "different modes of cell death" or cell death signalling events...

We agree. The term was edited to "cell death outcomes". Additionally, thank you to the reviewer whose comments throughout the revision process improved our manuscript.
